# Early tetrapod neck mobility modelled using an extant analogue

**DOI:** 10.1038/s41586-026-11158-3

**Published:** 2026-10-14

**Authors:** Dana E. Korneisel, Cassandra M. Donatelli, Linfang Han, Thomas A. Stewart, Andrew L. Traynor, Benjamin K. A. Otoo, Marcos Martinez, Tetsuto Miyashita, Emily M. Standen, Hillary C. Maddin

**Affiliations:** 1 Earth Sciences Department, Carleton University, Ottawa, Ontario Canada; 2 School of Engineering and Technology, University of Washington, Tacoma, WA USA; 3 Department of Biology, University of Ottawa, Ottawa, Ontario Canada; 4 Department of Biology, The Pennsylvania State University, State College, PA USA; 5 Canadian Museum of Nature, Ottawa, Ontario Canada; 6 Present Address: Department of Organismal Biology, Uppsala University, Uppsala, Sweden

**Keywords:** Palaeontology, Biological techniques

## Abstract

Tetrapods arose from lobe-finned fishes with a notochord-dominated axial column^1–3^. During this transition, the skull became separated from the shoulder girdle^4–6^, giving rise to a neck region. This head–shoulder separation has generated hypotheses suggesting that neck mobility increased during the origin of tetrapods, facilitating the ecological transition towards more terrestrial habitats^4,5,7–9^. However, incompletely co-ossified vertebral components^10^ have posed a challenge to modelling the mobility of these early tetrapod necks. Here we used material tests on the similarly configured axial columns of sturgeon to predict the capacities of the tetrapod neck region for lateral flexion, extension and rotation across the tetrapod stem. Our model indicates that the soft tissue-dominated axial column was surprisingly rigid, despite lacking co-ossified vertebral components. We estimate that most stem tetrapods could move their heads less than 6° laterally and less than 3° dorsally but could not rotate them. Notably, mobility did not shift in conjunction with the head–shoulder separation that occurred between the origin of tristichopterids (represented by *Eusthenopteron*) and that of more crownward stem tetrapods. Therefore, mobility in the tetrapod neck did not serve as a functional driver of head–shoulder separation. Instead, forelimb-driven locomotion, implicated in early tetrapods^11–14^, provides a better functional explanation for the head–shoulder separation.

## Abstract (web summary)

Biomechanical analysis of several early tetrapods using sturgeon as an analogue revealed that early tetrapods had limited neck mobility despite separation of the head and shoulders.

## Main

Since the head and trunk first became anatomically distinct in the earliest vertebrates, the interface between these two body regions has undergone a series of evolutionary transformations^3,5,9,15^. These include a distinct vagal neural crest population^16–19^, neck musculature (that is, cucullaris)^20–22^, specialized anterior spinal nerves (the accessory and hypoglossal nerves)^23–25^ and shoulder girdle^6,15,26,27^. During all these transformations, full head motion (as opposed to intracranial motion) was never independent of trunk motion^5,6,9,28,29^. However, the skull–trunk linkage underwent a marked change during the origin of tetrapods when the skull and shoulder girdle separated, giving rise to a distinct neck region unique to tetrapods^1,5,6^. Documented in key stem tetrapods such as *Acanthostega*^4,8^ and *Tiktaalik*^9^, this head–shoulder separation has given rise to the hypothesis that neck mobility increased markedly through an increase in flexion, extension or rotation across the new neck region during the origin of tetrapods as they began to inhabit more terrestrial habitats^4,7–9^. The predicted increase in neck mobility has not been functionally tested.

**Fig. 1:**
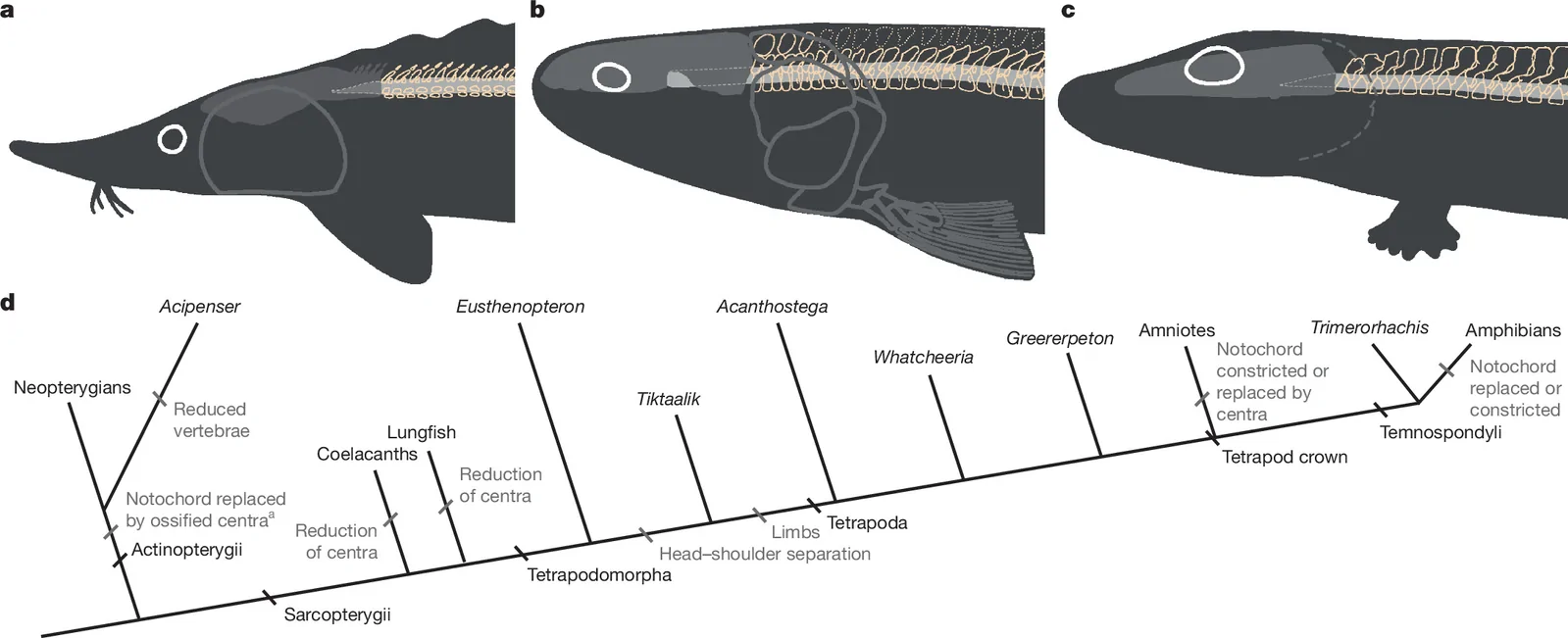
Evolution of the axial column during the origin of tetrapods. **a**, The anterior axial column of the lake sturgeon (*A. fulvescens*) showing the opercular bones; only the external operculum edge is outlined. Vertebral arches are shown in beige. Select skull elements are shown in dark grey, with opaque bone and translucent cartilage; the notochord is represented in pale grey and the white circle indicates the approximate position of the eye. **b**, The anterior axial column of *E. foordi* showing the ancestral tetrapodomorph condition, based on previously published studies^3,30^. Supraneurals posterior to the third supraneural are not illustrated but are presumed to be present anterior to the dorsal fin, as in other fishes. **c**, The anterior axial column of *A. gunnari* showing the limbed tetrapodomorph condition, based on previously published studies^2,63,64^. The dashed grey line indicates the posterior edge of the lateral skull. *Eusthenopteron* (**b**) and *Acanthostega* (**c**) are extinct. **d**, Phylogenetic relationships among the taxa examined in this study with clades denoted in black and relevant anatomical shifts in grey. ^a^Several secondary losses of central ossification occur in actinopterygians.

**Extended Data Fig. 1:**
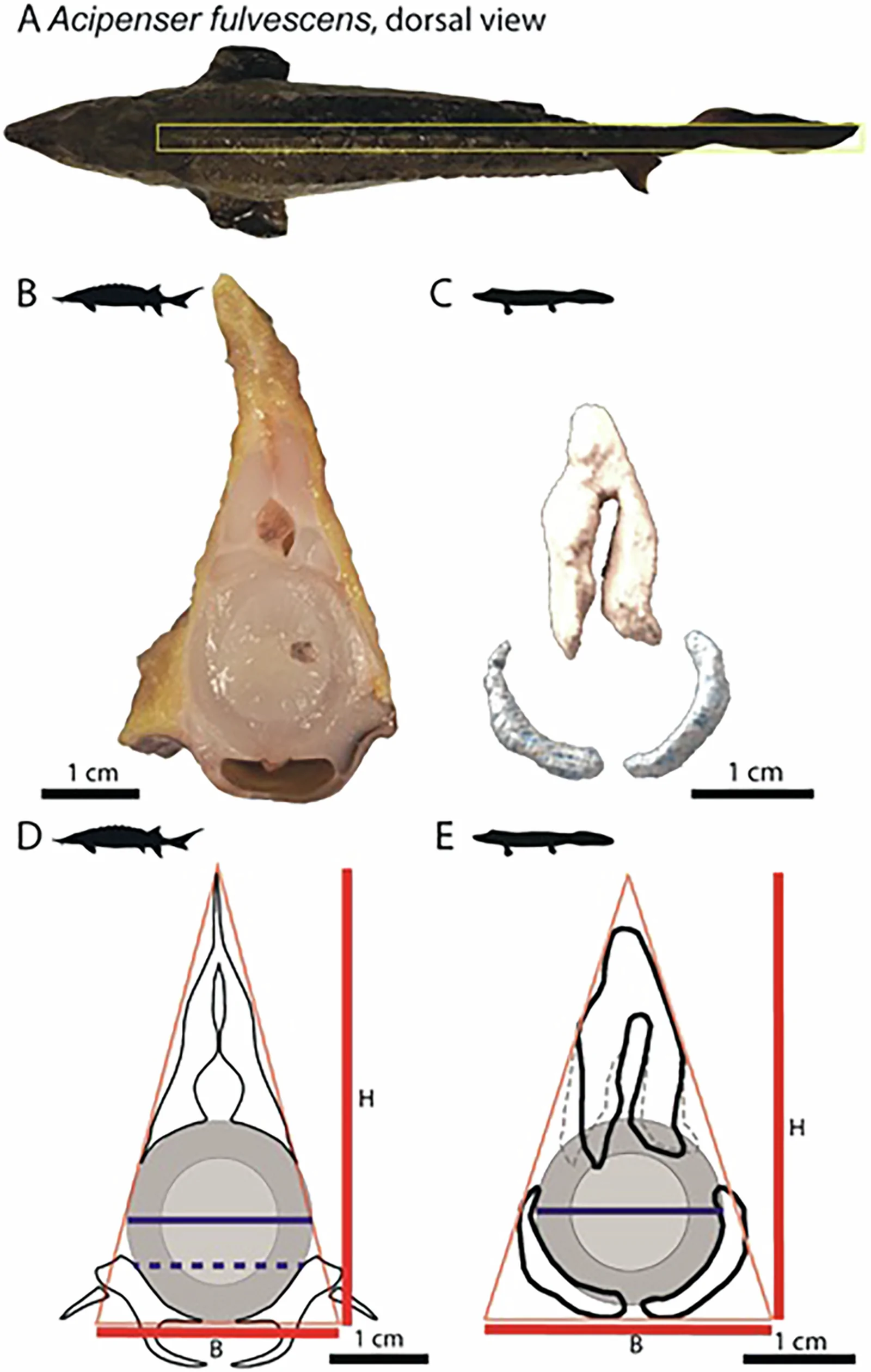
Measurements for calculation of second moment of area and polar moment of area in sturgeon. *Acipenser fulvescens* (A, B, D), and stem tetrapods (C, E), represented here by an exemplar, *Tiktaalik roseae*. **A**. *A. fulvescens* individual in dorsal view with area dissected for testing marked in yellow. **B**. *A. fulvescens* anterior axial column in cross-sectional view. **C**. Surface rendering of the anterior-most well-preserved vertebra of *T. roseae* photographed in cross-sectional view. **D**. Line drawing of idealized sturgeon vertebra in cross-sectional view with measurements marked. **E**. Line drawing of *T. roseae* vertebra 4 in cross sectional view with measurements marked. An approximated retro-deformation of the neural arch pedicles is indicated by a grey dashed line. Blue lines indicate notochord diameter (solid) and intercentral span (dashed in sturgeon where intercentral span is only 96% of notochord diameter). Red lines indicate column measurements, B = base, H = height. Image in **c** reproduced from ref. ^41^, PNAS, under a creative Commons licence CC BY-NC-ND 4.0.

Stem tetrapods have an axial column dominated by the soft tissue notochord, which they retain throughout adulthood^1–3,30,31^ (Fig. 1). The notochord is surrounded by distinct cartilaginous or bony arches that lack bone-to-bone joints^32,33^ (Fig. 1b,c and Extended Data Fig. 1). This condition predates the evolution of the vertebral column characteristic of most adult crown tetrapods in which the notochord is mostly replaced by bony centra. In such an axial column, each vertebral centrum is generally fused to a neural arch, and the vertebrae articulate with one another through inter-central and zygapophyseal joints^10^. Stem tetrapods lack these stiffening features, despite their hypothesized functional advantage in supporting the axial column for survival in increasingly terrestrial habitats^4,7–9^. These anatomical differences mean that the axial columns of stem tetrapods are not a joint-based system like those of crown tetrapods, making the ‘soft’ axial columns of stem tetrapods challenging to model. No modern tetrapods have an anatomically or functionally comparable axial skeleton^10^. To generate constrained estimates of the functional capability of the stem tetrapod neck, we sought an alternative model using phylogenetically independent but anatomically comparable extant analogues for which material testing can be performed.

**Extended Data Fig. 2:**
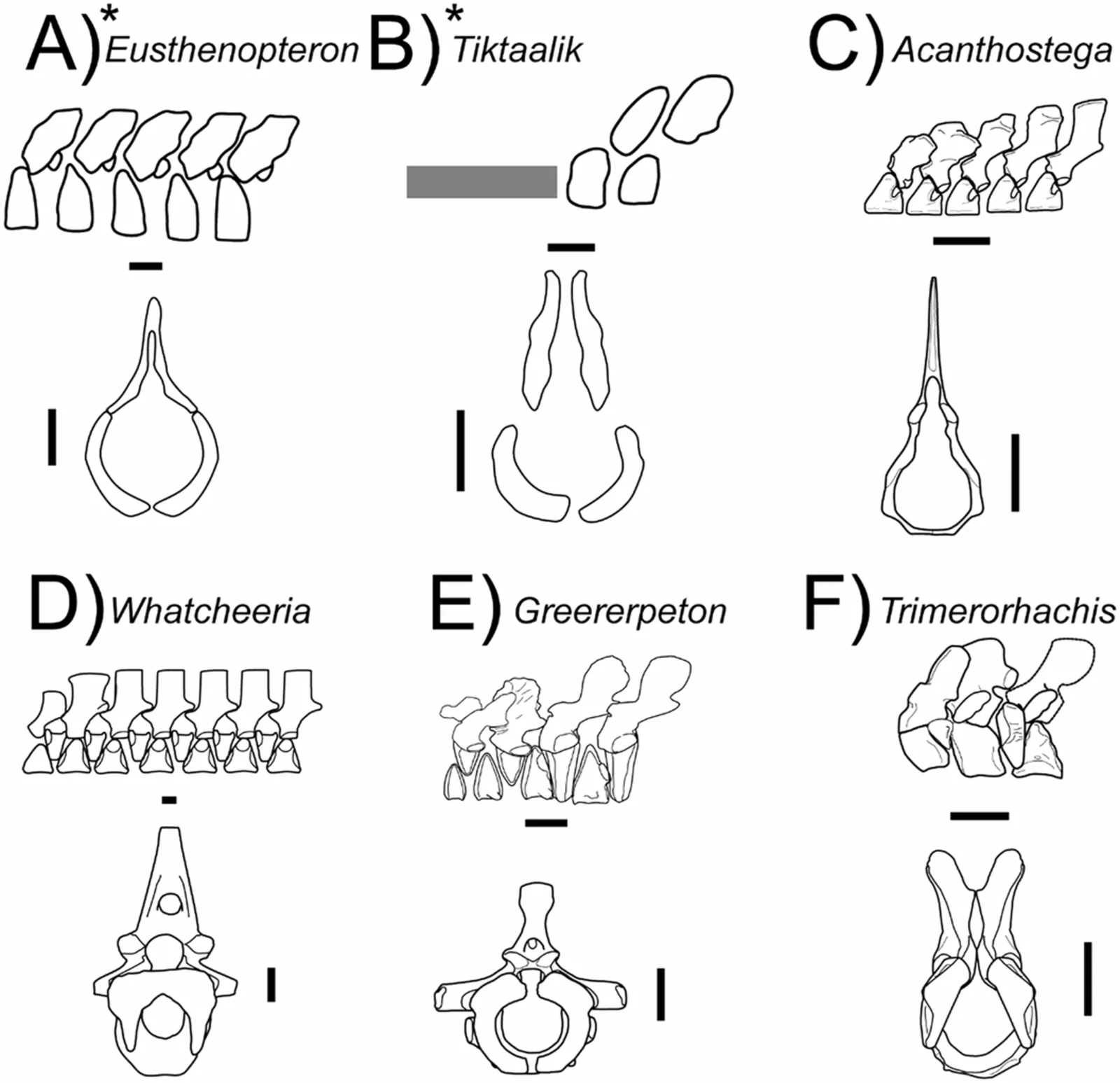
Vertebral reconstructions for extinct taxa considered for this study. Taxa with a highly restricted notochordal canal were deemed too anatomically dissimilar from the analogue for mobility estimation. A. *Eusthenopteron foordi*: from Andrews and Westoll (1970), input from Hitchcock, 1995 also considered in this study. B. *Tiktaalik roseae*: C. *Acanthostega gunnari*: modified from Coates (1996), vertebra in anterior view is equivalent to the posteriormost cervical. D *Whatcheeria deltae*: modified from Otoo et al. (2021) by removal of the first centrum component from the original reconstruction following Korneisel et al. (2025)’s observation that this appears to have been a misplaced pleurocentrum, vertebra in anterior view is a posterior cervical reconstructed from isolated vertebral components. E. *Greererpeton burkemorani*: modified from Godfrey (1989a), vertebra in anterior view is V5. F. *Trimerorhachis insignis*: based on Case (1911), 1935, Shiskin (2000), and Pawley (2000), vertebra in anterior view is reconstructed from isolated centra and posterior trunk neural arches. *measurements in this study were not taken from this reconstruction. *Eusthenopteron* included supraneurals per Hitchcock (1995) and *Tiktaalik* measurements were taken directly from CT rendering. All scale bars 1 mm.

We identified the lake sturgeon (*Acipenser fulvescens*) as the best available model for the soft axial column of stem tetrapods. Sturgeon retain an intact notochord into adulthood^34,35^ and have comparable body dimensions to stem tetrapods (Extended Data Figs. 1 and 5). The notochord-dominated axial column of sturgeon, a secondary adaptation in this lineage, has an anatomical configuration similar to that of stem tetrapods in terms of the overall proportions of vertebral arches to the notochordal canal in cross-section (Extended Data Figs. 1 and 2). The arches are joined by unmineralized connective tissues rather than by zygapophyseal joints^34,35^ and the ventral vertebral components are multipartite (basidorsals and basiventrals in sturgeon; pleurocentra and intercentra in stem and early tetrapods)^34^. Some anatomical details of sturgeon vertebrae differ from those of their counterparts in stem and early tetrapods. Two examples are that sturgeon have supraneurals and that the basidorsals and basiventrals are placed dorsoventrally across the notochord from one another^34^, whereas the intercentra and pleurocentra in stem and early tetrapods are staggered relative to one another but both ventrolateral to the notochord (Extended Data Fig. 2)^34^. Acknowleging these minor differences, we performed a series of material tests on the axial column and notochord of sturgeon specimens and measured lateral flexion stiffness, extension stiffness and torsional (rotational) stiffness. We then applied these stiffness values to the axial column dimensions of stem and early tetrapod taxa to determine the column rigidity and estimate neck mobility in these species.

## The sturgeon axial column model

The mobility of a notochord-dominated axial column is limited by its material properties rather than by zygapophyseal articulations. This type of column is composed of soft tissue (notochord), hard tissue (cartilage or bony arches) and the connective tissues that unite them, all of which contribute to the material properties of the column. Notochord stiffness has been measured in hagfish^36^, salmon embryos^37^ and tadpoles^38^, and its angular stiffness and internal pressure have been calculated in sturgeon during swimming^35^ (Supplementary Table 3). In hagfish, notochord stiffness alone accounts for most of the body stiffness^36^. By contrast, reduced ossification of the anterior-most vertebrae in some dragonfish provides the notochordal column with much greater mobility than the rest of the column with ossified centra^39^; thus, the tissues surrounding the notochord may considerably increase the stiffness of the whole notochordal axial column in non-cyclostomes. To test their relative contributions to body stiffness, we measured the stiffness of both the isolated notochord and the whole column for our range-of-motion estimates of the early tetrapod axial column.

In sturgeon, the whole axial column approximates axial mobility better than does an isolated notochord. Our data indicate that notochord stiffness is not the limiting factor for axial column mobility in sturgeon, as the stiffness of the whole column (with intact mineralized arches and connective tissue) is considerably higher than that of the notochord alone (Table 1 and Supplementary Fig. 4). This high column stiffness is not attributable to hard stops or joint interactions between the arches, as we did not observe any such interactions or regionalization of bending during testing. Only negligibly small forces and torques were required to bend and twist the notochord compared with the whole column (Extended Data Table 1, Supplementary Table 4 and Supplementary Fig. 4). Therefore, we used whole-column stiffness to estimate the flexural rigidity of the similarly configured necks of stem tetrapods (Methods).

**Table 1:** Bending and torsional stiffness values

Measurement	*E* or *G* (GPa)
**Lateral bending** (***E***)	
Notochord only	0.45–0.52
Whole column	1.51–1.82
**Dorsoventral bending** (***E***)	
Notochord only	0.55–1.07
Whole column	0.95–1.35
**Torsion** (***G***)	
Notochord only	2.11 × 10^−4^–2.44 × 10^−4^
Whole column	2.1–3.95

Values were computed using materials tests on *A. fulvescens* (*n* = 10). *E*, Young’s modulus or bending stiffness; *G*, torsional stiffness.

**Extended Data Fig. 3:**
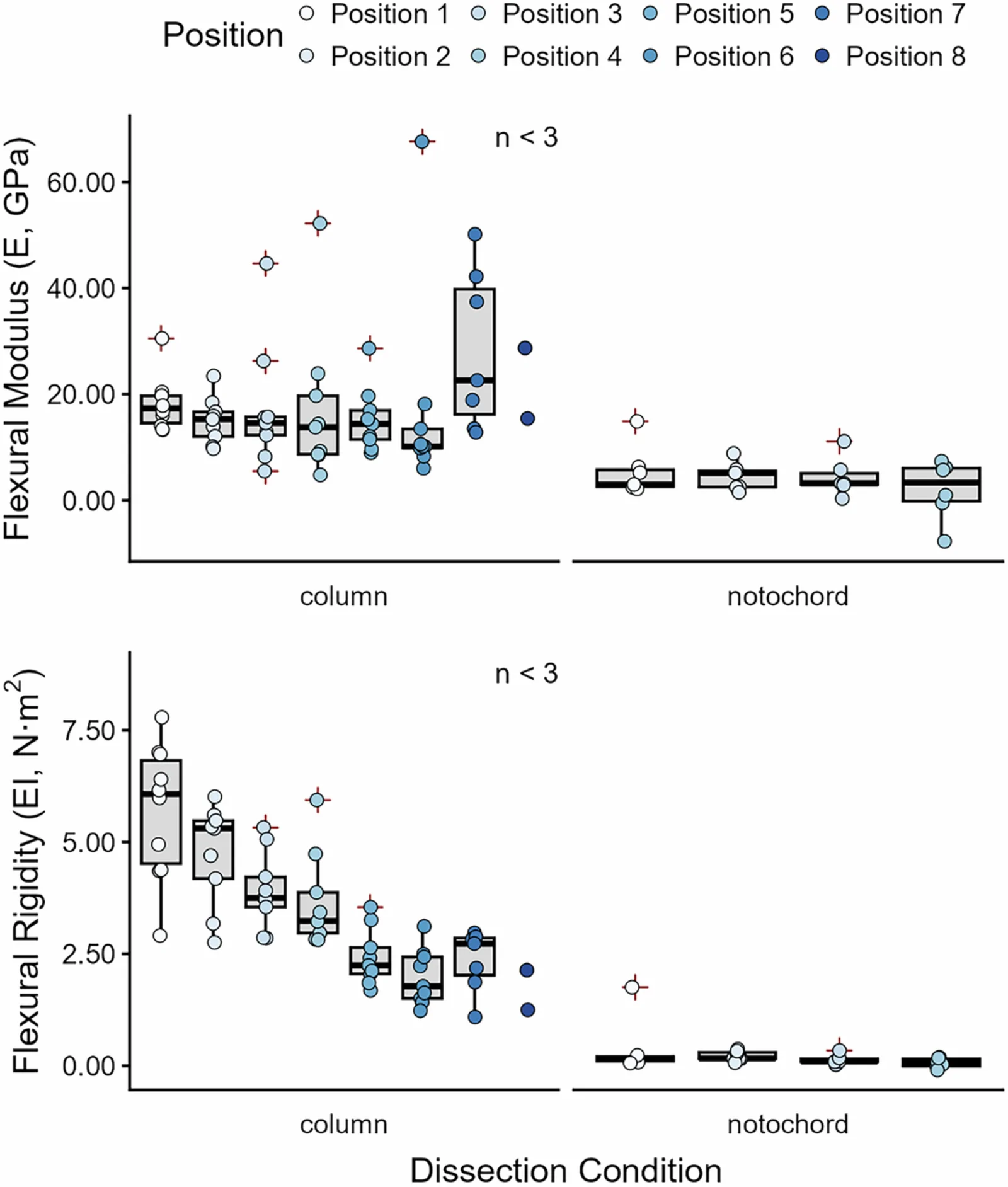
Lateral bending results from 3-point bending trials of the lake sturgeon, *Acipenser fulvescens*. Flexural modulus is the same as Young’s modulus. Colour of the jittered point represents position along the body. The solid line within the boxplot represents the median and the upper and lower limit of the box represents the first and third quartiles (25^th^ and 75^th^ percentiles). The distance between first and third quartiles is defined as the interquartile range (IQR). Whiskers extend to the furthest value that is 1.5x IQR. Points marked with a red cross indicate points outside of the IQR. No median or quartiles are presented for column lateral bending at position 8, as n = 2. Remaining sample sizes are as follows: Column position 1, n = 10; Column positions 2-6, n = 9; Column position 7, n = 8; Notochord position 1, n = 9; Notochord positions 2 & 3, n = 7; Notochord position 4, n = 6. Source data

**Extended Data Fig. 4:**
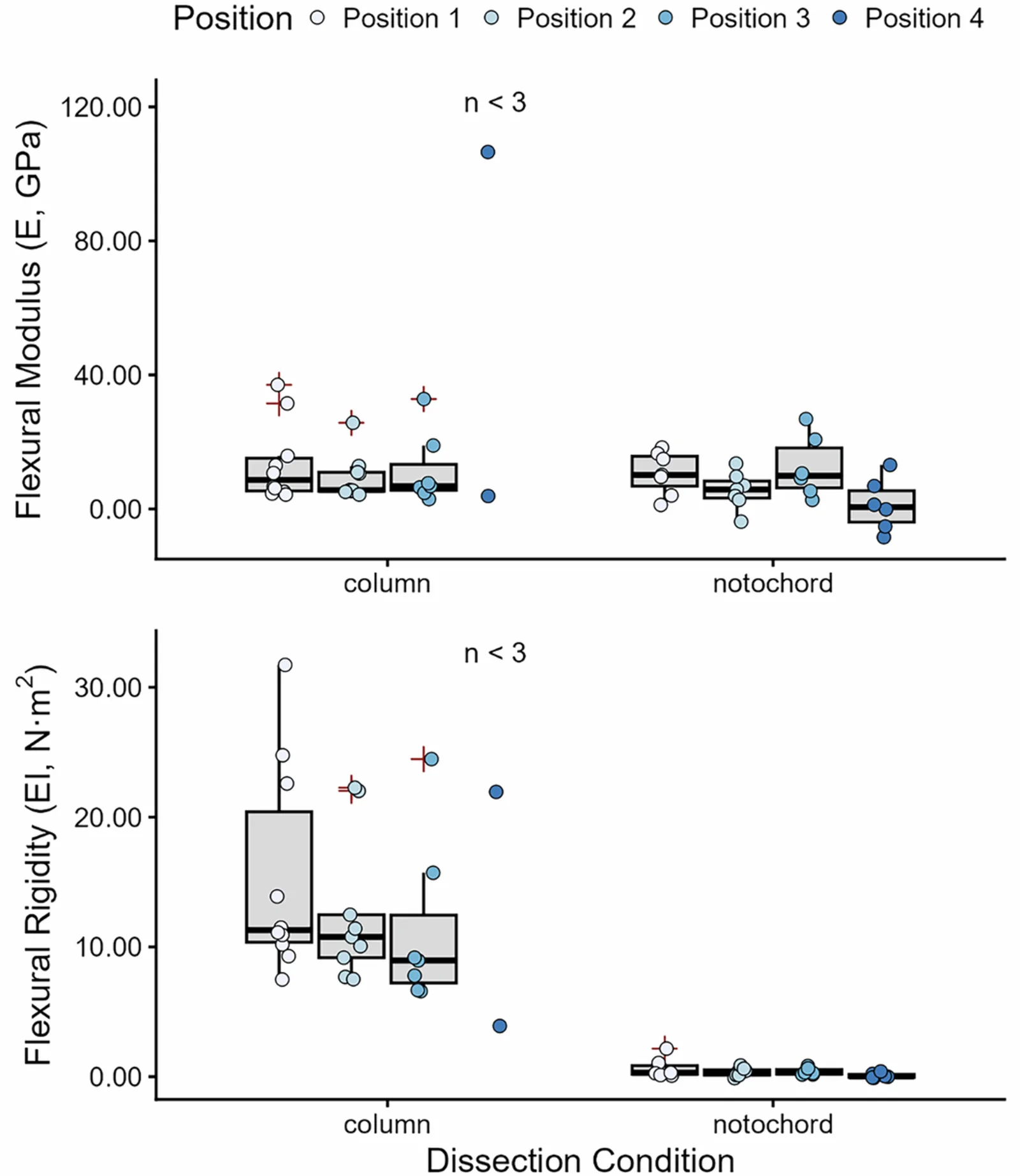
Dorsoventral bending results from 3-point bending trials of the lake sturgeon, *Acipenser fulvescens*. Flexural modulus is the same as Young’s modulus. Colour of the jittered point represents position along the body. The solid line within the boxplot represents the median and the upper and lower limit of the box represents the first and third quartiles (25^th^ and 75^th^ percentiles). The distance between first and third quartiles is defined as the interquartile range (IQR). Whiskers extend to the furthest value that is 1.5x IQR. Points marked with a red cross indicate points outside of the IQR. No median or quartiles are presented for column dorsoventral bending at position 4, as n = 2. Remaining sample sizes are as follows: Column position 1, n = 10; Column position 2, n = 9; Column position 3, n = 6; Notochord position 1, n = 9; Notochord position 2 & 3, n = 7; Notochord position 4, n = 6. Source data

**Extended Data Fig. 5:**
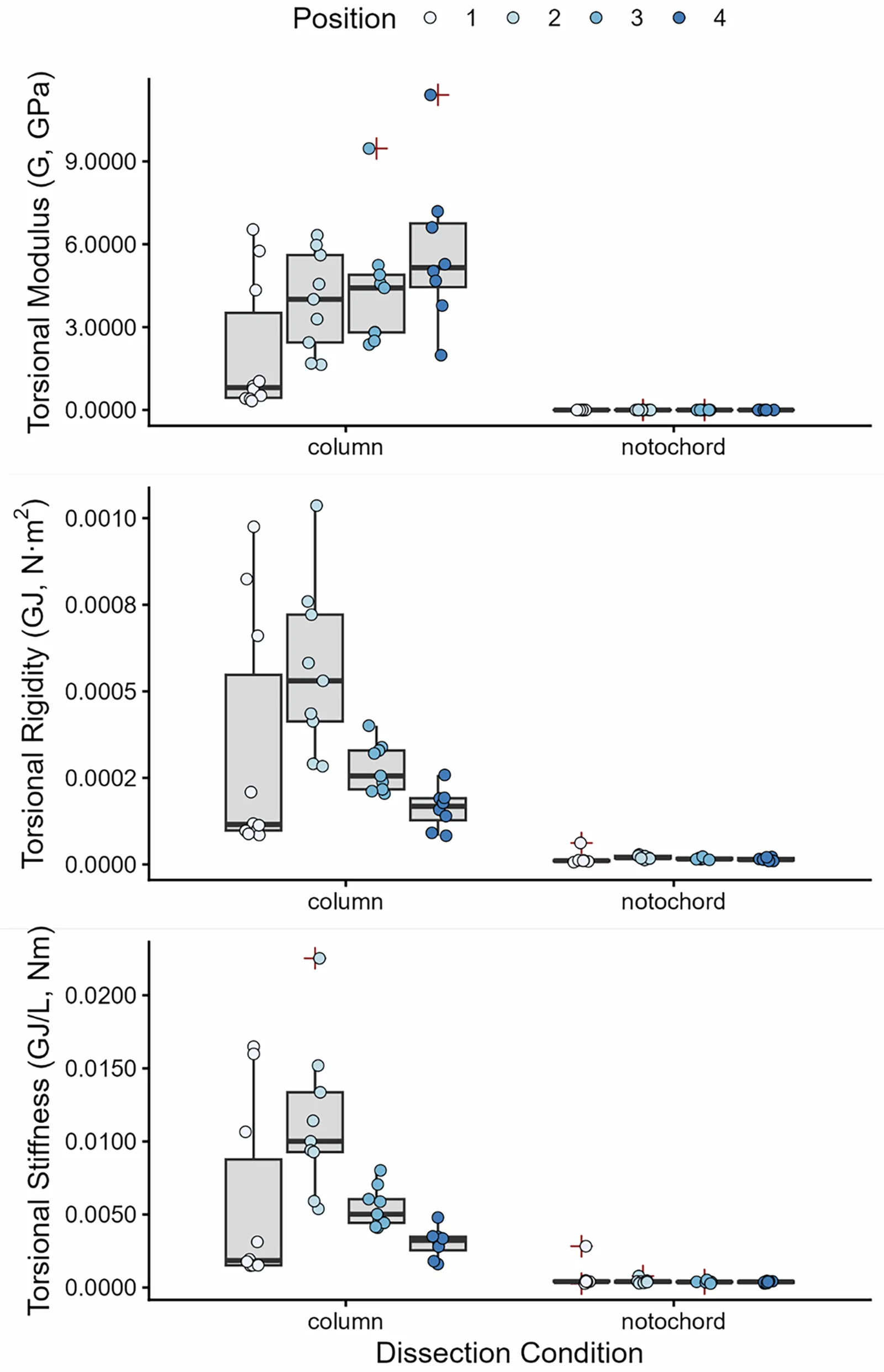
Torsion testing results for the lake sturgeon, *Acipenser fulvescens*. Colour of the jittered point represents position along the body. The solid line within the boxplot represents the median and the upper and lower limit of the box represents the first and third quartiles (25^th^ and 75^th^ percentiles). The distance between first and third quartiles is defined as the interquartile range (IQR). Whiskers extend to the furthest value that is 1.5x IQR. Points marked with a red cross indicate points outside of the IQR. Column position 5 is excluded due to n = 1. Remaining sample sizes are as follows: Column positions 1-3, n = 9; Column position 4, n = 8; Notochord position 1, n = 9; Notochord position 2 & 3, n = 7; Notochord position 4, n = 6. Source data

In lateral flexion, the sturgeon’s posterior precloacal and tail regions of the axial column are less stiff than the anterior-most section (Extended Data Fig. 3 and Supplementary Table 5), located just posterior to the fused occipital vertebrae (these are unique to sturgeon in our dataset; Fig. 1). Stiffness was consistent along the column for the other measurements (Extended Data Figs. 3–5 and Supplementary Tables 6 and 7). To account for this variation for sturgeon, we used the Young’s modulus from the anterior-most testing position for lateral flexion (Table 1), as this area is most comparable anatomically to the neck region in stem tetrapods.

## Neck mobility is highly constrained

To estimate tetrapod neck mobility, we selected five stem tetrapods (*Eusthenopteron foordi*^3,30,31,40^, *Tiktaalik roseae*^41^, *Acanthostega gunnari*^2^, *Whatcheeria deltae*^42^ and *Greererpeton burkemorani*^43^) and one crown tetrapod (*Trimerorhachis insignis*^44–47^) for which axial column dimensions (Extended Data Fig. 1) are measurable. The vertebral components in these taxa are sufficiently preserved to enable the reconstruction of the anterior-most axial column and the estimation of the body cross-sectional area at the neck. On the basis of the material tests using sturgeon specimens, we estimated that neck mobility is constrained strongly by the axial column in these taxa.

### Lateral flexion and extension

To estimate lateral flexion and extension (head elevation), we first determined the second moment of area for the anterior axial column (equation (3); Methods) and then calculated the force (*F*) required to achieve a given deflection (*δ*) (equation (8); Methods).

$$F=\frac{\delta 3{EI}}{{L}^{3}}$$

where *E* is Young’s modulus (Pa) calculated from material tests of sturgeon specimens, *I* is the second moment of area (m^4^) of the relevant section of the axial column and *L* is the length (m) of the cervical region or its equivalent. We next calculated the muscle cross-sectional area required to generate these forces, using the known pressures for striated muscles in vertebrates (Methods). These muscle areas were capped for each taxon at half the area of an oval fitted around the occiput to provide an absolute maximum muscle area available for lateral flexion (area on one side) and extension (combined dorsal area of both sides; Supplementary Fig. 3). The resulting estimates of the maximum range of motion represent the angles of deflection produced by the maximum force from the corresponding muscle area.

**Fig. 2:**
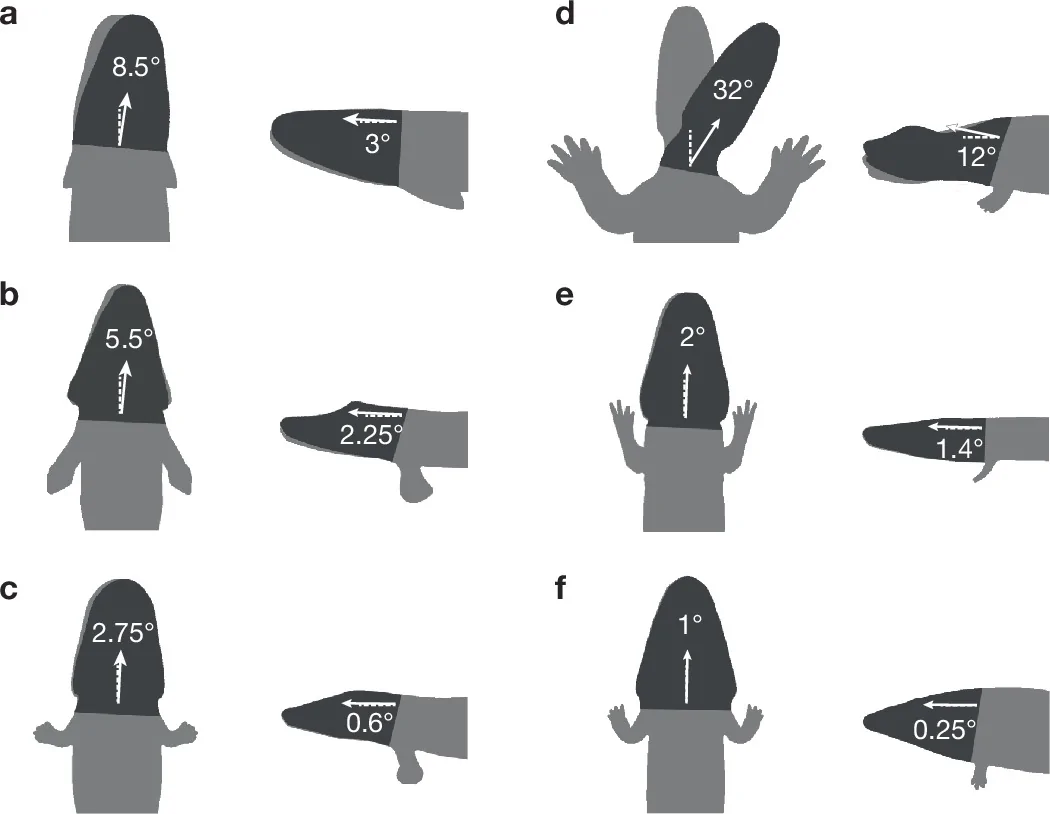
Estimates of lateral flexion and extension. **a**–**f**, Range-of-bending estimates in the neck (or analogous) region for *E. foordi* (**a**), *T. roseae* (**b**), *A. gunnari* (**c**), *W. deltae* (**d**), *G. burkemorani* (**e**) and *T. insignis* (**f**), as limited by axial column stiffness. Silhouettes are not drawn to scale relative to one another. Calculated estimates of lateral flexion and extension, along with neck length data, are provided in Extended Data Table 2.

Our modelling revealed only modest neck mobility (Fig. 2) in the tetrapodomorphs *Eusthenopteron* (lateral flexion of 8.5°) and *Tiktaalik* (lateral flexion of 5.5°)—the earliest-diverging stem tetrapods in our dataset. Except for *Whatcheeria*, stem tetrapods were generally not capable of lateral neck flexion of more than 8.5° or neck extension of more than 3°. Among early tetrapods, *Acanthostega*, *Greererpeton* and *Trimerorachis* showed even lower estimates for lateral flexion (<3°) and extension (<1.5°) than their fish outgroups. We generated these estimates for the notochord-dominated anterior-most region of the axial column^30,41^, where the column is much taller dorsally than it is wide in cross-section (Extended Data Fig. 2c). The column height was at least 166% of the column width in all tetrapods except *Tiktaalik* and *Greererpeton* (155% in *Tiktaalik*; only 128% in *Greererpeton*). In *Eusthenopteron*, this height was due to the presence of supraneurals^3^ (not illustrated in Extended Data Fig. 2a), whereas in other stem and early tetrapods, it was due to the relatively tall neural arches (Extended Data Fig. 2c,d,f). No matter whether height comes from supraneurals or tall neural arches, our estimates generally suggest that taller axial columns have less mobility than do shorter ones.

In a notochord-dominated axial column, the length of the bending region also affects mobility, as the force required to achieve lateral flexion or extension decreases as a cubic function of length (see equation (8); Methods). This inverse cubic function partly explains the low estimates of neck mobility in *Acanthostega* and *Greererpeton* and the particularly short-necked *Trimerorhachis* (1° of lateral flexion and <1° of extension with only three neck vertebrae). Conversely, we attribute the surprisingly high estimated neck mobility in *Whatcheeria*, in part, to its long neck (Extended Data Fig. 2d). Our modelling suggests that *Whatcheeria* could achieve up to 32° of lateral flexion and 12° of extension, which exceed our estimates for other early tetrapods by approximately tenfold (lateral flexion) and eightfold (extension). It has a substantially longer neck (89% of the skull length) than other early tetrapods (34.2% of the skull length in *Acanthostega* and 37.6% in *Greererpeton*)^42^. Thus, *Whatcheeria* seems to be an outlier among early tetrapods in terms of both neck length and mobility; others generally had a highly limited range of head motion.

### Rotation

To estimate the torques in the axial column required for head rotation, we used the modulus of rigidity (*G*) averaged from the material tests in sturgeon using equations (5)–(7) (Methods) and determined the polar moments of area (*J*) based on measurements obtained from the axial columns of stem and early tetrapods using equation (5) (Methods):

$${G}{J}=\frac{{TL}}{\theta }$$

where *T* is the maximum torque (Nm), *L* is the gauge length (m) and *θ* is the angle at maximum torque (degrees). We calculated torque (*T*) at small angles (5–10°) and found that the column required very high torques to achieve any rotation (≥200 Nm for 5°; Extended Data Table 1).

These high torque estimates suggest that large head turns are infeasible across the tetrapod stem, even in taxa with distinct atlas and axis vertebrae (notably, *Greererpeton* among the taxa examined here)^2,10,42,43,46^. We calculated the torques required to produce small 5° head turns in each taxon because the current knowledge of muscular anatomy is insufficient to estimate the maximum rotation achievable by the axial musculature (Extended Data Table 1). Among the taxa examined here, *Tiktaalik* required the lowest torque (200 Nm) for a 5° head rotation (Extended Data Table 1). This estimated torque is considerable given the limited length of the musculoskeletal elements that form the lever arm (for example, the cervical ribs). All other examined taxa, including *Eusthenopteron*, would require even higher torque (314–746 Nm) than that required in *Tiktaalik* for the same degree of rotation (Extended Data Table 1).

## Discussion

### Factors influencing axial column bending

Variations in the maximum lateral flexion and extension estimates for stem and early tetrapods are correlated with differences in the body proportions, especially the neck length, of the species analysed. The estimated maximum lateral flexion values of *Trimerorhachis*, the taxon with the shortest neck (<3 cm, 19% of its skull length; Supplementary Table 5), and *Whatcheeria*, the taxon with the longest neck (nearly 20 cm, 89% of its skull length; Supplementary Table 5), differ by 31°. However, the overall neck length and the number of vertebrae in the neck region are similar across most stem tetrapods (*Eusthenopteron*, *Tiktaalik*, *Acanthostega* and *Greererpeton*). Although *Eusthenopteron* lacks a distinct cervical region defined by the area between the skull and shoulder girdle, an equivalent region can still be identified in the anterior-most axial column by anatomically distinguishable vertebrae (that is, ribless centra or broad supraneurals, depending on the specimen)^3,30,40^. We measured the axial column length in this region to model theoretical mobility if the region were free (Extended Data Table 2 and Extended Data Fig. 2). The lateral flexion capacities of the axial columns of *Eusthenopteron* and *Tiktaalik* exceed those of *Acanthostega* and *Greererpeton* by two- to threefold (Fig. 2 and Extended Data Table 2), despite comparable neck lengths. These differences seem to result from variations in the geometries of both the axial column and body cross-sections (Extended Data Fig. 2 and Supplementary Fig. 3).

Axial column geometry influences the moment of inertia (*I* in equations (8) and (9); Methods), whereas the body cross-sectional area limits the maximum muscle area (Methods and Supplementary Fig. 3). The ratio of vertebral width to height affects *I* and, therefore, the column mobility, especially in extension. In *Tiktaalik*, the vertebrae are nearly as tall as they are wide at the base and this enables 2.25° of extension (Extended Data Figs. 1c,e and 2b; similar to *Greererpeton*, Extended Data Fig. 2e). Vertebral height is greater in other taxa (up to 2.17 times the width in *Acanthostega*; Extended Data Table 1 and Extended Data Fig. 2c) and extension is correspondingly lower (0.6° in *Acanthostega*; Fig. 2 and Extended Data Table 2). To further assess how vertebral proportions affect bending, we calculated bending for a theoretical 10 cm-long neck with the column of each taxon scaled to the same width. We found not only that tall and narrow vertebral proportions are indeed restrictive, especially in *Acanthostega*, but also that the rectangular profile of *Trimerorhachis* strongly restricts bending even in a long axial column section, so its low head mobility is only partly due to its very short neck (Supplementary Table 9).

Crownward tetrapods tend to have a relatively wide axial column, as seen in *Greererpeton* (Extended Data Fig. 2e). This is due in part to robust transverse processes^33^—a geometry that is favourable for greater mobility (Supplementary Table 9). In other taxa, such as embolomeres and amniotes, this widening is also correlated with an increasing replacement of the notochord by vertebral centra. Our current method is inappropriate for estimating the range of mobility in taxa such as these, with restricted notochords. The zygapophyseal connections between vertebrae are also elaborated in crown tetrapods, adding complex joints to the vertebral column. Evaluating early tetrapod and early amniote axial columns with more developed zygapophyseal articulations will require a different approach.

### Further constraints on neck mobility

Our estimates of lateral flexion, extension and rotational capacities are based on the flexural and torsional rigidity of the axial column. In a whole body, additional tissues superficial to the axial column—namely the integument, muscles, osteoderms and ribs—also contribute considerable stiffness^48^. Further constraints on tetrapod neck mobility could also arise from internal organs that cannot tolerate the extension or compression caused by head motion, such as the gill apparatus in, for example, *Eusthenopteron*^40^, *Tiktaalik*^49^ and *Acanthostega*^50^.

### The axial column and terrestrialization

Our models of neck mobility across stem and early tetrapods contradict the predicted increase in neck mobility driven by head–shoulder separation^4,7–9^. Instead, they suggests that the separation event (which occurred among the common ancestors shared by *Tiktaalik* and *Acanthostega* but was not shared with *Eusthenopteron*^9,49^) had no detectable effect on neck mobility (Fig. 2). Overall, stem tetrapods had stiffer necks than might be expected given their soft tissue-dominated axial columns (the properties assessed are summarized in Supplementary Table 3). In these taxa, the capacities for lateral flexion and extension were limited to only a few degrees. Furthermore, the capacity for head rotation was absent or, at best, extremely limited in stem and early tetrapods. In exceptional cases among stem tetrapods (such as *Whatcheeria*) and derived crown tetrapods, the neck enables substantial and independent head motion; however, this ability seems to have evolved secondarily to the origin of the tetrapod neck (that is, head–shoulder separation).

Our results offer an alternative to the prevailing interpretation^4,5,7–9,51^ that the separation of the skull and pectoral girdle indicates independent neck mobility through a free neck in stem tetrapods. A stiff axial column, as inferred from our analysis, is consistent with the amphibious lifestyle hypothesized for most stem tetrapods^4,7,8,51^, in which low neck mobility may have been advantageous for locomotion in water by increasing energetic efficiency during swimming^52^. A stiff axial column may also have contributed to passive resistance to gravity in terrestrial environments. Thus, given the limited neck mobility in stem tetrapods, alternative functional explanations for the head–shoulder separation are warranted.

We argue that the head–shoulder separation is more consistent with the predicted origin of forelimb-driven locomotion in early tetrapods^4,11–14^. The pectoral fin and girdle underwent several anatomical changes across the tetrapod stem^8,12^, including the rearrangement of the long bones and the loss of lepidotrichia^13,52–54^. *Eusthenopteron*, the earliest-branching tetrapodomorph considered, already has a stress-dissipating trabecular architecture in its humerus that could support weight bearing^14^. More derived stem tetrapods such as *Ichthyostega* are hypothesized to have used pectoral-powered locomotion in contact with the substrate^11,42,51,55,56^, similar to the terrestrial locomotion observed in several extant fishes^13,57,58^. These lines of inference reveal a trend of increasing demand on the shoulder girdle to support forelimb function^4,9,11,27,58^ rather than to connect the head with the trunk^5,6,15^ across the tetrapod stem. Thus, the head–shoulder separation in stem tetrapods probably resulted from this shift in primary shoulder function.

In fact, the tetrapod neck seems to have evolved as a by-product of the head–shoulder separation, rather than as an adaptation to increase neck mobility. Although the axial column remained generally stiff in stem tetrapods, derived tetrapods acquired greater neck mobility, partly by having longer necks and partly through the evolution of mobile intervertebral joints^59^ that enable lateral flexion, extension^60^ and rotation^61^. The delayed acquisition of neck mobility calls into question the relevance of some hypothesized terrestrial feeding modes for early tetrapods^51^, because such limited head mobility would preclude lateral snapping when the forelimb was planted except, perhaps, in *Whatcheeria*^42^. Meanwhile, our results align with hypotheses regarding aquatic feeding behaviour that suggest that additional lateral flexion in the trunk contributes to overall motion^51,62^ and broadly support amphibious locomotory habits among stem and early tetrapods^11,42,51,56^.

## Methods

### Materials

Ten *A. fulvescens* yearlings were partially dissected for material characterization. This number was not predetermined statistically. The fish were housed and cared for prior to being provided to us dead in accordance with the animal use protocol (22003) approved by the Canadian Council on Animal Care and Wilfrid Laurier University’s Animal Care Committee. Additional specimens from the same source, as well as waste from commercially fished sturgeon, were used for test design and figures (Extended Data Table 3).

### Material testing

The tested specimens were thawed and their axial columns were dissected out in the morning on the day of testing (Extended Data Fig. 1a, Supplementary Methods, Supplementary Table 1 and Supplementary Figs. 1 and 2). Each dissected axial column included a cube of bone and cartilage from the posterior-most section of the skull and the caudal fin to avoid cutting the notochord at any point along its length and compromising its mechanical properties. Dissected specimens were kept moist and stored on ice between dissection and testing and between trials. The column was dissected further on site for torsion testing of specific sections and for testing the notochord (Supplementary Methods). Tissue bending was measured on a ZwickiLine Z5.0 TN materials testing machine (ZwickRoell) using a ZwickRoell GTM series K load cell (5 kN capacity, International Organization for Standardization (ISO) 7500-1 accuracy class 1 from 20 N to 5 kN). Measurement electronics provide a 24 bit resolution across the range corresponding to a force resolution finer than 0.01 N; although our maximum loads fell below the threshold of the ISO, they remained well within the electronic resolution of the system and were repeatable across measurements. Torque was measured using a 20 Nm torque transducer (ISO 7500-1 accuracy class 1 from 0.2 Nm to 20 Nm) on the same machine. The full-scale range corresponds to a resolution finer than 0.0001 Nm; although some of our measured torques fell below the ISO threshold, they also remained well within the electronic resolution of the system. The first round of manipulation in each trial is the source of data in this study, with additional rounds being performed in case these data are of interest for future materials assessments.

#### Whole-column bending

First, three-point lateral bending (equivalent to lateral flexion) was performed with the supporting pins set at a 4 cm span and the loading pin set to a 5 mm depression depth (five rounds of depression per trial). Next, dorsoventral bending (equivalent to extension) was conducted with the same span but with the depression depth decreased to 3 mm to avoid bending the tissue to a damaging degree.

#### Whole-column torsion

After the bending tests, the column was dissected further into sections between every 12th rib and the subsequent rib. The skull cube was not removed to avoid cutting the anterior end of the notochord. Each section was clamped vertically in the machine with the anterior part facing upwards. The torsion drive was then rotated 30° clockwise, held for 1 s and returned to the starting position three times per trial.

#### Notochord bending

The notochord was dissected from each column. Lateral bending and dorsoventral bending were performed consequently with the supporting pins set at a 2 cm span and the loading pin set to a 3 mm depression depth (five rounds per trial). Small pieces of coarse sandpaper (60–100 grit) were placed between the sample and support points to stabilize the specimen when it rolled out of the desired orientation during set-up. We ensured that the added sandpaper did not overlap with the span between the supporting pins so as to avoid increasing the stiffness of the notochord area that was being tested.

#### Notochord torsion

Testing trials for specimens 2401-01 and 2401-02 followed the same protocol as for whole-column torsion measurements. During initial trials, we observed a sinusoidal curve indicative of viscoelastic properties. We therefore increased the hold time to 3 s at 30° for specimens 2401-04–2401-10.

### Measurements

#### Sturgeon

Total column length (from skull cube to tail tip), torsion gauge length and notochord diameter after dissection were measured on the day of testing. The notochord diameter at the midpoint of each section was used during data processing. All other measurements were taken from photos in ImageJ2 (v.1.42q)^65^.

#### Stem and early tetrapods

Measurements of *T. roseae* were taken using Blender^66^ from a computed tomography reconstruction of the axial column of NUFV 108 by T.A.S. Measurements of *A. gunnari*, *G. burkemorani* and *T. insignis* were taken using Fiji^67^ from two-dimensional reconstructions modified by B.K.A.O. from their source publications. Measurements of *W. deltae* were taken using Fiji^67^ from two-dimensional reconstructions by B.K.A.O. based on direct observations (see Supplementary Fig. 7 caption and Supplementary Table 12 for references).

### Data processing

Raw data were processed in R^68^ and Rstudio^69^ using the CrshR repository^70^. The CrshR functions are used to analyse each bending or torsion cycle. For bending, the functions produce the maximum bending load (N), maximum bending displacement (mm), maximum bending angle (degrees) and work done to achieve maximum displacement (Nm). For torsion, the functions produce the maximum torque (Nm) and the angle at maximum torque (degrees). These data were further processed and plotted in R using functions in the Korneisel_TetNecks_2026 repository^71^ and the ggplot2, ggthemes, readr, dplyr and RColorBrewer packages^72–78^. Equations were published previously^79^; any modifications are indicated.

For bending trials, we first calculated the flexural rigidity (*EI*, Nm^2^) using:

1 $${EI}=\frac{{{FL}}^{3}}{48d}$$

where *F* is the maximum force (N), *L* is the three-point bending rig span (m) and *d* is the maximum displacement (m). We next used the following known equations for the second moment of area (*I*) to calculate Young’s modulus (*E*). For the notochord, we used:

2 $${I}_{\mathrm{circle}}=\frac{{\rm{\pi }}{r}^{4}}{4}$$

where *r* is the radius (m). For the vertebral column bent laterally, we used:

3 $${I}_{{\rm{triLat}}}=\frac{h{b}^{3}}{48}$$

and for the vertebral column bent dorsoventrally, we used:

4 $${I}_{{\rm{triDV}}}=\frac{{h}^{3}b}{36}$$

where *h* is the height of the estimated vertebral triangle (m) and *b* is the base of the estimated vertebral triangle (m).

For torsion trials, we first calculated torsional rigidity (*GJ*, Nm^2^ per radian) using:

5 $${GJ}=\frac{{TL}}{\theta }$$

where *T* is the maximum torque (Nm), *L* is the gauge length (m) and *θ* is the angle at maximum torque (degrees). We next used the following known equations for the polar moment of area or torsional constant (*J*) to calculate the modulus of rigidity (*G*). For the notochord, we used:

6 $${J}_{\mathrm{circle}}=\frac{{\rm{\pi }}{D}^{4}}{32}$$

where *D* is the diameter of the notochord (m). For the vertebral column, we used:

7 $${J}_{{\rm{t}}{\rm{r}}{\rm{i}}}=\frac{hb}{144}({4b}^{2}+{3h}^{2}).$$

The Young’s modulus was calculated from the three-point bending data for each matching position or section between specimens under both dissection conditions, and the torsion constant was calculated from the torsion data for each section and under both dissection conditions, using the CrshR package in R^68–70^.

### Modelling of extinct taxa

The Young’s modulus and the torsion constant for the axial column were determined from the measurements taken from the anterior-most axial column of *A. fulvescens* (Supplementary Table 1, bending position 1 (bending) and section number 1 (rotation)). The anterior position for whole-column bending and the first section for all other tests were posterior to the midline skull and fused occipital vertebrae but overlapped with the opercula in life, making them most comparable to the area of interest in stem tetrapods. The material values were then used to calculate lateral flexion, extension and rotation, along with *I* and *J* values obtained from the measurements of the neck region of each taxon (Extended Data Table 4 and Extended Data Fig. 2). These bending calculations revealed the force and the torsion calculations the torque needed to produce the deflections in the anterior axial column.

#### Lateral flexion estimation

For extinct taxa, the second moment of area for the anterior axial column was calculated using equation (3), and for the notochord using equation (2), on the basis of the measurements shown in Extended Data Table 4.

We used equation (8), which gives the deflection of a beam under cantilevered bending solved for force, to calculate the force required to achieve a specific lateral deflection. Data were collected using three-point bending rather than cantilevered bending. This is because the three-point set-up enables the paired supports to be placed closely, so that the soft tested material can start in a neutral position, rather than a bent one because of the force of gravity. Cantilevered bending, however, is a good approximation for the bowstring model of axial bending and was therefore used to model the estimated lateral flexion and extension.

8 $$F=\frac{\delta 3{EI}}{{L}^{3}}$$

In equation (8), *δ* is deflection, *E* is the calculated Young’s modulus for position 1 (whole-column bending) or section 1 (all other measurements) of *A. fulvescens*, *I* is the second moment of area of the relevant axial-column section of the taxon of interest and *L* is the length of the cervical region or its equivalent.

For each of these equations, deflection was treated as the opposite end of a right triangle with the adjacent side length of *L* to determine the angle of deflection (equation (9)) using the ATAN function in Excel. This deflection was obtained in radians and converted to degrees using equation (10).

9 $${\tan }^{-1}\left(\frac{\delta }{L}\right)$$

10 $${\delta }_{\mathrm{deg}}=\frac{{\delta }_{{rad}}\times 180}{\pi }$$

Striated muscle in vertebrates is known to generate pressures of 200–300 kPa (kilonewtons per square meter of muscle in cross-section)^79^. Using this known range, we calculated the muscle cross-sectional area required to achieve these forces. These muscle areas were capped for each taxon at half the area of an oval fitted around the posterior skull. This was chosen to provide the absolute maximum muscle area available to perform lateral flexion (area on one side) and extension (dorsal area of both sides combined; Supplementary Fig. 3). The angle of deflection produced by the maximum force from the corresponding muscle area in each species represents the estimated maximum range of motion for the animal.

#### Extension estimation

The second moment of area for the anterior axial column was calculated using equation (4). All other steps followed the same process as that for lateral flexion.

#### Rotation estimation

The polar moments of area for torsion on the column were calculated using equation (6) and for the notochord using equation (7). *G* was averaged from the sturgeon vertebral column, as it did not change significantly with position along the column. *J* was calculated from measurements of extinct taxa. We calculated torque (*T*) at small angles (5–10°) using equation (11), which is equation (5) solved for *T*:

11 $$T=\frac{G\,J\,\theta }{L}.$$

For rotation, we started with small angles and found that the column required very high torques to achieve any rotation (Extended Data Table 1).

### Validation

The method for estimating range of motion was validated by applying it to lungfish, an extant taxon, described in a previous study^80^, in which elevation of the cranium was measured in vivo at a maximum of about 5° during feeding strikes over an axial column length of 5 cm. We measured the axial column and body cross-sectional area in computed tomography scans of the original specimens using Materialise Mimics (v.26.0). Our estimate of the maximum extension along this axial column length was 5.25° or 8.75°, depending on the proportions of each individual.

### Reporting summary

Further information on research design is available in the Nature Portfolio Reporting Summary linked to this article.

## Online content

Any methods, additional references, Nature Portfolio reporting summaries, source data, extended data, supplementary information, acknowledgements, peer review information; details of author contributions and competing interests; and statements of data and code availability are available at https://doi.org/10.1038/s41586-026-11158-3.

## Supplementary information

Supplementary Information Supplementary Materials and Methods, including Supplementary Figs. 1–4, Supplementary Tables 1–9 and Supplementary References.

Reporting Summary

Supplementary Data A multi-sheet Excel file containing measurements of extinct taxa, equations for estimating mobility and instructions on what information to input for anyone interested in producing an estimate for another taxon or body region of interest using our methods. This replaces the version from previous revision rounds in the GitHub repository.

Peer Review File

## Source data

Source Data Extended Data Figs. 3 and 4

Source Data Extended Data Fig. 5

## Figures and Tables

**Extended Data Table 1:**
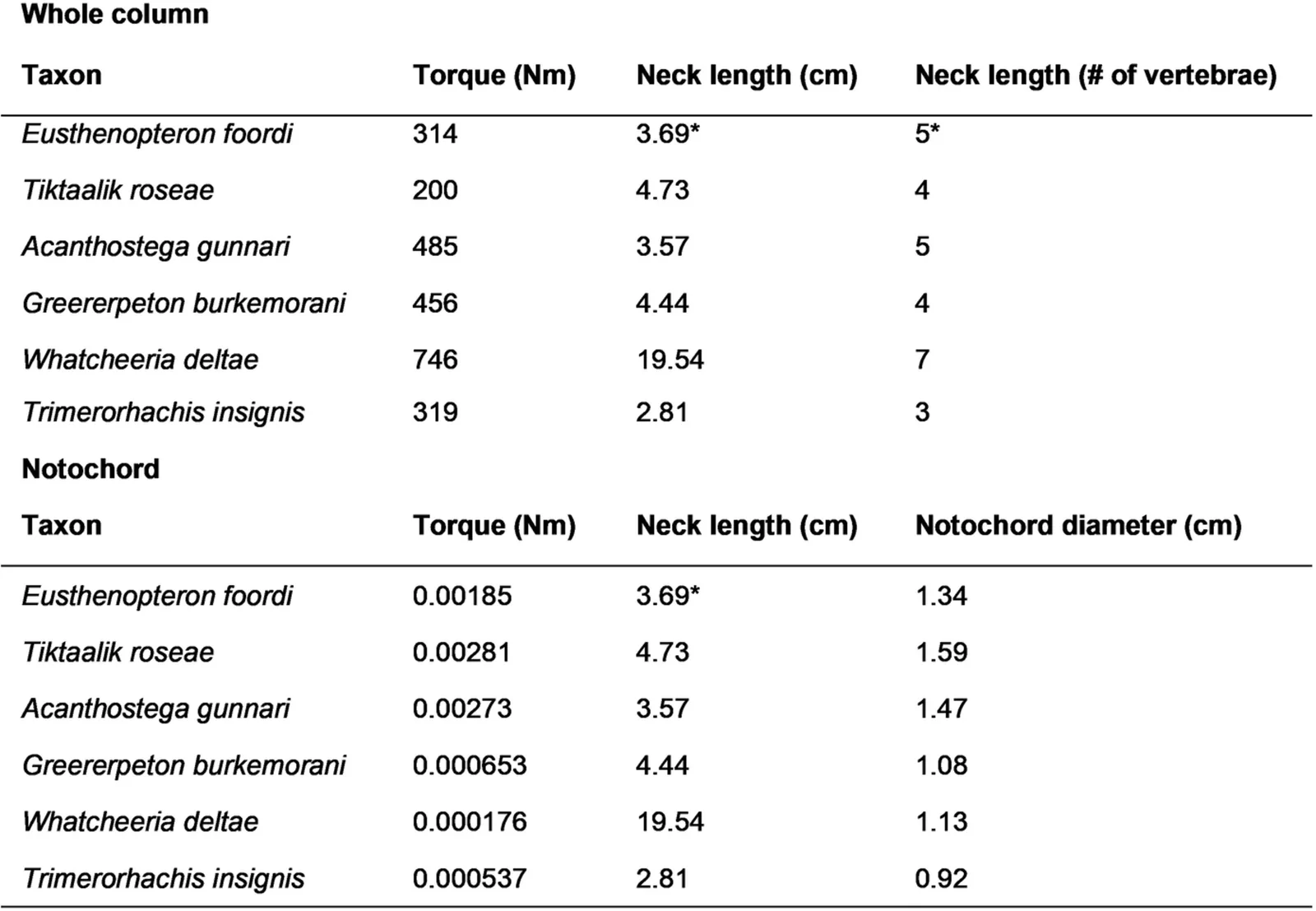
Torque necessary for 5 degrees of head rotation in extinct taxa

High torques would be necessary to twist the anterior axial column for even small head rotations, but the notochord alone is much less resistant to torsion. *E. foordi does not have a cervical region. The first 5 vertebrae are anatomically distinct^30^, so their length was used here, but they are overlapped by the opercular bones covering the gills.

**Extended Data Table 2:**
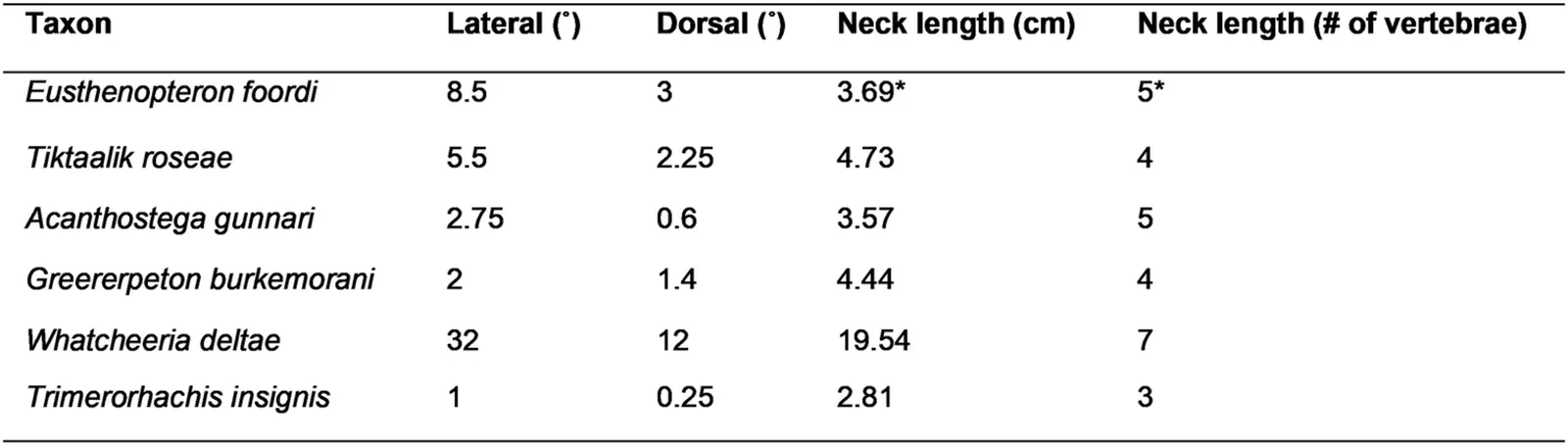
Maximum lateral flexion and extension estimates (degrees) for extinct taxa as limited by the axial column

Estimates, also imaged in Fig. 2, with original sources referenced for neck length corresponding to Extended Data Fig. 2, as follows: *Eusthenopteron*^30^, *Tiktaalik*^41^, *Acanthostega*^2^, *Greererpeton*^43^, *Whatcheeria*^42^, *Trimerorhachis*^44–47^. *First five vertebrae and length across their centra.

**Extended Data Table 3:**
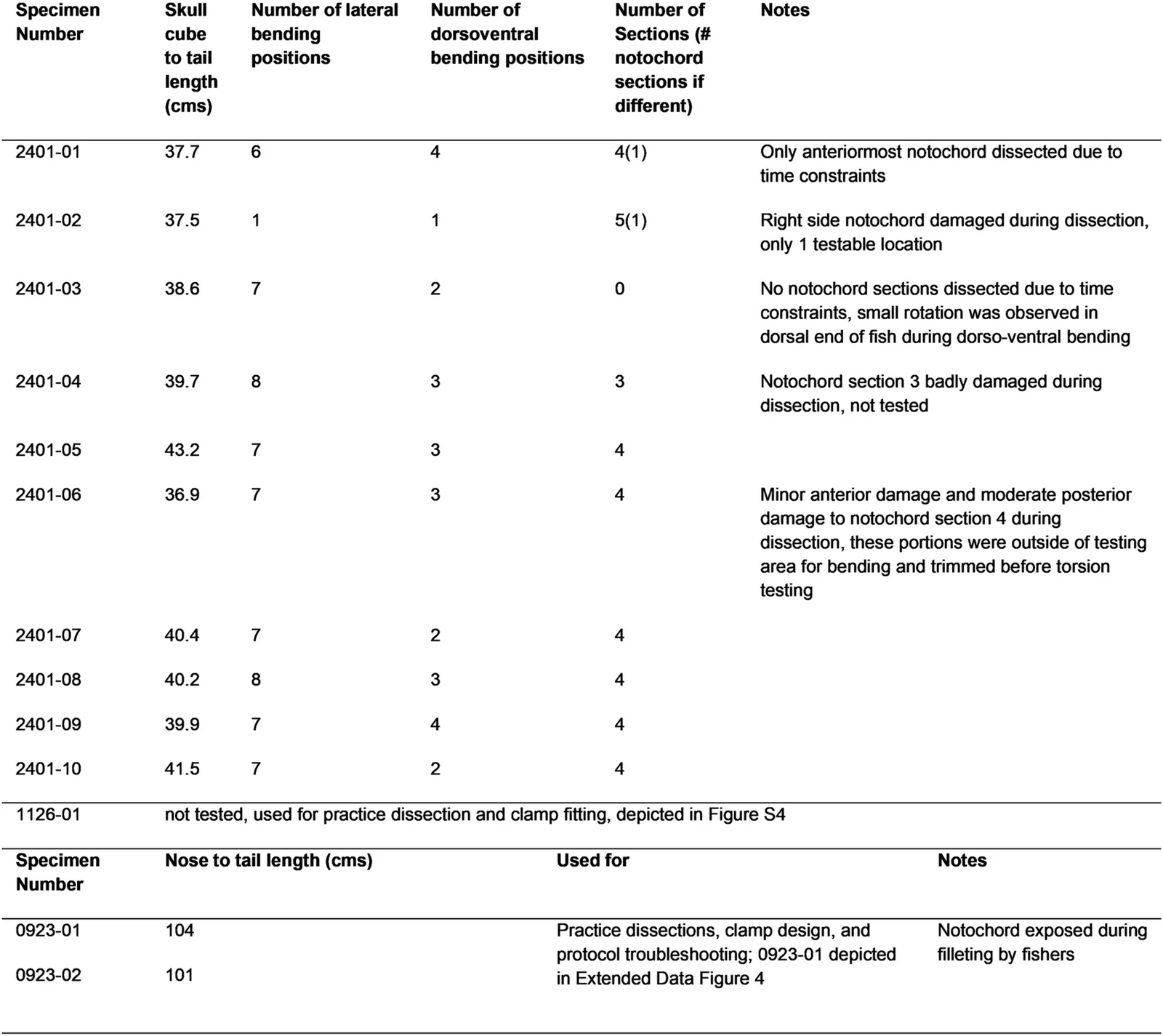
Specimens used for Materials Testing

*Acipenser fulvescens* specimens used for design and implementation of materials tests.

**Extended Data Table 4:**
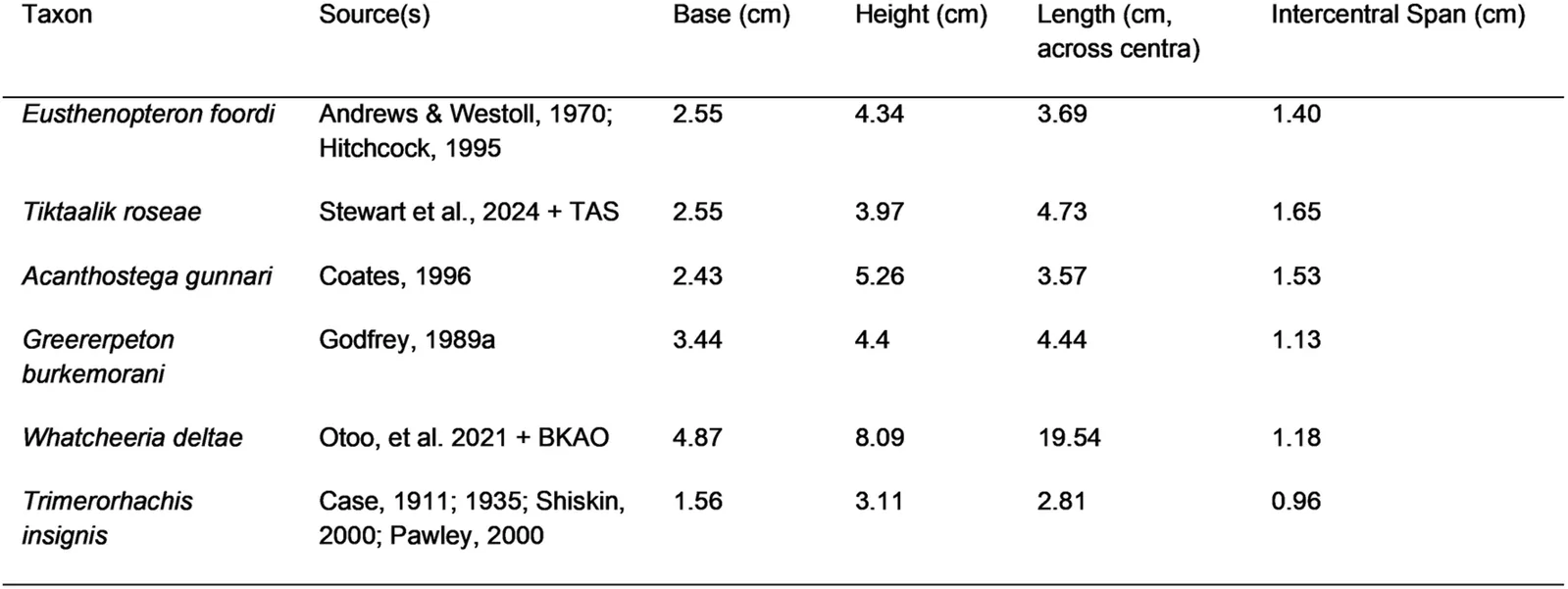
Area of interest measurements from extinct taxa

Sources and linear measurements necessary to perform deflection calculations to estimate mobility.

## Data Availability

The data necessary to replicate our results are available at GitHub^71^ (https://github.com/CDonatelli/Korneisel_TetNecks_2026) or at Figshare^81^ (https://figshare.com/articles/dataset/Korneisel_TetNecks_2026/33552199?file=68415544). Materials not available in this paper, its Supplementary Information or the repository are available from the corresponding author. Source data are provided with this paper.

## Code availability

The codes used to analyse universal testing machine output data are available at GitHub^70^ (https://github.com/CDonatelli/CrshR). Additional codes specific to the analysis of this method, as well as modifications to the base CrshR code, are available at GitHub^71^ (https://github.com/CDonatelli/Korneisel_TetNecks_2026) or at Figshare^81^ (https://figshare.com/articles/dataset/Korneisel_TetNecks_2026/33552199?file=68415544).
