# Supplementary material for: Early tetrapod neck mobility modelled using an extant analogue
Source: Nature. 2026 Oct 14;659(8139):128–32. doi: 10.1038/s41586-026-11158-3 (PMC13736659; doi:10.1038/s41586-026-11158-3)
Supplement: Supplementary file 1 [file 41586_2026_11158_MOESM1_ESM.pdf]

nature portfolio

https://doi.org/10.1038/s41586-026-11158-3

Supplementary information

Early tetrapod neck mobility modelled using an extant analogue

In the format provided by the authors and unedited

Korneisel *et al.*, 2026 "Early tetrapod head mobility" Supplemental Information

Supplemental Materials & Methods

Materials

Axial columns were harvested from adults of *Acipenser fulvescens* provided by Péché Fumé. These specimens were used to develop protocols for materials testing, and one of these (specimen #1126-01) was used for figures (Table S3; Fig. S9).

The fish originated from a cohort of eggs collected from the wild by members of the Rainy River First Nations aquaculture conservation team and raised in captivity at the Ontario Aquacultural Research Center. The fish were then transferred to and raised further by the Wilkie lab at Wilfrid Laurier University.

Specimen preparation / storage

Individuals 2401-01 through 2401-10 were euthanized with MS-222 and gutted through a ventral midline incision and their brain, heart, and a lateral skin/muscle sample was removed. The skull anterior to the pectoral girdle was removed along with the brain at this time (Fig. S1A). The fish were frozen on the same day as euthanasia by placement in a -20° freezer after dissection and then mailed by overnight courier to Carleton University in insulated containers on ice. They were inspected on arrival and found to have remained fully frozen. They were then stored in a -20° Celsius freezer until preparation for biomechanical testing. A single freeze-thaw cycle and freezing at -20° Celsius was chosen based on literature indicating that it would have a minor effect on the biomechanical properties of the tissues (Changoor *et al.*, 2010; Kaye *et al.* 2012; Blaker *et al.*, 2024; Dhiman *et al.*, 2025) and based on observations of sturgeon tissues by DEK across multiple freeze thaw cycles during experimental design indicating that tissues degraded with added freeze-thaw cycles.

It was determined that the specimens needed over 24 hours to fully thaw to be further dissected for biomechanical testing. Each specimen was removed from the freezer and thawed in a 4° Celsius refrigerator for 36 hours prior to testing. After 16–18 hours, when minimal ice remained, the bulk dissection removing the osteoderms, pectoral fins, excess skull material, and lateral musculature from the vertebral column was completed. If sufficiently thawed to allow precision, the precise removal of muscle tissue from around the axial column and trimming of ribs to stubs next to the axial column was completed at this time. If not, the specimens were allowed to continue to thaw overnight and cleaned in the morning within 2 hours of testing.

Bulk dissection

A cube was trimmed out of the remaining portion of the posterior skull of each specimen anterior to the notochord where bone shears were used to trim down the dermal skull bones and remove the pectoral fins. Next, surgical scissors were used to cut the skin and superficial musculature between the dorsal and adjacent lateral osteoderms (Fig. S1B). Surgical shears were then used to cut parallel to the axial column down to the end of the lateral osteoderms on the sides of the tail then around the posterior-most osteoderm (Fig. S1C). The ventral row of osteoderms posterior to

the pelvic fins and surrounding ventral skin was then removed by cutting anteriorly through the ventral caudal fin with surgical shears, ventral to the position at which the notochord curves dorsally into the upper caudal fin lobe (Fig. S1D, lower). This cut was propagated with a scalpel along the ventral edge of the axial column to separate the anal fin and remaining ventral skin (Fig. S1D, upper & Fig. S1E). The initial longitudinal cuts were then deepened to the lateral ribs from the dorsal cuts (Fig. S1D, upper and middle) and the lateral ribs were cut near the column from inside the body cavity to meet the previous cut from above and remove the lateral tissue (Fig. S1D, lower). The lateral cuts met the cuts around the posterior-ventral part of the fish to remove the lateral musculature, ventral skin, and pelvic fins. This left the axial column in a block of muscle along with the skull cube and the dorsal osteoderm row (Fig. S2A). Surgical shears were used to then cut the skin between the dermal skull bone and the anteriormost dorsal osteoderm that is not ossified to the dermal skull (Fig. S2B). A scalpel was used to cut under the dorsal skin and row of osteoderms along the whole column from each side until the cuts met medially and the dorsal osteoderm row was separated from the axial column, removing also the dorsal fin (Fig. S2A). In most specimens, the remaining column was still frozen at the time of bulk dissection and precision cleaning happened the following day. Axial columns that were still frozen were wrapped in dampened paper towel and stored in the 4°C fridge for precision dissection the following day (i.e., day of testing).

Precision dissection / cleaning

Once the axial column was isolated and thawed, the fatty tissue underlying the dorsal osteoderms was trimmed along the dorsal portion of the column using surgical shears until the dorsal peak of the neural arch was palpable near the surface. Surgical shears were also used to remove lateral muscle from the posterior third of the axial column and the anterior two thirds only in cases where the ribs were not yet exposed. Then, a shallow ventrolateral cut was made along the length of the axial column to either side of the neural arches using a scalpel. Next, a similar shallow dorsomedially oriented cut just dorsal to the ribs was made. Each of these was propagated in shallow cuts, following the profile of the cartilaginous arches, until the cuts met, and the muscle was removed from the column. When necessary, the ribs were then trimmed to nubs to improve countability and fit for torsion clamping.

Clean columns were wrapped in dampened paper towel and placed in a plastic bag to be stored in the 4°C fridge (when specimens were sufficiently thawed so that precision dissection occurred on the same day as bulk dissection) or on ice until testing (when precision dissection occurred on the same day as testing).

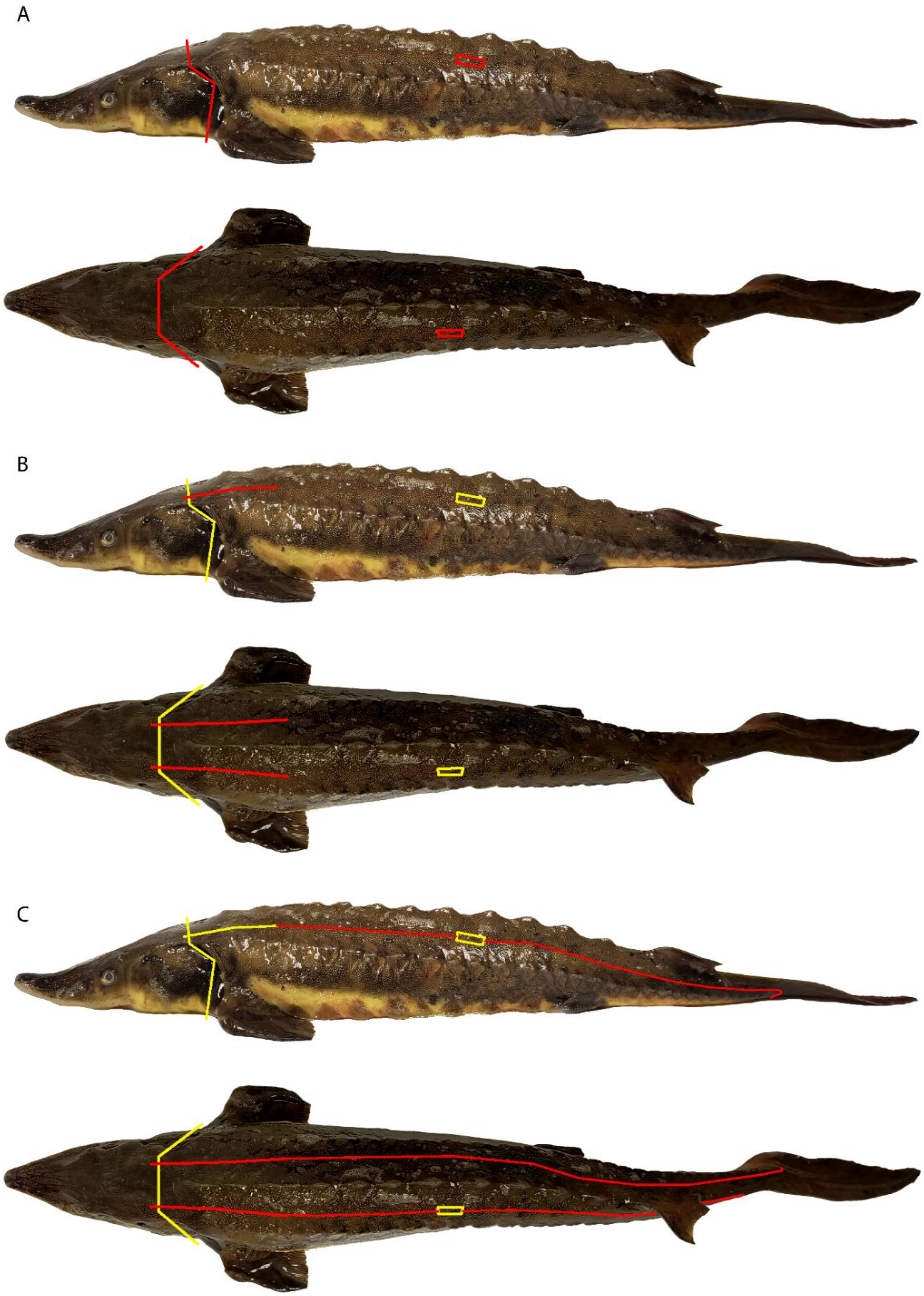

Supplemental Figure 1. Dissection process illustrated on a whole *A. fulvescens* specimen (specimen #1126-01).

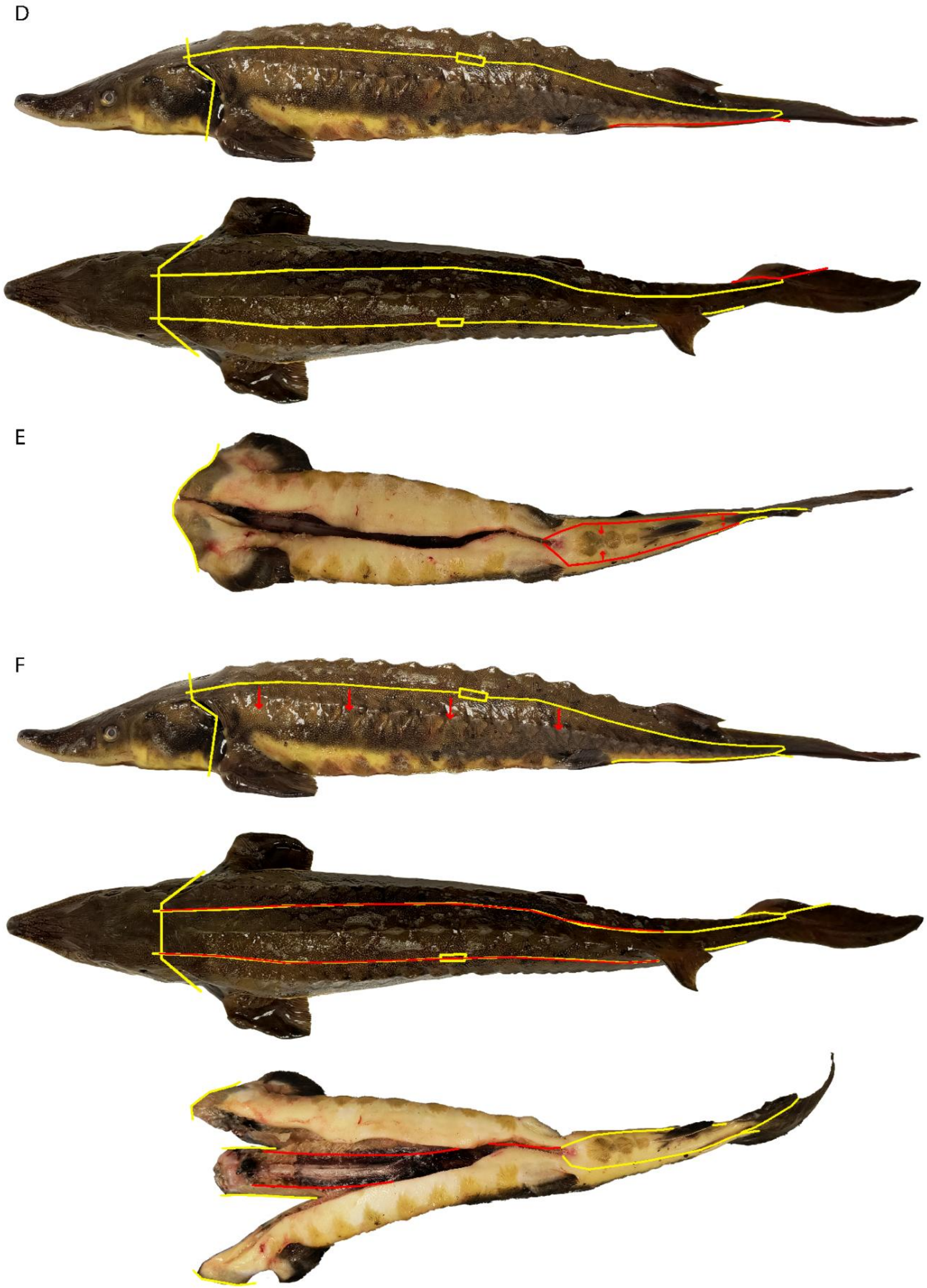

Supplemental Figure 1 cont. Dissection process illustrated on a whole *A. fulvescens* specimen (specimen #1126-01).

Supplemental Figure 1 cont. Dissection process illustrated on a whole *A. fulvescens* specimen (specimen #1126-01) in order of operation. **A.** Cuts made before freezing and shipping. **B.** First set of cuts made after thawing with bone shears. **C.** Cuts from B propagated posteriorly with surgical scissors. **D.** Ventral caudal fin cut and initial cuts made with shears. **E.** Removal of ventral osteoderms, skin, and fin with scalpel. **F.** Lateral muscle, lateral osteoderms, and ventral osteoderms and skin removed by propagating cuts ventrally and cutting parallel to the axial column from inside the body cavity. For each panel top = left lateral view, bottom = dorsal view. Red lines indicate location of new cuts, yellow lines indicate locations of previous cuts.

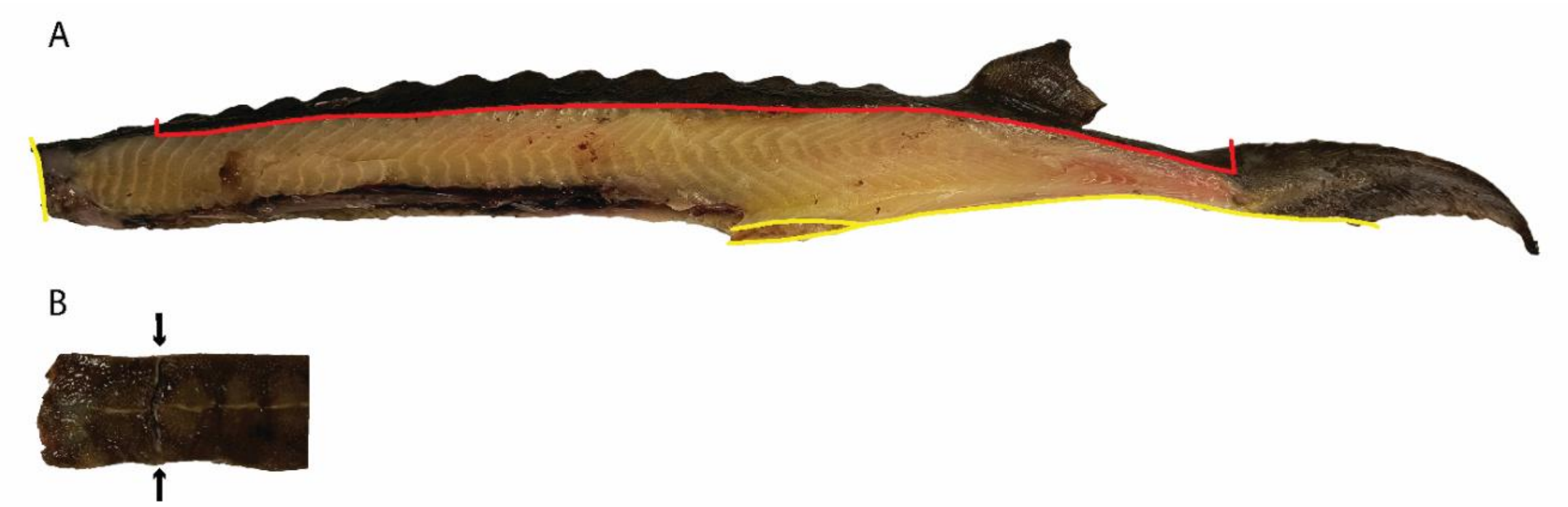

Supplemental Figure 2. Final steps of bulk dissection process illustrated on partially dissected *A. fulvescens* specimen (specimen #1126-07). **A.** *A. fulvescens* axial column in left lateral view, dorsal osteoderms removed by cutting ventral to the skin. **B.** the location of the cut separating the first free dorsal osteoderm from the skull cube indicated by black arrows. Red lines indicate location of new cuts, yellow lines indicate locations of previous cuts.

Measurements

Each whole axial column specimen was photographed with scale before starting testing and the full length from anterior skull cube to tail tip was measured and recorded. Each column was photographed directly after sectioning for whole column torsion with each section separated slightly, and each section and every cut face was photographed with a scale bar for record and later calculation of cross-sectional area. For whole column torsion testing, a picture with scale bar was taken of each “Section 1” to measure the span minus the length of the specimen stabilized within the skull cube.

Measurements to determine an idealized triangular prism shape for the column were taken in Fiji (Schindelin *et al.*, 2012) from the images captured of cross-sectional cuts just before torsion testing of the whole column. For each section, the posteriormost measurements were applied during data processing. As both sides of each cut were imaged, each measurement was taken both from the posterior face of the section and the anterior face of the next section. These two measurements were averaged. Height (h) was measured from the dorsal tip of the neural arch to the ventral midline point of the basiventral just below the notochord, excluding empty space

for the hemal canal. Width (b, for base) was measured at the ventral-most point of the height measurement as shown in Figure S6. Calibration was done on a one-centimeter span of the scale bar captured in each image.

For the lungfish validation step, measurements of axial column Base width and body cross sectional area were taken directly on a 3D rendering of the vertebrae and a rendering of all mineralized tissues including the scales that denoted the body outline in Materialise Mimics version 26.0. A screencapture was taken in cross sectional view with the Base measurement marked and the height measurement was determined from measuring an idealized triangle shape in Adobe Photoshop calibrated to the base width.

For intercentral span measurements in *T. roseae*, the ruler ends were fixed on the interior face of the intercentrum. This line was placed at the maximum intercentral width in anterior view then checked to be perpendicular to the long body axis in dorsal view. The space between intercentra of each vertebra that preserved both intercentra was measured. The resolution of the scan from which this reconstruction was segmented is 122.441 um

The length of the ossified portion of *Tiktaalik*'s axial column was taken from the anterior midline edge of the first left-side intercentrum to the posterior midline edge of the last left-side intercentrum. The last few partial vertebrae preserved posterior to the column were excluded from this measurement. The length of the anterior portion of the axial column lacking ossifications was measured between the posterior point on the left exoccipital and the anterior point of first intercentrum used in the previous measurement. An anterior view screenshot of each vertebra with both of its intercentra preserved was taken in Blender (Community B.O., 2018). These images were calibrated based on the known intercentral span (method of measurement in the previous paragraph) in Fiji (Schindelin *et al.*, 2012). The neural arch was moved dorsally to minimize overlap with the notochord while recognizing likely crushing of the neural arch pedicles together (Fig. S6 C *vs* E) then height (h) and width (b) were measured to determine the appropriate size for the column idealized to a triangular prism shape.

Measurements from all other taxa (Extended Data Table 5) were also taken from the anteriormost point of the first intercentrum to the posterior edge of the last cervical (or non-cervical anterior column region, as in *Eusthenopteron*) vertebra centrum, measured on reconstructions to the nearest tenth of a millimeter (Extended Data Figure 7, note that the reconstruction of *Whatcheeria* is modified from the original as described in the figure caption). Except for in *Eusthenopteron*, the definition of the cervical vertebral region followed that of the source material (citations in Extended Data Figure 7)

Testing

Before testing, 1123-05 was dissected from a fresh non-frozen condition approximately following the protocol described above to determine an effective dissection protocol for tested specimens. It was then used to determine the spacing for the stable points during bending tests. The axial column and then notochord sections of 1126-01 were placed across two dissection pans with a gap between. These were adjusted to various spans to determine a testing span at which no droop occurred between supports for each dissection condition and testing direction. Mock

depressions were performed at the midpoint by hand to determine the depth of bending that could be accomplished without turning or toppling the specimen.

Lateral Bending – Specimens were placed with their right side up and anterior to the left on aluminum stable points spaced at 4 cm mounted to the load cell platform with spring clamps. A small plywood support table nested inside and a larger support table at the same level as the stable points were mounted around the apparatus to stop the fish from bending over the stable points under their own weight.

The descending point was made of firm plastic and was lowered to just above the specimen before each run. The middle of the descending point was placed at position one, between the 11th and 12th rib stub. The depth of depression was 5 mm and the test was done with five repetitions. Position two was between the 17th and 18th rib stub, position three six more ribs posterior (between the 23th and 24th) and the same for position four. Starting at positions five, six, or seven depending on the rib palpability in each individual, each position was 3 cm successively posterior to the previous position (this was always the case for positions seven and eight). If ribs were palpable, then the criterion of six ribs more posterior for the next position was kept. Each specimen was tested posteriorly until the last position at which placement no tail fin or undissected tail skin would be within the testing span. The base of the tail sat on the right-side stable point for position seven of #2401-06.

Dorso-ventral bending – Next, dorso-ventral three-point bending testing was conducted with the anterior of the specimen to the left. The depth of depression was decreased to 3 mm, but the remainder of the testing protocol was the same including positions (projected dorsally from the ribs, this frequently placed the descending point between two neural arches). The more posterior positions were not possible for dorsoventral bending due to the tail flopping to one side and twisting the posterior end of the specimen. Testing was only performed until the last position at which no twisted tissue was in or immediately adjacent to the testing area. The specimen was watched during each trial for any sign of rolling (i.e., instead of bending, the column started to fall over laterally). Trials in which rolling was observed or the specimen fell over were discarded.

Torsion testing – Torsion testing of the whole column was performed next. Posterior to palpable ribs, the specimen was sectioned at lengths approximately equal to the previously cut sections. The column with each section separated slightly and every cut face was photographed with a scale bar for record and later calculation of cross-sectional area.

“Section 1” was affixed to a square clamp with four bolts so that the brain cavity was visible through the attachment point to the machine and the column protruded such that it would be positioned vertically when mounted on the machine with the dorsal side facing in. It was then mounted on the machine and lowered into the open triangular clamp to receive the posterior end then secured with three bolts. The testing span was measured between the middle of the top and bottom bolts as this is where the specimen was held inside each clamp. The top clamp was turned 30 degrees to the right then returned to the start position three times per trial.

The specimen was watched during each trial for any sign of movement in the clamp.

Notochord bending – After completion of whole column torsion testing, the notochord was dissected from each column section starting with the first section. In this section, the

basioccipital cartilage was at least trimmed to the diameter of the anterior notochord or removed completely when possible. In the anterior section, the first step was to remove any remaining basisphenoid bone, then cut the chondrocranium into a wide wedge from between the cranial ribs to the anterior midline. The dermal bone was then cut off and the chondrocranium was shaved down to just dorsal of the notochord.

After this step, for sections one first and for all other sections, the neural arches were removed via scalpel and forceps, then the basiventrals were cut away from the notochord at each end with a scalpel and peeled from the notochord using forceps. The scalpel was used to separate any tissues that remain adhered to the notochord. Any remaining arch cartilage was peeled off with forceps until the notochord was isolated. The sections were stored in dampened paper towel and a plastic bag on ice prior to testing. Each trial was watched for any sign of rolling, in which the whole cylinder of notochord rotated laterally out of the intended orientation for the trial. If minimal rolling occurred, it was noted for each section and sand paper was added under the specimen to stabilize it as noted in the main methods. If non-minor rolling occurred, such as a dorsoventral trial rolling over into a lateral orientation, and this could not be stopped by the addition of sandpaper in a second trial attempt, then those trials were discarded from analysis.

Notochord Torsion – Each section of notochord was clamped with a pair of 4” nylon zip-ties with a loop tensile strength of 80N (Commercial Electric, model # GT-100M) that had been pre-bent around the handle of a blunt probe. The anterior end tie was fitted into a clamp that matched the tie’s clamped profile with a snug fit, then the specimen was lowered into the lower matching clamp until the specimen bent slightly (to avoid placing the tissue under tensile stress) and the posterior-end zip-tie was fitted snugly into the lower clamp. Each trial was watched for signs of motion of the specimen within the clamp or the zip-tie in the machine attachment.

Data Processing

As data output from the Universal Testing machine was force of stress by time during each trial, an additional column was produced in the rough data output files for each set of trials to match the testing protocol. For bending, this was a column of the distance of descent at each timepoint based on the total depression, hold time at the end and start positions, and rate of descent and rise. For torsion, the same information was generated except for degrees of rotation rather than distance of descent.

Supplemental Table 1. Schedule of dissection and testing. Initial testing run (on specimens #2401-01, 2401-02, and 2401-03) matches through start of column torsion, which lasted until about 2pm. Only two sections of notochord could be dissected due to time constraints that day.

**Time/Day**	**Action**
6-7 pm Sunday	3-4 specimens moved from -20°C to 4°C to thaw
7-8 am Monday	fish thawing progress checked
2 pm Monday	bulk dissection & precision dissection when possible
7 am Tuesday	precision dissection
7:30-9:30 am Tuesday	fish stored in damp paper towel on ice, transported to UOttawa
8:30-9:30 am Tuesday	whole column measured and photographed, column bending commences
c. 11:30 am Tuesday	columns sectioned and sections photographed, column torsion commences
	notochord dissections started on each section after torsion test is complete, stored on ice before dissection when necessary and after dissection until further testing
c. 12:30 pm Tuesday	Notochord bending commences
	when all notochord dissections are complete, notochord is clamped on each end with zip-ties in preparation for torsion, the section length is measured between the zip ties, and the notochord diameter is measured
c. 1:30 pm Tuesday	Notochord torsion commences
3-4 pm Tuesday	Testing complete

Supplemental Table 2. Definitions of testing positions and sections. The diagram below approximates these positions. Ribs are palpable for most of the anterior axial column after dissection, but measurements are used posteriorly where ribs are not palpable.

**Bending Position**	**Definition**	**Section Number**	**Definition**
1	Descending point placed between rib 11 & 12	1	Skull cube to rib 12
2	"" rib 17 & 18	2	Rib 13 to 24
3	"" rib 23 & 24		
4	"" rib 29&30, or in smaller fish 3 cm posterior to position 3	3	Rib 25 to 36
5	"" rib 35&36, or 3 cm posterior to position 4		
6	"" rib 41&42, or 3 cm posterior to position 5	4	Posterior to position 3, length matched to more anterior sections on a per specimen basis
7	3 cm posterior to position 6		
8	3 cm posterior to position 7	5	contains the tail, not tested

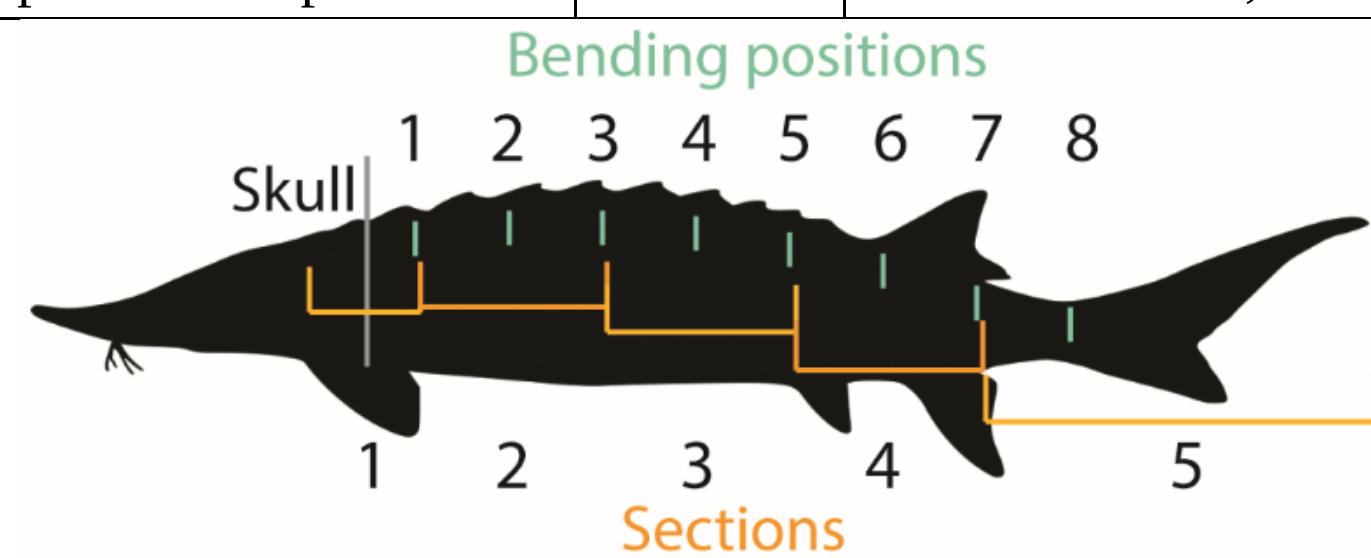

Supplemental Table 3: Notochord material data from notochord-dominated column testing in the literature

**Species**	**Part of the axial column**	**Type of test**	**Property Calculated**	**Value**	**Reference**
Myxine glutinosa	Notochord	Dynamic Bending	EI (Nm^{-2})	~0.2–0.3	Long *et al.*, 2002
Salmo salar	Notochord	Hydrostatic Pressure	EI (Nm^{-2})	$1E^{-5}$–$1E^{-12}$	Kryvi *et al.*, 2017
Xenopus laevis	Notochord	Bending	EI (Nm^{-2})	$1E^{-12}$–$1E^{-14}$	Adams *et al.*, 1990

Supplemental Table 4. Maximum lateral flexion and extension estimates (degrees) for extinct taxa as limited by notochord alone

Taxon	Lateral	Dorsal	Neck length (cm)	Notochord diameter (cm)
Eusthenopteron foordi	76.5	64	3.69*	1.400
Tiktaalik roseae	51	31	4.73	1.653
Acanthostega gunnari	44	25	3.574	1.526
Greererpeton burkemorani	81	72	4.439	1.128
Whatcheeria deltae	80	80	19.542	1.178
Trimerorhachis insignis	78.5	67.5	2.81	0.959

*Length of first five vertebrae across the centra

Supplemental Table 5. Statistics on mean, median, standard deviation, and number of trials for lateral bending trials. Maximum load and work during a trial were always observed in cycle 1.

**Lateral Bending**								
	**Dissection condition**	**Position**	**mean**	**median**	**Min**	**Max**	**sd**	**n**
Youngs Modulus (*E*, GPa)								
	column	Position 1	1.818988686	1.732622533	1327885248	3054046201	0.508605711	10
	column	Position 2	1.507190719	1.528807456	974082307	2344547966	0.430334545	9
	column	Position 3	1.730519254	1.457837646	549846919	4466335682	1.177081855	9
	column	Position 4	1.726376444	1.377994441	478091032	5223338928	1.441375972	9
	column	Position 5	1.523821705	1.440536379	897731555	2865709096	0.610412973	9
	column	Position 6	1.715452613	1.015701000	603735391	6765171727	1.923798297	9
	column	Position 7	2.824148902	2.263294113	1282931409	5017210796	0.149110817 0	7
	column	Position 8	2.207934486	2.207934486	1544046588	2871822383	0.938879269	2
	notochord	Position 1	0.524854242	0.299566058	216164385	1488517865	0.452186958	7
	notochord	Position 2	0.451667037	0.516318922	149475253	883750566	0.253313505	7
	notochord	Position 3	0.441672450	0.320595006			0.370568554	6
	notochord	Position 4	0.200692280	0.334967634			0.569261175	6
Flexural Rigidity (*EI*, Nm^2)								
	column	Position 1	0.568887334	0.607371497	0.290980482		0.15037728	10
	column	Position 2	0.472874082	0.530317736	0.27573533		0.11331449	9
	column	Position 3	0.391756463	0.374925804	0.285398936		0.08535781	9
	column	Position 4	0.367287657	0.323734212	0.281645942		0.10424921	9
	column	Position 5	0.242342961	0.22425549	0.168377721		0.06262634	9
	column	Position 6	0.198187252	0.177593172	0.123209012		0.06205992	9
	column	Position 7	0.23649199	0.27307756	0.108911085		0.06939161	7
	column	Position 8	0.169330227	0.169330227	0.12500732		0.06268206	2
	notochord	Position 1	0.037079503	0.015590999	0.006029934		0.06128613	7
	notochord	Position 2	0.021694549	0.016327897	0.006714133		0.01086296	7
	notochord	Position 3	0.013730456	0.009835732			0.01113662	6

	notochord	Position 4	0.006261675	0.006135578			0.01117245	6
Max Load (N) - Cycle 1								
	column	Position 1	1.42221833	1.51842874	0.72745121		0.3759432	10
	column	Position 2	1.18218521	1.32579434	0.68933833		0.28328622	9
	column	Position 3	0.97939116	0.93731451	0.71349734		0.21339451	9
	column	Position 4	0.91821914	0.80933553	0.70411485		0.26062303	9
	column	Position 5	0.6058574	0.56063873	0.4209443		0.15656586	9
	column	Position 6	0.49546813	0.44398293	0.30802253		0.1551498	9
	column	Position 7	0.59122998	0.6826939	0.27227771		0.17347902	7
	column	Position 8	0.42332557	0.42332557	0.3125183		0.15670514	2
	notochord	Position 1	0.18539752	0.07795499	0.03014967		0.30643066	7
	notochord	Position 2	0.10847274	0.08163948	0.03357067		0.05431478	7
	notochord	Position 3	0.06865228	0.04917866			0.05568308	6
	notochord	Position 4	0.03130837	0.03067789			0.05586223	6
Work (Nm) - Cycle 1								
	column	Position 1	3.03214606	3.081099742	1.8076861		0.7516279	10
	column	Position 2	2.40387841	2.67720743	1.2125985		0.7112607	9
	column	Position 3	2.01455055	1.974890461	1.3166683		0.4171681	9
	column	Position 4	1.77225883	1.754292511	1.219897		0.4870894	9
	column	Position 5	1.08567659	1.063085676	0.7003346		0.2891072	9
	column	Position 6	0.89682479	0.89394155	0.5531305		0.2904153	9
	column	Position 7	1.14773647	1.272661674	0.5461591		0.3128172	7
	column	Position 8	0.80843693	0.808436927	0.5881534		0.3115279	2
	notochord	Position 1	0.22171249	-0.04905083	-0.1396524		0.7088407	7
	notochord	Position 2	0.05796737	0.000119051	-0.2270783		0.1999724	7
	notochord	Position 3	-0.0684302	-0.09438415			0.1252801	6
	notochord	Position 4	-0.12836284	-0.12644322			0.17251	6

Supplemental Table 6. Statistics on mean, median, standard deviation, and number of trials for dorsoventral bending trials. Maximum load and work during a trial were always observed in cycle 1.

Dorsoventral Bending								
	**Dissection Condition**	**Position**	**mean**	**median**	**Min**	**Max**	**sd**	**n**
Youngs Modulus (*E*, GPa)								
	column	Position 1	1.348609684	0.864830134	428234241	3.704E+09	1167689544	10
	column	Position 2	0.953918990	0.563925679	429567818	2.571E+09	681710619	9
	column	Position 3	1.148559402	0.679562658	293307164	3.28E+09	1071881602	7
	column	Position 4	5.519552759	5.52E+09	386661489	1.065E+10	7259004449	2
	notochord	Position 1	1.067030063	1.014E+09	119972357	1.833E+09	640433269	7
	notochord	Position 2	0.553990365	0.580496140	-378614664	1.354E+09	549184977	7
	notochord	Position 3	1.256875143	0.989625712	269801912	2.684E+09	932084386	6
	notochord	Position 4	0.124165072	0.56727223	-842862074	1.313E+09	787225971	6
Flexural Rigidity (*EI*, Nm^2)								
	column	Position 1	1.533799858	1.129345	0.74831553	3.1718226	0.80901577	10
	column	Position 2	1.259129853	1.0765099	0.75064588	2.225695	0.56466307	9
	column	Position 3	1.133138554	0.8949673	0.65792513	2.4464535	0.65686574	7
	column	Position 4	1.292268777	1.2922688	0.39060254	2.193935	1.27514862	2
	notochord	Position 1	0.065850077	0.0299834	0.00763231	0.2159803	0.07447279	7
	notochord	Position 2	0.034482241	0.0369296	-0.01161575	0.0861547	0.03325896	7
	notochord	Position 3	0.040041553	0.0303613	0.01648655	0.0823368	0.02689325	6
	notochord	Position 4	0.007901358	0.0016497	-0.01059172	0.0402729	0.01928082	6
Max Load (N) - Cycle 1								
	column	Position 1	1.9172498	1.4116812	0.93539441	3.9647782	1.01126971	10
	column	Position 2	1.5739123	1.3456373	0.93830735	2.7821188	0.70582884	9
	column	Position 3	1.4164232	1.1187091	0.82240641	3.0580668	0.82108218	7
	column	Position 4	1.615336	1.615336	0.48825318	2.7424188	1.59393577	2
	notochord	Position 1	0.1646252	0.0749584	0.01908078	0.5399507	0.18618198	7
	notochord	Position 2	0.0862056	0.0923239	-0.02903938	0.2153867	0.0831474	7
	notochord	Position 3	0.1001039	0.0759033	0.04121639	0.2058421	0.06723313	6
	notochord	Position 4	0.0197534	0.0041242	-0.02647929	0.1006822	0.04820204	6
Work (Nm) - Cycle 1								
	column	Position 1	4.198395825	3.2606717	1.81442025	8.7225	2.2010991	10
	column	Position 2	3.043112523	2.9755896	1.52184633	5.2447838	1.3639927	9
	column	Position 3	2.896039345	2.3962959	1.70199506	6.1447794	1.5578646	7
	column	Position 4	2.748890615	2.7488906	0.43022771	5.0675535	3.2790845	2
	notochord	Position 1	0.164165206	-0.11404	-0.19733654	0.7911684	0.4212756	7
	notochord	Position 2	0.003895958	0.0315476	-0.26293989	0.3039641	0.2174923	7
	notochord	Position 3	0.039487002	0.0204053	-0.07231483	0.2487407	0.1189621	6
	notochord	Position 4	-0.21236608	-0.219161	-0.39258611	-0.001043	0.1298716	6

Supplemental Table 7. Statistics on mean, median, standard deviation, and number of trials for torsional trials.

**Torsion**								
	**Dissection Condition**	**Position**	**mean**	**median**	**Min**	**Max**	**sd**	**n**
Torsional Modulus (*G*, GPa)								
	column	1	2.095632988	8.110153514	4.06E+05	4.06E+05	2.45	10
	column	2	3.947752323	4.008058229	4.06E+05	4.06E+05	1.80	9
	column	3	4.343170745	4.418865876	4.06E+05	4.06E+05	2.22	9
	column	4	5.743387444	5.152011880	4.06E+05	4.06E+05	2.80	8
	notochord	1	0.0002442956	0.0001985393	4.06E+05	4.06E+05	0.000193	6
	notochord	2	0.0002105376	0.0000194384	4.06E+05	4.06E+05	0.0000547	7
	notochord	3	0.0002497075	0.0002446083	4.06E+05	4.06E+05	0.0000724	6
	notochord	4	0.0003000598	0.0002575233	4.06E+05	4.06E+05	0.0000808	7
Torsional Rigidity (*GJ*, Nm^2)								
	column	1	3.27E-04	1.16E-04	8.51E-05	9.75E-04	3.50E-04	10
	column	2	5.61E-04	5.30E-04	2.84E-04	1.04E-03	2.45E-04	9
	column	3	2.80E-04	2.56E-04	2.05E-04	4.01E-04	6.96E-05	9
	column	4	1.61E-04	1.68E-04	8.30E-05	2.59E-04	5.74E-05	8
	notochord	1	1.86E-05	1.11E-05	6.54E-06	6.21E-05	2.14E-05	6
	notochord	2	2.08E-05	1.96E-05	1.29E-05	2.89E-05	5.41E-06	7
	notochord	3	1.70E-05	1.59E-05	1.33E-05	2.27E-05	3.43E-06	6
	notochord	4	1.52E-05	1.41E-05	1.01E-05	2.17E-05	4.69E-06	7
Torsional Stiffness (*GJ*L, Nm)								
	column	1	0.005598229	0.001846384	0.0014939	0.0164825	6.26E-03	10
	column	2	0.01138235	0.010005812	0.0053822	0.0225285	5.23E-03	9
	column	3	0.005473051	0.005015862	0.0040951	0.0080161	1.38E-03	9
	column	4	0.003051216	0.003227748	0.0016021	0.0047888	1.01E-03	8
	notochord	1	0.000794285	0.000405587	0.0002617	0.0028226	9.96E-04	6
	notochord	2	0.000435213	0.000406528	0.0002865	0.0007812	1.64E-04	7
	notochord	3	0.00038309	0.000380343	0.0002667	0.0005169	8.11E-05	6
	notochord	4	0.000375321	0.000389023	0.0002733	0.0004515	6.84E-05	7
Torque (Nm)								
	column	1	0.002931302	0.000966796	0.0007822	0.0086304	3.28E-03	10
	column	2	0.005960019	0.005239197	0.0028182	0.0117963	2.74E-03	9
	column	3	0.002865786	0.002626402	0.0021442	0.0041974	7.22E-04	9
	column	4	0.001597676	0.001690115	0.0008389	0.0025075	5.31E-04	8
	notochord	1	0.000415899	0.00021237	0.000137	0.001478	5.22E-04	6
	notochord	2	0.000216316	0.000210524	0.00015	0.0003361	6.13E-05	7
	notochord	3	0.000200586	0.000199148	0.0001397	0.0002706	4.25E-05	6
	notochord	4	0.000195785	0.000203691	0.0001431	0.0002364	3.68E-05	7

Supplemental Table 8. Sources for vertebral reconstructions (Extended Data Fig. S5) and skull measurements (Fig. S3). W. = Westoll Collection, University of Newcastle upon Tyne; B.M. = British Museum (Natural History); R.S.M. = Royal Scottish Museum, Edinburgh; NUFV = Nunavut Fossil Vertebrate Collection, currently housed at Canadian Museum of Nature; CMNH, Carnegie Museum of Natural History; MGUH = Geological Museum, University of Copenhagen; FMNH = Florida Museum of Natural History; MCS, Museum of Comparative Zoology, Harvard University.

**Taxa**	**Specimens referenced for vertebrae***	**Additional specimens referenced for skull**	**Sources**
**Eusthenopteron foordi**	W. 659, B.M. P. 6794, & R.S.M. 1897.51.188	Hitchcock (1992) reconstruction from MHNM 6-12-196, ROM 1234	Vertebrae: Andrews and Westoll, 1970; Hitchcock, 1995<br>Skull: Hitchcock, 1992
Tiktaalik roseae	NUFV 1008	NA	Stewart et al., 2024
Greererpeton burkemorani	CMNH 11069 and CMNH 11068	NA	Vertebrae: Godfrey, 1989a<br>Skull: Godfrey, 1989b
Acanthostega gunnari	MGUH f.n. 1227: X primarily, Y&Z as well	Clack composite reconstruction from MGUH f.n. 1227, 1300, 1305, and 1604	Vertebrae: Coates, 1996<br>Skull: Clack, 1994
Whaatcheeria deltae	FMNH PR 1634 primary, 1816 & 1700 secondary	NA	Vertebrae: Otoo *et al.*, 2021<br>Skull: Rawson *et al.*, 2021
Trimerorhachis insignis	MCZ 16009 B, primary. MCZ 16009D, secondary (formerly UCMP)	Milner & Shoch composite reconstruction from MCZ 1169 and AMNH 4565	Vertebrae: Case, 1911 & 1935; Shishkin, 2000; Pawley, 2007<br>Skull: Milner & Schoch, 2013

*specimen numbers from referenced publications

Supplemental Table 9. The influence of axial column proportions on mobility. Comparison of bending calculation along 10 cm of whole axial column with equivalent force for deflection applied and proportions of each taxon's column scaled to the size of the column of *Eusthenopteron foordi* by "Base" (i.e., width).

**Taxon**	**Lateral Flexion (°) resulting from an applied force of 1.44E+03 N**	**Doral Extension (°) resulting from an applied force of 4.13E+03 N**	**Torque (Nm) necessary for 5° of rotation**
Eusthenopteron foordi	10	10	116
Tiktaalik roseae	10.9	13.05	93.8
Acanthostega gunnari	7.85	4.88	210.5
Whatcheeria deltae	10.25	10.75	109.4
Greererpeton burkemorani	13.2	22.6	61.1
Trimerorhachis insignis	0.54	0.526	637

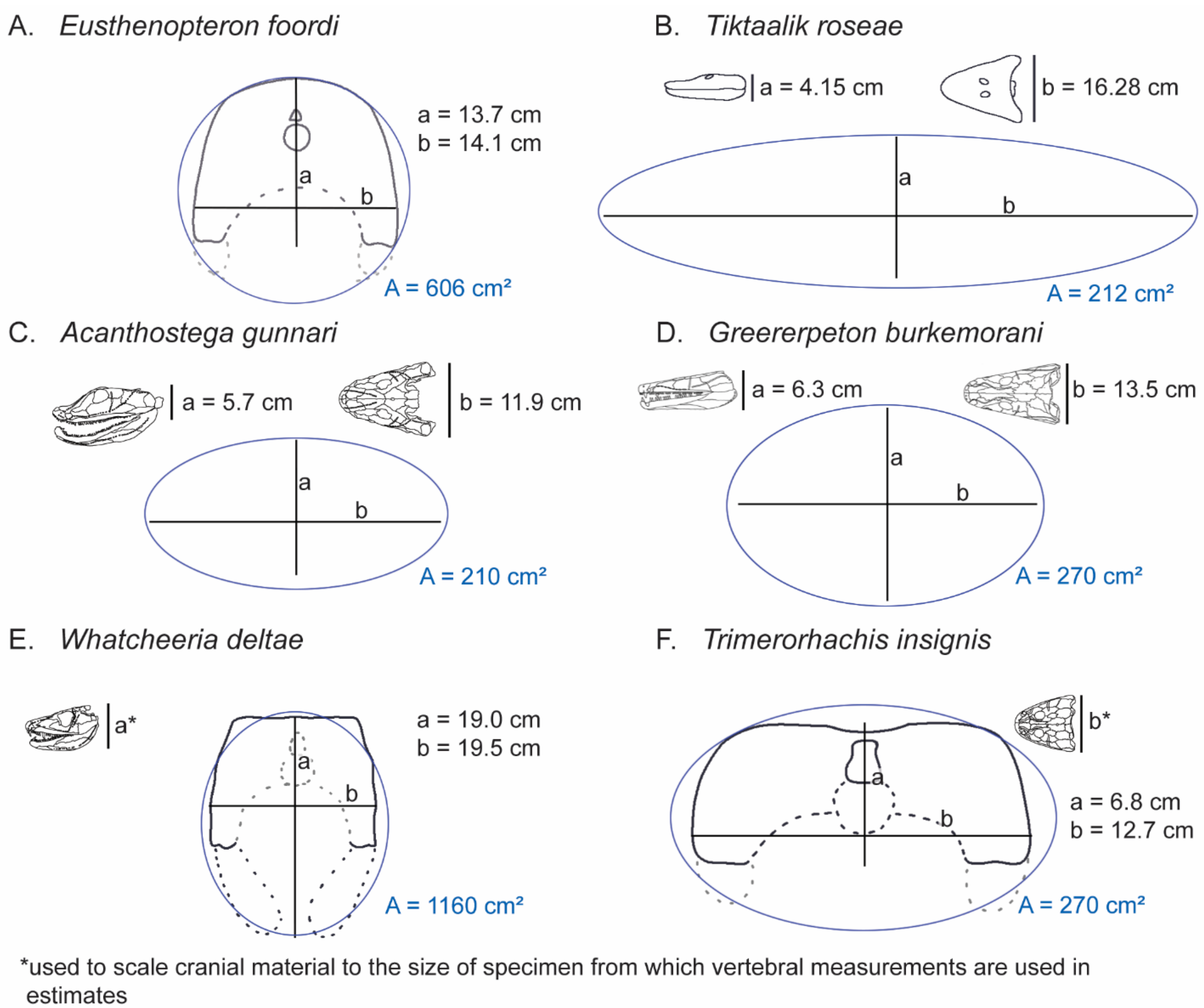

Supplemental Figure 3. Maximum muscle areas for extinct taxa correspond to hemispheres. Images produced with reference to A. Hitchcock (1995). B. Stewart *et al.* (2024). C. BKAO & Clack (1994). D. Godfrey (1989b). E. BKAO & Rawson *et al.* (2021). F. Milner & Schoch (2013), BKAO, & Case (1935).

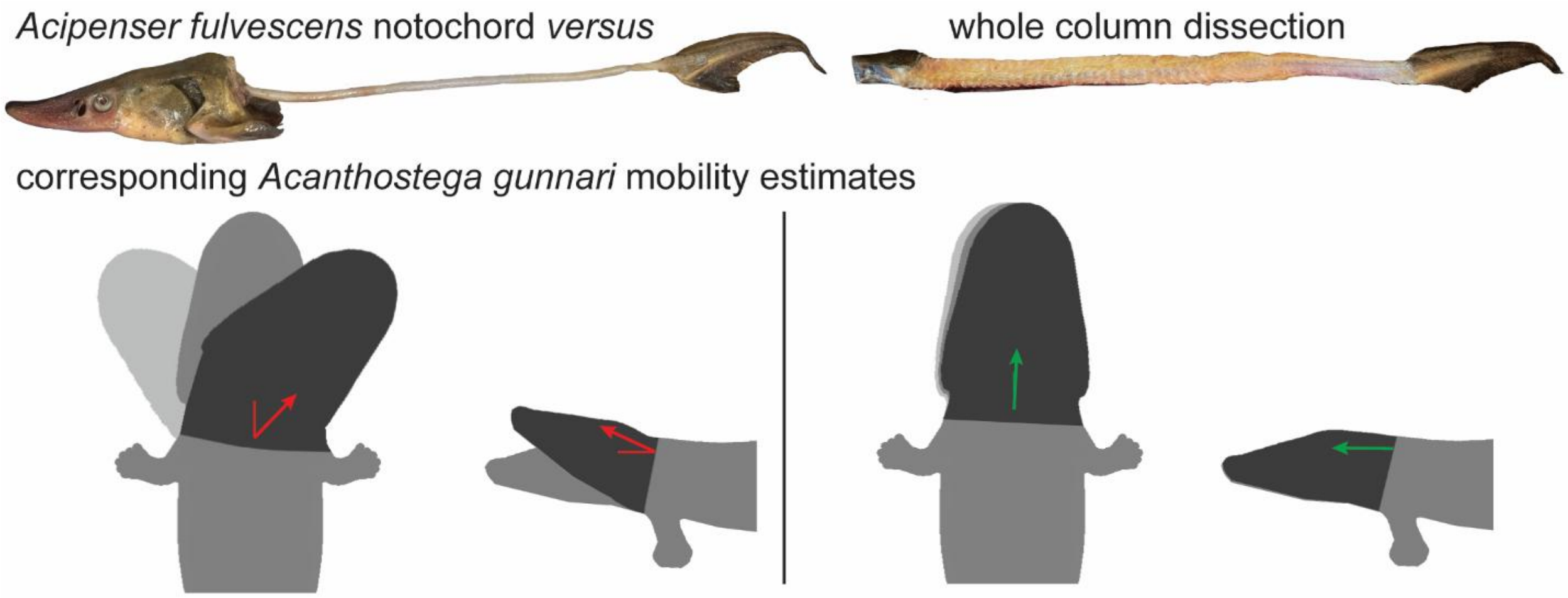

Supplemental Figure 4. Range of lateral flexion and extension estimates in the neck as limited by the notochord alone (left) and the whole axial column (right) demonstrate that notochordal stiffness is not the limiting factor in axial column mobility.

References, Supplemental Information:

1. Blaker, C. L., Ashton, D. M., Hartnell, N., Little, C. B., & Clarke, E. C. Tendon biomechanical properties are altered by storage duration but not freeze-thaw temperatures or cycles. *Journal of Orthopaedic Research* **42**(6), 1180-1189 (2024).

2. Case, E. C. Revision of the Amphibia and Pisces of the Permian of North America. *Carnegie Institute of Washington Publication* 1–179 (1911).

3. Case, E. C. Description of a collection of associated skeletons of *Trimerorhachis*. *University of Michigan Contributions from the Museum of Paleontology* **4**, 227–274 (1935).

4. Changoor, A., Fereydoonzad, L., Yaroshinsky, A., & Buschmann, M.D. Effects of refrigeration and freezing on the electromechanical and biomechanical properties of articular cartilage. *Journal of Biomechanical Engineering* **132**(6), 064502 (2010).

5. Clack, J. A. *Acanthostega gunnari*, a Devonian tetrapod from Greenland; the snout, palate and ventral parts of the braincase, with a discussion of their significance. *Meddelelser om Grønland: Geoscience* **31**, 1–24 (1994).

6. Coates, M. I. The Devonian tetrapod *Acanthostega gunnari* Jarvik: postcranial anatomy, basal tetrapod interrelationships and patterns of skeletal evolution. *Transactions of the Royal Society of Edinburgh: Earth Sciences* **87**, 363–421 (1996).

7. Dhiman, M. S., Salaam, M. A., Bader, T. J., Jacobs, W. B., Thomas, K. C., Salo, P. T., Hart, D.A., Swamy, G., & Duncan, N. A. Freezing negatively impacts the interlamellar mechanical properties of the degenerated human annulus fibrosus. *Journal of Biomechanics* **188**, 112779 (2025).

8. Godfrey, S. J. The postcranial skeletal anatomy of the Carboniferous tetrapod *Greererpeton burkemorani* Romer, 1969. *Philosophical Transactions of the Royal Society B: Biological Sciences* **323**, 75–133 (1989a).

9. Godfrey, S. J. Ontogenetic changes in the skull of the Carboniferous tetrapod *Greererpeton burkemorani* Romer, 1969. *Philosophical Transactions of the Royal Society of London. B, Biological Sciences* **323**(1213), 135-153 (1989b).

10. Hitchcock, E. C. A functional interpretation of the anterior-most vertebrae and skull of *Eusthenopteron*. *Bulletin du Muséum national d'Histoire naturelle, 4ème série–section C–Sciences de la Terre, Paléontologie, Géologie, Minéralogie* **4**, 269–285 (1995).

11. Kaye, B., Randall, C., Walsh, D., & Hansma, P. The effects of freezing on the mechanical properties of bone. *Open Bone Journal* **4**(1), 14-19 (2012).

12. Milner, A. R., & Schoch, R. R. *Trimerorhachis* (Amphibia: Temnospondyli) from the Lower Permian of Texas and New Mexico: cranial osteology, taxonomy and biostratigraphy. *Neues Jahrbuch für Geologie und Paläontologie, Abhandlungen* **270**, 91-128 (2013).

13. Otoo, B. K. A., Bolt, J. R., Lombard, R. E., Angielczyk, K. D. & Coates, M. I. The postcranial anatomy of *Whatcheeria deltae* and its implications for the family Whatcheeriidae. *Zoological Journal of the Linnean Society* **193**, 700–745 (2021).

14. Rawson, J. R., Porro, L. B., Martin-Silverstone, E., & Rayfield, E. J. Osteology and digital reconstruction of the skull of the early tetrapod *Whatcheeria deltae*. *Journal of Vertebrate Paleontology* **41**(2), e1927749 (2021).

15. Shishkin, M. A. Evolution of the Cervical Vertebrae in Temnospondyl Amphibians and Differentiation of the Early Tetrapods. *Paleontological Journal* **34**, 534–546 (2000).

16. Stewart, T. A., Lemberg, J. B., Hillan, E., Magallanes, I., Daeschler, E. B. & Shubin, N. H. The axial skeleton of *Tiktaalik roseae*. *PNAS* **121**(15), e2316106121 (2024).
