# Supplementary material for: Early tetrapod neck mobility modelled using an extant analogue
Source: Nature. 2026 Oct 14;659(8139):128–32. doi: 10.1038/s41586-026-11158-3 (PMC13736659; doi:10.1038/s41586-026-11158-3)
Supplement: Supplementary file 2 [file 41586_2026_11158_MOESM2_ESM.pdf]

nature portfolio

Corresponding author(s): Dana E. Korneisel

Last updated by author(s): Aug 28, 2026

Reporting Summary

Nature Portfolio wishes to improve the reproducibility of the work that we publish. This form provides structure for consistency and transparency in reporting. For further information on Nature Portfolio policies, see our Editorial Policies and the Editorial Policy Checklist.

Statistics

For all statistical analyses, confirm that the following items are present in the figure legend, table legend, main text, or Methods section.

n/a	Confirmed	
☐	☒	The exact sample size (*n*) for each experimental group/condition, given as a discrete number and unit of measurement
☐	☒	A statement on whether measurements were taken from distinct samples or whether the same sample was measured repeatedly
☒	☐	The statistical test(s) used AND whether they are one- or two-sided<br>*Only common tests should be described solely by name; describe more complex techniques in the Methods section.*
☒	☐	A description of all covariates tested
☒	☐	A description of any assumptions or corrections, such as tests of normality and adjustment for multiple comparisons
☐	☒	A full description of the statistical parameters including central tendency (e.g. means) or other basic estimates (e.g. regression coefficient) AND variation (e.g. standard deviation) or associated estimates of uncertainty (e.g. confidence intervals)
☒	☐	For null hypothesis testing, the test statistic (e.g. *F*, *t*, *r*) with confidence intervals, effect sizes, degrees of freedom and *P* value noted<br>*Give P values as exact values whenever suitable.*
☒	☐	For Bayesian analysis, information on the choice of priors and Markov chain Monte Carlo settings
☒	☐	For hierarchical and complex designs, identification of the appropriate level for tests and full reporting of outcomes
☒	☐	Estimates of effect sizes (e.g. Cohen's *d*, Pearson's *r*), indicating how they were calculated

Our web collection on statistics for biologists contains articles on many of the points above.

Software and code

Policy information about availability of computer code

Data collection	Programs for data collection on the Universal Testing Machine were written by CMD with modifications by LH. The parameters are listed in the extended data methods section. Blender (version 4.0) was used for linear measurements on 3D data. Fiji (version #####) was used for linear measurements on 2D data from fossil material and sturgeon. Fiji (version v1.54p) was used during revision to check measurements.
Data analysis	Code was written by CMD in R (version 4.3.3) used with RStudio (version 2023.12.1) using code written for the CrushR package with some functions specialized for use in this study (the base package, previously published is available at github: https://github.com/CDonatelli/CrshR, all specialized code for this study is also present in the following repository). Data and additional code for their analysis as well as the specialized code using CrushR are available in the github repository Korneisel_TetNecks_2026 which can be accessed through https://github.com/CDonatelli/Korneisel_TetNecks_2026 or through Figshare with the identifier "10.6084/m9.figshare.33552199" or using the link: https://figshare.com/articles/dataset/Korneisel_TetNecks_2026/33552199?file=68415544. The R packages ggplot2 (version 4.0.1), ggthemes (version 5.1.0.9000), readr (version 2.1.5), dplyr (version 1.1.4), and RColorBrewer (version 1.1-3) are used in data analysis and plotting.

For manuscripts utilizing custom algorithms or software that are central to the research but not yet described in published literature, software must be made available to editors and reviewers. We strongly encourage code deposition in a community repository (e.g. GitHub). See the Nature Portfolio guidelines for submitting code & software for further information.

Data

Policy information about availability of data

All manuscripts must include a data availability statement. This statement should provide the following information, where applicable:

- Accession codes, unique identifiers, or web links for publicly available datasets
- A description of any restrictions on data availability
- For clinical datasets or third party data, please ensure that the statement adheres to our policy

The original data for this study are available within the GitHub repository https://github.com/CDonatelli/Korneisel_TetNecks_2026, owned and managed by CMD, presented in excel spreadsheets.

Research involving human participants, their data, or biological material

Policy information about studies with human participants or human data. See also policy information about sex, gender (identity/presentation), and sexual orientation and race, ethnicity and racism.

Reporting on sex and gender	No human participants or material were used in this study.
Reporting on race, ethnicity, or other socially relevant groupings	No human participants or material were used in this study.
Population characteristics	No human participants or material were used in this study.
Recruitment	No human participants or material were used in this study.
Ethics oversight	No human participants or material were used in this study.

Note that full information on the approval of the study protocol must also be provided in the manuscript.

Field-specific reporting

Please select the one below that is the best fit for your research. If you are not sure, read the appropriate sections before making your selection.

☒ Life sciences ☐ Behavioural & social sciences ☐ Ecological, evolutionary & environmental sciences

For a reference copy of the document with all sections, see nature.com/documents/nr-reporting-summary-flat.pdf

Life sciences study design

All studies must disclose on these points even when the disclosure is negative.

Sample size	Ten Acipenser fulvescens specimens were used in this study as each could be used for multiple measurements along the length of the body as well as for three different types of movement. It is imperative for biomechanical study that the specimens be tested as freshly as possible and in as similar a condition as possible. The ultimate sample size was limited for this reason and because dissection of each sample before and during testing was very time consuming, limiting each testing day to 3-4 specimens.
Data exclusions	Data were excluded when there were issues during the collection cycle (see extended data methods section) such as the specimen falling over during testing. Exclusion criteria were pre-established in the experimental design and data that would need to be excluded were identified during testing.
Replication	Testing took place on three separate days but each material test was performed once at each location except in cases where the specimen fell out of alignment (in these cases, the test was attempted a second time). These tests were repeated at the same locations on multiple specimens (see extended data tables 1-3 for process and numbers). All details of specimen preparation for testing including detailed dissection images are provided in the extended data to make the precise testing conditions repeatable by any interested party.
Randomization	This study did not allocate specimens into different experimental groups as it was focused on collecting non-experimental biomechanical data.
Blinding	This study did not involved experimental groups, so no blinding protocol was used.

Reporting for specific materials, systems and methods

We require information from authors about some types of materials, experimental systems and methods used in many studies. Here, indicate whether each material, system or method listed is relevant to your study. If you are not sure if a list item applies to your research, read the appropriate section before selecting a response.

Materials & experimental systems

n/a	Involved in the study
☒	☐ Antibodies
☒	☐ Eukaryotic cell lines
☐	☒ Palaeontology and archaeology
☐	☒ Animals and other organisms
☒	☐ Clinical data
☒	☐ Dual use research of concern
☒	☐ Plants

Methods

n/a	Involved in the study
☒	☐ ChIP-seq
☒	☐ Flow cytometry
☒	☐ MRI-based neuroimaging

Palaeontology and Archaeology

Specimen provenance	All specimens utilized in this study were collected and described by previous researchers. Measurements were taken from published data and the source publications are listed in the references sections as well as in figure captions where relevant.
Specimen deposition	All specimens utilized in this study were described by previous researchers and are in collections readily available to researchers. Their accession information can be found in the relevant referenced articles.
Dating methods	No new dates are provided in this study.

☐ Tick this box to confirm that the raw and calibrated dates are available in the paper or in Supplementary Information.

Ethics oversight	No ethical approval or guidance was necessary for this study as the palaeontological specimens used herein did not require it as all material is already present in the literature.

Note that full information on the approval of the study protocol must also be provided in the manuscript.

Animals and other research organisms

Policy information about studies involving animals; ARRIVE guidelines recommended for reporting animal research, and Sex and Gender in Research

Laboratory animals	This study did not include any laboratory animals.
Wild animals	The Acipenser fulvescens individuals used in this study were sourced after euthanasia at approximately 1 year of age. They were originally obtained from the wild as fertilized eggs by members of the Rainy Rivers First Nation in collaboration with the Ontario Aquaculture Research Center and raised at the research center before being moved by ground transport to Wilfrid Laurier University where they were housed until reaching approximately one year of age. After experimentation there, the remains of euthanized fish were provided to us.
Reporting on sex	Sex was not considered in this study design and the fish were not sexed during testing. There is no reason to believe that sex has any effect on the axial column biomechanics of Acipenser fulvescens.
Field-collected samples	This study did not involve any newly collected animals from the field. See 'wild animals' section for more information on specimen provenance.
Ethics oversight	Neither ethical guidance nor approval were necessary for this study as the study organisms were all sourced dead. The fish used in this study that were not sourced from waste from commercial fishing were housed and cared for in accordance with the Canadian Council on Animal Care and Wilfrid Laurier University's Animal Care Committee approved animal use protocol (AUP #22003).

Note that full information on the approval of the study protocol must also be provided in the manuscript.

Plants

Seed stocks	No plants were used in this study.
Novel plant genotypes	No plants were used in this study.
Authentication	No plants were used in this study.
