# Supplementary material for: Early tetrapod neck mobility modelled using an extant analogue
Source: Nature. 2026 Oct 14;659(8139):128–32. doi: 10.1038/s41586-026-11158-3 (PMC13736659; doi:10.1038/s41586-026-11158-3)
Supplement: Supplementary file 3 [file 41586_2026_11158_MOESM4_ESM.pdf]

nature portfolio

# Peer Review File

# Early tetrapod neck mobility modeled from an extant analogue

Corresponding Author: Dr Dana Korneisel

Any redactions in this file are there to maintain patient confidentiality, the confidentiality of unpublished data, or to remove third-party material.

**This file contains all reviewer reports in order by version, followed by all author rebuttals in order by version.**

Version 0:

Reviewer comments:

Referee #1

(Remarks to the Author)
Summary of key results
This study proposes that early/stem tetrapods had a notochord-dominated axial column, whose flexibility is determined by the material properties of soft tissues, rather than the mobility of bony intervertebral joints. Because notochord-dominated axial columns are relatively rare in living vertebrates, it has been hard to accurately assess the mobility of these early necks. Specifically, did the head-shoulder decoupling that gave rise to the neck increase mobility of the head relative to the trunk? This study tests the hypothesis that head mobility was the functional driver for head-shoulder separation.
This study measured stiffness of the axial column in lake sturgeon, which also have a notochord-dominated axial column. They used this stiffness, along with the dimensions (second moment of area, length) of the neck in 6 early/stem tetrapods to estimate maximum neck mobility in these extinct species. The results showed that, unless the neck was relatively long, neck mobility was low: <10 (usually <5) degrees of dorsal or lateral bending – even in species with a de-coupled head and shoulders.
The authors conclude that independent head mobility (including for feeding) was not a functional driver for separation of the head and neck. Instead, head-shoulder decoupling was likely driven by locomotion functions, and neck mobility primarily increased through the evolution of longer necks and mobile, bony intervertebral joints.

Originality and significance
I want to specifically applaud the authors for:
1) The use of sturgeon as a novel but appropriate model for early/stem tetrapods. It is particularly well-suited as one of the few living vertebrates whose axial column is dominated by notochord rather than bony joints.
2) Measuring empirical, mechanical data from living species to test hypotheses and inform predictions about extinct species. This topic—the origin of the neck and its function, mobility in early/stem tetrapods—is widely interesting (as demonstrated by the large number of papers in this area). However relatively few apply quantitative, mechanical analysis of living analogues, so this subject is compelling and noteworthy to many people from a range of disciplines, e.g., biomechanics, evolution, palaeontology, ecology.

Validity and conclusions
1. The modelling used here, while logical, has not been validated on extant species. The conclusions of this study rely on knowing: how well do measurements of axial column material properties and dimensions predict the maximum potential (or even better, in vivo) bending of the neck or similar region of the spine? This could be done relatively easily by measuring bending and torsion within the same species, the lake sturgeon. It would be even more robust to validate it across multiple species (see also next point). Without this validation, it is difficult to assess the validity and degree of uncertainty in the bending estimated for extinct species.
2. The conclusions also rely on notochord/soft-tissue properties being similar across actinopterygian and sarcopterygian fishes. Is there evidence that lake sturgeon are representative across this range? I was somewhat surprised this study did not use lungfish, which also have a notochord-dominated axial column, but are more closely related to early/stem tetrapods. If the study were repeated on another extant species, would the conclusions change? This could be addressed by measuring the material properties of the axial column in one or more additional species. Or explaining why A) there are good

reasons to expect these properties are similar across species, or B) no other extant species could be tested or is as appropriate a model for early/stem tetrapods.

Data and Methodology
For the stem and early tetrapods, how many and which vertebrae were measured for second moment of area calculations? From Supplemental Figure 7, at least vertebral height—if not width—varies along the cervical column. So were dimensions taken from a single vertebra or averaged from multiple vertebrae averaged? I couldn't find this information, which would be essential to reproduce the results:

Clarity
The paper would benefit greatly from clearly stating how the location and mobility of the head, neck, and body are being defined. Specifically the following terms:
'total head motion' (what does this include?)
'rotational head turning' (rotation in which axis?)
'neck'
'head mobility'
'neck bending' vs 'trunk bending'
For example, if head mobility is defined as motion relative to the shoulder, then the separation of the head and shoulder girdle must always increase the head's mobility. Similarly, the distinction between 'neck' and 'trunk' is somewhat ambiguous in a study that includes at least two species with no clearly defined cervical region.

It would also be useful to explicitly state that you are not studying the neck as a joint-based system (as most other studies do). Instead, the assumption is that the neck functions as a single structure in bending, based on constat material properties.

Suggested Improvements
It would be useful to include a measure of bending per joint or unit of length to show how neck shape—not just length—impacts mobility. It is not greatly surprising that species with longer necks are predicted to have greater head mobility.

Please state what—if any—impact freezing and thawing is known to have on the material properties of soft tissues like the notochord.

Please include how many early/stem tetrapods had notochord-dominated axial columns (and are therefore well-represented by this study)?

The fused occipital vertebrae of sturgeon are mentioned, but not discussed or shown in detail. Please explain if/how these would be expected to impact mobility and justify why this doesn't impact the applicability of sturgeon as a model for stem and early tetrapods.

Please explain and justify why the modelling approach in this study doesn't take into account any variation in the morphology ossified elements of the cervical or anterior axial column.

(Remarks on code availability)
The provided code on the sturgeon data in the CrshR repository was tested and returned results similar to those presented in the paper. The manual selection of break and change points presents a potential source of variability, especially as there are no instructions on what is being selecte or why. Otherwise the code meets the specified requirements for reproducibility. However, detailed annotations in the code or a thorough README file would clear up uncertainties; the current README provides almost no instructions.

Referee #2

(Remarks to the Author)
I co-reviewed this manuscript with one of the reviewers who provided the listed reports.

(Remarks on code availability)
I was able to use and run the code, however there is no README file with sufficient instructions provided for others without former knowledge of RStudio.

Referee #3

(Remarks to the Author)
This manuscript entitled "Early tetrapod neck mobility modeled from an extant analogue" by Dana E. Korneisel and colleagues examined the mobility of the neck, which was newly evolved anatomical part between the head and shoulder in the stem tetrapods, based on the biomechanical properties of the notochord, and found that the neck mobilities were limited in early limbed tetrapods. For the past 20 years, our understanding of the transition from fish to tetrapods has made great progress, particularly in the morphological and functional changes from fins to limbs. On the other hand, our understanding of the function of the neck in the lineage leading to tetrapods has remained poor, and this study is the first to do so. The methodology of this study represents the best approach to quantitatively estimating neck mobility from the fossil data currently available and is highly convincing. This finding will be of interest to many scientists as it is important for

understanding how tetrapods evolved from fish and how our body became established. Therefore, I believe this study is worth publishing from Nature, although there is room for improvement before publication.

Major comments.

(1) Background of this study: neck.
The morphological differences between necked crown tetrapods and neckless fishes should be better explained. In vertebrate morphology, the term "neck" has been used by various researchers to refer to structures found in various taxa. For example, the term "neck" is often used to describe the gap between the head and shoulder of placoderms and the other fishes. On the other hand, the "neck" in the human body is a region lacking internal organs (i.e., a coelom), and is distinct from that of amphibians. It should be called the "amniote neck" (see Hirasawa et al., 2016). In amphibians, the heart is located in the "neck." The "neck" used in this study is used to refer to the structure that constitutes the body plan of crown tetrapods, and should be called the "tetrapod neck." It would be better to explain how it evolved from the fish to the amniote conditions (this should be explained in the diagram, see Comment (5)).

It's debatable whether the evolutionary process covered in this paper is appropriately called "head-shoulder decoupling." I believe the "decoupling" referred to here is functional, not developmental, but the authors don't even explain this. As the results of this study, neck mobility was shown to be quite limited, so I suppose that (functional) "decoupling" is not appropriate. I think the term "separation," used elsewhere in this paper, is a more appropriate description of this evolution.

Hirasawa, T., S. Fujimoto, and S. Kuratani. 2016. Expansion of the neck reconstituted the shoulder-diaphragm in amniote evolution. Development, Growth & Differentiation 58: 143–153.

(2) Background of this study: notochord.
It should be more clearly explained that the evolutionary process focused by this study is the stage before the evolutionary establishment of the vertebral column. As mentioned in comment (5), the evolution of the axial skeleton can be better explained by illustrating it in a Figure.
Regarding the idea that the axial skeleton supported by the notochord is more flexible, it may be helpful to explain this by using the example of the dragonfish lineage, a teleost lineage in which the vertebral elements in the neck region were secondarily reduced and the skeleton was supported by the notochord (Schnell et al., 2010). In this lineage, the secondarily acquisition of the notochord-supported axial skeleton increased the mobility of the neck region.

Schnell, N. K., R. Britz, and G. D. Johnson. 2010. New insights into the complex structure and ontogeny of the occipito-vertebral gap in barbeled dragonfishes (Stomiidae, Teleostei). Journal of Morphology 271:1006–1022.

(3) Explanation of how to calculate neck lengths.
There is a lack of explanation for how the number of vertebrae corresponding to the neck region was determined, making it difficult to determine whether this is appropriate. For example, while the number of the neck vertebrae of Whatcheeria seems to be based on the detailed description by Otto et al. (2021), rather than simply accepting the number of "cervical vertebrae" given there, the study should provide a reproducible explanation of the criteria used to determine the number of neck bones (e.g., the presence or absence of developed ribs). Regarding Whatcheeria, ribs are also present in the areas considered to be vertebrae in the neck, so the neck length estimate may need to be left to a certain extent.

(4) Explanation of the relevance of choosing the sturgeon as a model.
In my opinion, there is no problem with using the sturgeon as a model for vertebrates with an axial skeleton supported by the notochord. However, the explanation for the choice of this taxon should be more detailed. It should also be explained whether the shared features seen in stem tetrapods and the sturgeon represent an ancestral state or a secondary related state (see comment (14)). It should also be specified what the "additional connective tissues" are (ligaments? fascia?) and whether they were also present in stem tetrapods.

(5) Main Figures
I believe a phylogenetic tree showing the evolution of notochords and the phylogenetic relationship of stem tetrapods should be included as a figure in the main text. The phylogenetic position of Trimerorhachis (temnospondyl), which is being analyzed in this study, should be explained properly, otherwise it will lead to misunderstandings. The branching of the lineage leading to the amniotes and the lineage leading to the lissamphibians, along with the phylogenetic position of the acquisition of the intervertebral joint, should be presented. The process of the head-shoulder separation should also be explained using lateral views of the skeleton.

Fig. 1 in the current manuscript includes illustrations of Eusthenopteron, Acanthostega, and Acipenser, but this is not informative, as the essential axial skeletal morphology is barely visible. Body silhouettes are unnecessary here. Rather, the skeletal elements (notochord and bones) should be clearly and in detail illustrated. Something like Supplementary Figure 7, but with the addition of notochord etc.

The way the data is presented in Fig. 2 is a little difficult to understand. It would be better to make the comparison of

estimated range of motion easier to understand than the silhouettes of the animals' bodies. A previous paper dealing with similar estimations, Pierce et al. (2012), is a good reference. (Incidentally, Pierce et al. (2012) also shares with this paper the use of a phylogenetically independent animal as a model for joint range of motion.) I believe this type of graphical comparison is better. Also, it would be better to compare both notochord-only estimates and whole axial column estimates on the same graph.

The phylogenetic tree in Fig. 2 in the current manuscript is very difficult to understand, so it would be better to show the phylogenetic relationships in Fig. 1 separately and use Fig. 2 to compare the estimated values graphically.

(6) Significant Figures of the Data
Throughout the paper, significant figures appear to be handled inappropriately. For example, it seems nearly impossible to measure fossil skeletons of the size under consideration with an accuracy of 0.01 mm, and such precision is not necessary, considering postmortem deformation of the fossils. Furthermore, while the actual measurements of material properties are given with very precise values, it is questionable whether these actually correspond to the accuracy of the measuring equipment.

(7) Anatomical direction terminology.
In vertebrate anatomy, directional terms are defined to describe body movement: rotation of the head is called "rotation" (or "axial rotation"), sideways bending is called "lateral flexion," and dorsal bending is called "extension." "Extension" in this case could be called "head elevation," but "bending" and "torsion" are not common in vertebrate anatomy, so I believe it would be better to revise the terminology.

Minor comments.

(8) Lines 22 and 26.
"co-ossified"
I don't understand why the authors say "co-ossified." It should simply be "ossified."

(9) Line 25.
"a soft-tissue dominated axial column is surprisingly rigid" should be changed into "a soft-tissue dominated axial column was surprisingly rigid"

(10) Lines 27, 56, 128, 131, 203, 219, and 220.
"the capacity to move their heads"
Here, "capability" is more appropriate than "capacity."

(11) Lines 28–30.
This sentence reads as if more-crownward stem tetrapods evolved from the tristichopterids, but this is not the case. It should be rewritten to be phylogenetically accurate.

(12) Lines 46.
"soft notochord" should be changed into "soft tissue notochord." At this point, data regarding hardness relative to other tissues are not cited. Thus, here the authors should just say "soft tissue" or "unmineralized."

(13) Line 49
"crown tetrapods today"
Remove "today." It's redundant.

(14) Line 57.
"phylogenetically distant but anatomically similar"
The phylogenetic distance is not an issue per se. What is important is whether they are of the same origin or independent. So, it should be rephrased as "phylogenetically independent but anatomically comparable."

(15) Lines 66, 67, 69, 129, and 147.
"early tetrapods" should be changed into "stem tetrapods." Trimerorachis should be excluded from this category.

(16) Line 90.
"in-tact"
Remove "-."

(17) Line 95.
"posterior precloacal axial column." (Add "precloacal.")

(18) Lines 99, 117, and 601.
The "m" in Young's modulus should be lowercase.

(19) Line 104.
"identified" should be changed into "focused on."

(20) Line 109.
"is" should be changed into "was."

(21) Lines 117–118.
The variables (E, I, and L) should be written in italics.

(22) Lines 120 and 611.
"the established pressures for vertebrate striated muscles"
"Established" sounds somewhat strange. "Known" is appropriate.

(23) Line 127.
"5.5–8.5º." These values correspond to Tiktaalik and Eusthenopteron, respectively, and should not be explained as a range.

(24) Line 148.
"34.2–37.6%." Use a dash rather than a hyphen.

(25) Lines 146–148.
"Whatcheeria is an outlier in neck length among early [stem] tetrapods, as its neck length relative to skull length is more than double that of other stem tetrapods."
What is the rationale for discussing neck length compared to skull length, and not skull width?

(26) Lines 202, 230, 246, and 248.
"head mobility" should be changed into "neck mobility."

(27) Line 204.
"peripheral" should be changed into "superficial."

(28) Lines 206–209.
This sentence should be simply rephrased as:
"Additional constraints on neck mobility could also come from the internal organs that would not allow extension or compression created by neck motion, such as the gill apparatus."
Based on the loss of postbranchial lamina (see Otto et al. (2021)), evolutionary changes in the gill apparatus should be included in the phylogenetic tree shown in [new] Fig. 1.

(29) Line 230.
"substantial." (Add "l.")

(30) Figure 1 caption.
"the general tetrapodomorph sarcopterygian condition"
"General" is not appropriate wording in evolutionary biology. Since it refers to the charactar state of the common ancestor, it should be called "ancestral." Also, the expression "tetrapodomorph sarcopterygian" is redundant. Therefore, it should be rephrased as "the ancestral tetrapodmorph condition" or "the ancestral condition of the tetrapodomorphs."

(31) Line 499.
"at a span of 4 cm" should be "at spans of 4 cm."

(32) Supplementary Table 2.
Positions 1–8 are confusing, so the positions should be shown on the body silhouette.

Tatsuya Hirasawa (The University of Tokyo)

(Remarks on code availability)

Referee #4

(Remarks to the Author)
General comments: This manuscript uses mechanical experiments on a modern analog and modeling data from fossils to address a fundamental question in the evolution of the tetrapod body plan: when did a truly mobile neck evolve? The approach the authors use is relatively simple, and is clever in its simplicity. They first use being experiments on modern sturgeon vertebral columns, which have a notochord-dominated morphology analogous to that observed in early tetrapods, to gain insight into the level of flexibility of the notochord itself as well as the column including bones and connective tissues. They then apply these values to models that simplify the shapes of the neck region of the early tetrapods and calculate the degree of bending that would be possible given estimates of the amount of force that the muscles acting on the neck could

generate. There are definitely some simplifying assumptions in terms of the modeled shapes of the vertebral columns and the the way they estimated the amount of muscle available to move the necks, but the assumptions generally seem reasonable and I suspect that their estimates are reasonably accurate.

Most of my comments below are relatively minor requests for clarifications or suggestions for small changes to the text and figures. Among these, maybe the most significant is the request for more information about what actual specimens were used in making the neck reconstructions for the fossil tetrapods. I also had one more significant question, specifically about whether bony stops (i.e., disarticulation of vertebrae where zygapophyses are present and/or bone-bone contact that would prevent further movement) would further constrain the ranges of motion they estimate. In part this question pertains to the sturgeon experiments (i.e., were there any cases where bone-bone contact seemed to be preventing further movement). It also pertains to the fossils, though, and is likely more important there because many of the fossils have more extensive articulations between vertebrae than is the case for the sturgeons. Given the very limited ranges of motion calculated, my guess is that bony stops are unlike to be much of a constraint, but I think this is an important thing to mention explicitly as it would speak to the overall plausibility of their results. Alternatively, it would be interesting to note if there are any cases where the size/orientations of articular surfaces suggest a range of motion that is greater than that suggested by the bending studies because this might suggest their results are overly conservative, although my guess is that's probably not the case. Finally, given that they have ranges of material property values for the extant sturgeons, it would be good to provide range of motion estimates for the fossil taxa that take this variation into account (e.g., range of motion estimates using, minimum, maximum, and mean values). That would provide a useful estimate of (one aspect) of uncertainty associated with the results.

Overall, this was an enjoyable paper to read and the results are interesting. It was also nice to see that the unusual neck proportions of Whatcheeria that were described a few years ago seem to have real functional implications.

Line 46: Maybe say soft tissue notochord instead of just soft. Regardless of the results you present here, I think it's generally known that the notochord is a somewhat rigid structure, and I don't think you want to imply that it is highly squishy.

Fig. 1: Are the schematic drawings of the heads/necks in parts a-c to scale? It would be good if they are because it would help support your statement in the text that the lake sturgeon is of comparable size to the relevant tetrapods. If the drawings are to scale, please note this in the figure caption. Also, since you mention supra-neurals, basidorsals and basiventrals in the text, I think it would be good to use those terms in the labels for the vertebra in part E so that readers can match the names with the structures.

Line 86: From what you say here and in the methods, it seems like the way you're approaching the problem is to estimate the stiffness the notochord/axial column of the fossil taxa and then predict how far the column could be bent given the muscle force available. That makes sense to some degree because in general there would be few bony stops that could limit bending in the sturgeon, so essentially you're carrying over the assumptions from the sturgeon tests to the tetrapod models. However, in the sturgeon experiments are there any points where the column can bend to a degree where contact between bony parts of successive vertebrae come into contact and therefore could actually serve as bony stops on range of motion? Or is the degree of bending required for that to happen so great that it is beyond plausible limits? Likewise, when thinking about the fossil taxa, some of them have relatively closely spaced central elements and zygapophyses that could function as bony stops on range of motion that are more akin to what's seen in more crownward tetrapods. Did you take those into account at all? Similarly, are the ranges of motion you predicted small enough to avoid causing disarticulation or unrealistic collisions/overlap of the zygapophyses?

Line 107: The reconstructions in the supplementary figure look good, but I think it would be helpful to include more information (presumably in a section in the supplement) that explains which specimens form the basis of the reconstructions. I realize that a lot of the information underlying them is taken from the literature, but even in those cases, I think noting (as much as possible) which specimens were used in the literature sources would help readers feel confident that the different parts of the vertebrae are scaled accurately relative to one another, understand if there is articulation information that helps to estimate spacing between vertebrae based on articulated specimens, the degree to which the cervical series is based on an actual series of vertebrae vs. repetition of a single vertebral morphology, etc.

Line 176: It would be good if you can provide some measurement of body size here to help contextualize the neck lengths you give. Also, neck length values and angular measurements of bending aren't given in table 1. It seems like you should call out table S5 here instead, which does provide those values.

Line 202: This would be a good place to discuss whether you would expect any impact of bony stops on range of motion for any of your included taxa, and if they were likely to come into play with the ranges of motion you calculated from shape/material properties alone.

Line 228: The wording here make it sound a bit like it's implying that the head/neck is being used for propulsion. I recommend replacing propulsion with locomotion.

Line 244: You could call the neck a spandrel in this context if you want to cite Gould and Lewontin.

Line 485: How does the size of the yearling sturgeon compare to the early tetrapods you're studying? i.e., are they comparable in size at that age, or do the fish have to grow more to achieve tetrapod-like sizes?

Line 538: Was there an original source for the drawings that were modified (e.g., from Coates' paper)? Same question for Trimerorhachis.

Line 582: Was a certain specific vertebral position used for the 'anteriormost' column? Or average values from a series of positions? Are these the positions you mention at line 588? If so, I would recommend moving that sentence up to this line.

(Remarks on code availability)
There is a REEADME file (code demo), but there wasn't code provided with the documents for review. I also didn't see a link for a repository with code, but maybe I missed it.

Version 1:

Reviewer comments:

Referee #1

(Remarks to the Author)
Overall, I appreciate the authors' thoughtful and positive approach to the reviews. They engaged meaningfully with my comments and made thorough revisions as a result. All of my original concerns have been sufficiently addressed and I do not have any further concerns or improvements.

The use of existing lungfish data (Gartner et al., 2022) is a clever way to validate the methods on in vivo data from extant species. Lungfish are available through the aquarium trade, so future studies would be able to acquire fresh (unfixed) specimens -- if scientifically and ethically justified.

The revised terminology for head, trunk, and neck motion provides more clarity, while avoiding the tricky issue of how, exactly, to define these regions (which I agree is a topic for a separate study/paper).

(Remarks on code availability)
As previously reported, the code can be installed and run. The improved comments and README file now make it much easier to use and understand this code.

Referee #2

(Remarks to the Author)
I co-reviewed this manuscript with one of the reviewers who provided the listed reports.

(Remarks on code availability)
The provided code works as intended and can be used to reproduce results.

Referee #3

(Remarks to the Author)
The revised version of the manuscript entitled "Early tetrapod neck mobility modeled from an extant analogue" by Dana E. Korneisel and colleagues has been improved from the previous version. I was mostly convinced with the authors' response to my comment. However, there still remain several points to be clarified, particularly concerning numerical values in primary data and significant figures, before publication.

Major comments

(1) Significant Figures of the Data
The authors' response to my previous comments was insufficient.
The neck length and notochord diameter of fossil species are described to have been measured at a resolution of 0.01 mm. It should be considered whether it is truly possible to measure objects on the scale of a few centimeters with this level of accuracy using photos and CT data, and if so, the method should be explained. When measuring from photographs, the angle of the specimen and the effects of lens aberrations must be taken into account, but it seems extremely difficult to obtain data indicating that "the notochord diameter of Acanthostega is 1.526 cm", for example. Furthermore, as is evident, in Extended Data Figure 8, attention has not been paid to the significant figures upon calculation. Although they do not affect the final conclusions of this study, clearly stating the level of precision used in the research is essential in scientific papers. In addition, while I understand that the zwickiLine Z5.0 TN used in the test is extremely accurate, the measurement accuracy should be described in the "Methods: Material Testing" section so that readers can understand it.

Minor comments

(2) Line 133
Remove "degrees."

(3) Figure 1
Fig. 1 in the new version is improved. However, there is a problem with how the ranges of taxa are illustrated on the phylogenetic tree. In the current version, all taxa are apomorphy-based, but the definitions of some taxa are branch-based (e.g., Tetrapodomorph). On the other hand, "Tetrapoda" is usually defined apomorphy-based, but based on that, it also includes stem tetrapods such as Acanthostega. The closest phylogenetic position to what the authors labeled would be "crown tetrapods". However, if "crown tetrapods" are to be illustrated, they must be labeled at the branching point of the common ancestor of amniotes and amphibians.
In panels A–C, only B. Eusthenopteron shows the shoulder girdle and fin skeletons; however, for consistency, the corresponding skeletal parts should also be shown in panels A and C.

(4) Extended Data "Measurements" section and Extended Data Figure 7
I understand that the method of identifying the cervical vertebrae in this paper has become clearer from the authors' responses to previous comments. However, I believe that Whatcheeria's explanation needs to be more specific, as shown in the response, rather than simply stating that it is based on "personal observation."

Tatsuya Hirasawa (The University of Tokyo)

(Remarks on code availability)

Referee #4

(Remarks to the Author)
General comments: It's good to see this paper progressing through the review process. Overall, I'm satisfied with the authors' responses to my comments and the changes they made to the manuscript to address them. I have a couple suggestions where maybe adding a sentence or two would further help to show that bony contacts don't play a major role in determining range of motion, but those should be very simple additions. Other than that there were a couple places where I think changes didn't get carried over as intended between drafts exactly as intended (e.g., the caption to figure 1), but those are just technical changes to make.

Figure 1 caption: The ordering of images of taxa in the figure and the A/B/C lettering described in the caption don't match up. It seems like the ordering from the previous version of the figure was inadvertently carried over.

Extended data figure 7: It didn't occur to me in the previous round of review, but the presence of Proterogyrinus and Archeria in this figure isn't really explained anywhere. Those taxa only show up in this figure and aren't referred to anywhere else in the main text or supplement that I can find aside from an oblique reference at line 213. I think the space that they use in the figure would be better spent on showing vertebrae for Eusthenopteron and Tiktaalik considering that they are discussed a lot in the text. I realize they show up a little in some of the other figures, but having them shown in a comparable way to the other taxa here would be helpful. If you do want to keep Proterogyrinus and Archeria in an expanded version of this figure (which also includes Eusthenopteron and Tiktaalik), then I recommend more explicitly referring to them somewhere in the text (maybe around line 51, where you talk about taxa where the vertebrae are more ossified and the notochord is reduced, and also specifically naming them when you mention embolomeres at line 213 to help readers who are unfamiliar with early tetrapod taxonomy make the connection between them).

Line 85: Change to: All contribute to the material properties of the column.

Line 97: You comments in the response to reviewers about bony stops not seeming to serve as a strong constraint in bending makes sense. I still think this is an important point to mention in the paper, though. This (line 97) seems like it might be an appropriate place to add a comment that you didn't observe this limiting bending in the sturgeon case. Somewhat related to this, you could also note that checking the the 3d orientations and sizes of the joint surfaces of those taxa would be an interesting next step; i.e., are the joints sized and shaped in a manner consistent with very limited ranges of motion? It would be a good test of your material properties/basic shape-based predictions. Line 216 seems like it would be a good place to include that discussion point.

Line 222: It might be interesting to mention the young sturgeon example you referenced in the rebuttal as an example of how the presence of osteoderms could significantly impact mobility (especially since at least some of the taxa in question did have bony scales, even if they weren't exactly like the osteoderms of sturgeons).

Line 486: Is 'material characterization material testing" redundant?

Line 643: Should 'depending' be in the sentence? Or is something missing after it?

(Remarks on code availability)

Version 2:

Reviewer comments:

Referee #3

(Remarks to the Author)
The authors responded sincerely to my comments. In particular, the accuracy of the measurement equipment has been thoroughly investigated and described in the Methods section; this study represents a significant contribution that will serve as a valuable reference for future research. However, I believe there are two points that should be addressed prior to publication. In all other respects, the manuscript is an excellent scientific paper.

Minor comments.
(1) Methods, Measurement section in the Main Text
Lines 546–547.
"Each intercentral span measurement was divided by 0.96 to estimate the notochord diameter" should be changed into " Each intercentral span measurement was multiplied by 0.96 to estimate the notochord diameter." (Notochord diameter = intercentral span x 0.96)

(2) Significant Figures of the Data
In some cells of Extended Data Table 7, the significant figures have not been corrected.
Furthermore, given the CT data resolution of 122.441 µm for *Tiktaalik*, a measurement should be expressed as "1.59 cm" (X.XX cm) rather than "1.585 cm" (X.XXX cm). This is because measurements on digital images are typically taken in pixel units (unless specialized techniques are employed); even assuming no manual error in pixel selection, the result falls between 129 pixels × 122.441 µm (1.5794889 cm) and 130 pixels × 122.441 µm (1.591733 cm). Consequently, the second decimal place falls within the margin of error, rendering the third decimal place meaningless. While this applies to two-dimensional measurements, measurements in Blender—compared to those in ImageJ (Fiji)—are subject to three-dimensional misalignment and interpolation (smoothing) during 3D model rendering; therefore, one generally cannot expect accuracy superior to that of two-dimensional measurements.

Tatsuya Hirasawa (The University of Tokyo)

(Remarks on code availability)

**Open Access** This Peer Review File is licensed under a Creative Commons Attribution 4.0 International License, which permits use, sharing, adaptation, distribution and reproduction in any medium or format, as long as you give appropriate credit to the original author(s) and the source, provide a link to the Creative Commons license, and indicate if changes were made.
In cases where reviewers are anonymous, credit should be given to 'Anonymous Referee' and the source.
The images or other third party material in this Peer Review File are included in the article's Creative Commons license, unless indicated otherwise in a credit line to the material. If material is not included in the article's Creative Commons license and your intended use is not permitted by statutory regulation or exceeds the permitted use, you will need to obtain permission directly from the copyright holder.
To view a copy of this license, visit https://creativecommons.org/licenses/by/4.0/

Dear Dr. Gee and Reviewers,

Thank you for your initial consideration of our manuscript, consideration “Early tetrapod neck mobility modeled from an extant analogue,” for publication. The comments from all of the reviewers were helpful in revising the manuscript, and I especially appreciated the attention to consistency in terminology throughout the text. I was so glad to see that the impact I saw in this project was apparent to all of you as well! I hope that you find our edits to have improved upon the initial submission with any declined edits fairly considered.

We have acted on both of the two main requests, for validation of the model against lungfish data and for the addition of code annotations and a README file to the github repository. CMD maintains the repository and has handled the code. We were able to acquire CT scans of two lungfish from a previous biomechanical study by Dr. Samantha Gartner (Gartner et al., 2022) and her XROMM model from which cranial elevation were measured in the original publication (peaking at just under five degrees). Repeating our modeling method on each of the two lungfish from measurements on their CT scans resulted in an estimate that the axial column stiffness would allow mobility of 3.5 degrees at the lowest, using the most conservative parameters for one of the fish and 8.75 degrees maximum, using the least conservative parameters for the other fish, comfortably surrounding the range of actual observed elevation *in vivo*. So, we found this to be a good sign for the validity of the method of estimation. Please see the first response to Referee #1’s comments below for more detail on the process.

For the remainder of your comments, I have greyed out the original text and offered my responses in purple below. The line numbers jump around between the tracked changes and clean versions of the manuscript as well as when viewing the document on a Mac, so I have tried to refer to the clean version when mentioning positions in the point-by-point responses and hope that they are findable for you all. Thank you all for your care and attention!

Best wishes,

Dana E. Korneisel

on behalf of the team

Referees' comments:

Referee #1 (Remarks to the Author):

Summary of key results
This study proposes that early/stem tetrapods had a notochord-dominated axial column, whose flexibility is determined by the material properties of soft tissues, rather than the mobility of bony intervertebral joints. Because notochord-dominated axial columns are relatively rare in living vertebrates, it has been hard to accurately assess the mobility of these early necks. Specifically, did the head-shoulder decoupling that gave rise to the neck increase mobility of the head relative to the trunk? This study tests the hypothesis that head mobility was the functional driver for head-shoulder separation.

This study measured stiffness of the axial column in lake sturgeon, which also have a notochord-dominated axial column. They used this stiffness, along with the dimensions (second moment of area, length) of the neck in 6 early/stem tetrapods to estimate maximum neck mobility in these extinct species. The results showed that, unless the neck was relatively long, neck mobility was low: <10 (usually <5) degrees of dorsal or lateral bending – even in species with a de-coupled head and shoulders.
The authors conclude that independent head mobility (including for feeding) was not a functional driver for separation of the head and neck. Instead, head-shoulder decoupling was likely driven by locomotion functions, and neck mobility primarily increased through the evolution of longer necks and mobile, bony intervertebral joints.

Originality and significance
I want to specifically applaud the authors for:
1) The use of sturgeon as a novel but appropriate model for early/stem tetrapods. It is particularly well-suited as one of the few living vertebrates whose axial column is dominated by notochord rather than bony joints.
2) Measuring empirical, mechanical data from living species to test hypotheses and inform predictions about extinct species.
This topic—the origin of the neck and its function, mobility in early/stem tetrapods—is widely interesting (as demonstrated by the large number of papers in this area). However relatively few apply quantitative, mechanical analysis of living analogues, so this subject is compelling and noteworthy to many people from a range of disciplines, e.g., biomechanics, evolution, palaeontology, ecology.

Validity and conclusions
1. The modelling used here, while logical, has not been validated on extant species. The conclusions of this study rely on knowing: how well do measurements of axial column material properties and dimensions predict the maximum potential (or even better, in vivo) bending of the neck or similar region of the spine? This could be done relatively easily by measuring bending and torsion within the same species, the lake sturgeon. It would be even more robust to validate it across multiple species (see also next point). Without this validation, it is difficult to assess the validity and degree of uncertainty in the bending estimated for extinct species.

Glad that the logic of our model followed for you, it is easy to lose the forest for the trees while developing a study like this! As to validating the method on extant species, that is a fantastic idea. We reached out to authors of published studies on lungfish and sturgeon (both seem good options for validation, see a bit of discussion on this in the next point) biomechanics to check our model against their measured results.

Samantha Gartner of Gartner et al, (2022, *Open Biology*) responded and was able to provide us with the original videos from her study from which we were able to measure the length of column involved in the head elevations they reported in their supplementary data and CT scans of the two lungfish she used from which we took axial column base and height measurements as well as a body cross section area measurement using Materialise Mimics. We have added to the acknowledgements to thank her for making these data available. Using these data, we put together the necessary calculations (in the attached excel file as the third

sheet along with the DIY ‘choose your own extinct taxon’ sheet and the 10 cm column scaled calculations sheet) to estimate extension using our method for her two fish. Gartner had measured neurocranium elevation peaking just under 5 degrees during feeding strikes in two specimens of *Protopterus annectens*. Our estimates indicate that a maximum extension would be 3.5 to 5.25 degrees (the lower estimate being for 200 kPa muscle pressure and the upper for 300 kPa muscle pressure) for the first fish. For the second fish, we could not be certain of the scaling on the scan as there was only a scan sheet with resolution noted for one of the fish. Assuming it was the same scan protocol as for fish 1, the second fish was quite similar in proportions overall to the first, it had a slightly smaller axial column but was larger in cross section overall, perhaps due to preservation (the first was scanned folded in half, with a more oval profile and possible lateral compression, the second was scanned straight and was quite round), the estimate was 5.25-8.75 degrees with the same reasoning for the two estimates.

Given these estimates, the habitual motion of feeding strikes *in vivo* may use close to the total possible range of motion for the axial column in isolation at its maximum observed extension (elevation measured by Gartner et al. ranged from around 2 to 5 degrees). There are a few other factors to consider here. First, we would expect these fish to have some additional stiffness added by their extensive mineralized dermal scales. Second, the actual effective muscle area with that can input force for head elevation might be greater. Effective muscle cross sectional area can be increased by angling of muscle fibers, but this is of course also a factor in the fossil taxa. I have added an explanation of this validation process to the extended data methods section on measurement.

We would have liked to also validate using sturgeon as a second point but were not able to get a response from researchers who may have relevant swimming and C-start data for fish of a meaningful size. That which we could find was a dissertation that appears not to have resulted in any peer reviewed publications and it used very young fish. The problem with using sturgeon that are near-hatch for this kind of validation is that their osteoderms don’t grow allometrically with their body size. Instead, they start out huge relative to the whole body so that hatchlings are strongly armored and not terribly mobile. This was pointed out to me first by the fishermen who provided my earliest sturgeon. They also noted that adult (about 20-30 year old fish over a meter in length, their growth slows significantly after a few years but they keep growing throughout their long lives, which are fished for smoking flesh and for caviar) sturgeon can turn their whole body such that they can practically touch their nose to their tail-tip. This was observed by the fishers in sturgeon that had gone into shallow water as an escape maneuver. We don’t have live fish available for swim tests.

I have added a short paragraph describing the methods for this validation to the end of the main methods section.

2. The conclusions also rely on notochord/soft-tissue properties being similar across actinopterygian and sarcopterygian fishes. Is there evidence that lake sturgeon are representative across this range? I was somewhat surprised this study did not use lungfish, which also have a notochord-dominated axial column, but are more closely related to early/stem tetrapods. If the study were repeated on another extant species, would the conclusions change? This could be addressed by measuring the material properties of the axial column in one or more additional species. Or explaining why A) there are good reasons

to expect these properties are similar across species, or B) no other extant species could be tested or is as appropriate a model for early/stem tetrapods.

"Notochord/soft-tissue properties [are] similar across actinopterygian and sarcopterygian fishes": Schmitz (1998a, *Journal of Morphology* 236:75–104 & 1998b, same issue pages 105-116) compared the structure of the notochord in sturgeon (*A. brevirostrum*) and lungfish (*Protopterus annectens*) histologically and with immunohistochemically and found that they were overwhelmingly similar at a cellular level, with the main difference being in the presence of endoplasmic reticulum in cells in a particular layer of the notochordal sheath in sturgeon that was not present in the sheath of lungfish.

B) no other extant species ~~could be tested or~~ is as appropriate a model for early/stem tetrapods: A number of researchers have looked into differences in the notochord between extant taxa using dissection and histology. The overall trend in differences between notochords is not phylogenetic but related to the degree of enclosure of the notochord by surrounding arches (cartilaginous or ossified), such that the more arch material surrounds the notochord the less thick the fibrous sheath of the notochord itself is. In cyclostomes, there is no arch material and the fibrous sheath is very thick (Long Jr. *et al.*, 2002, in main MS references), in the lungfish, which have small to very small central cartilages, the fibrous sheath is quite thick, up to about 2/3 of the diameter of the whole notochord (*Protopterus*, Goodrich, 1930, "Studies on the Structure and Development of Vertebrates" ch. 1 figure 10), in sturgeon the fibrous sheath makes up up to about 2/5 of the diameter of the whole notochord (our own observations on *Acipenser fulvescens*, including in Fig. 1D; *A. sturio*, Goodrich, 1930, ch. 1 figure 34) and in developing trout with extensive vertebral arches and centra forming, the sheath is much thinner, perhaps 1/6 of the total diameter (*Salmo*, Goodrich, 1930, ch. 1 figure 11).

Since the notochord is the same at the cellular level in sarcopterygians and actinopterygians, the important factor here is likely the sheath thickness. We know from Long Jr. *et al.* (2002) that the sheath contributes most to notochordal stiffness, and the vertebrae of stem and early tetrapods are more similar to sturgeon vertebrae than to the more reduced vertebrae of lungfish, so the notochord proportions were probably closer to sturgeon as well.

Could lungfish be tested? Perhaps, but I would caution against using them as a model for stem tetrapods. At the time of this study, the only lungfish we had access to were museum specimens stored in ethanol, which are dehydrated and stiff, making them unsuitable for this kind of biomechanical testing. I expect that most anyone interested in replicating this study would run into the same issue, so for practical reasons as well as biological reasons (similarity to taxa of interest, addressed above) I don't think they would have been/are a suitable model. It would be interesting for a future study to look into modern lungfish as a model for extinct lungfish (though some of these have more extensive centra, so I think that sturgeon would still be more appropriate for those taxa) or for coelacanths, which also have highly reduced centra. We can, though, and have used lungfish as a validation point for the method, as you pointed out in point one, above. You can find the results of that validation in the response to the previous point.

So, "If the study were repeated on another extant species, would the conclusions change?" I expect that the notochordal stiffness in lungfish would be higher than in sturgeon due to the thicker fibrous sheath. In sturgeon, most of the stiffness of the axial column comes from the

arches being in place and the notochord alone is much more flexible, but this may be a very different relationship in lungfish because their vertebral components are highly reduced compared to tetrapodomorphs.

Data and Methodology

For the stem and early tetrapods, how many and which vertebrae were measured for second moment of area calculations? From Supplemental Figure 7, at least vertebral height—if not width—varies along the cervical column. So, were dimensions taken from a single vertebra or averaged from multiple vertebrae averaged? I couldn't find this information, which would be essential to reproduce the results.

For *Tiktaalik roseae*, the measurements were taken from the vertebra shown in Extended Data figure 6, which is the anteriormost ossified vertebra in the specimen, identified as V4 by Stewart *et al.* (2024). I have added that information to the caption of Extended Data figure 6.

For the others, the measurements are from the single reconstructed vertebra shown for each in Fig. S7 and these are all made from the best reconstructable (good 3D preservation) vertebra available in the column for each taxon. The morphology of the vertebra for *Acanthostega* is equivalent to the posteriormost cervical vertebra. *Whatcheeria*'s is reconstructed from multiple isolated vertebral components, modeled on the posterior cervicals. *Greererpeton*'s is "presacral 5" (Godfrey, 1989, figure 7), which I prefer to term V5 for consistency. *Trimerorhachis*' is the only one that is from more posterior vertebrae. It is reconstructed from neural arches from posterior trunk vertebrae and isolated centra. We have added the vertebral number or description of source for each to the caption in Extended Data figure 7!

Clarity

The paper would benefit greatly from clearly stating how the location and mobility of the head, neck, and body are being defined. Specifically, the following terms:
'total head motion' (what does this include?)
'rotational head turning' (rotation in which axis?)
'neck'
'head mobility'
'neck bending' vs 'trunk bending'
For example, if head mobility is defined as motion relative to the shoulder, then the separation of the head and shoulder girdle must always increase the head's mobility. Similarly, the distinction between 'neck' and 'trunk' is somewhat ambiguous in a study that includes at least two species with no clearly defined cervical region.

'Total head Motion': This was meant to refer to movement of the whole head relative to the trunk, distinguishing that from motions in the head of fishes including tetrapodomorphs that are more independent such as flexion at an intracranial joint or jaw motions. I have tried a lot of different options here to clarify and most of them get really wordy in a way that, I expect, is more confusing. So, I have opted to insert a parenthetical "(as opposed to intracranial motion)" after the term "total head motion" (currently line 39-40) and hope this suits.

'Rotational head turning': Referee 3 made some suggestions for standardized terminology here, and we have followed their suggestions. So, instead of saying 'rotational head turning'

is now just ‘rotation’ as is ‘torsion’, though the term torsion is still used in reference to the type of testing performed on the universal testing machine, the torsion drive, and the torsion constant.

‘Neck bending’, and ‘trunk bending’: We also followed Referee 3’s suggestions here. Lateral bending is now ‘lateral flexion’. This has been changed where we originally used the term ‘lateral neck bending’ and where ‘trunk bending’ has been replaced by “additional lateral flexion in the trunk region” in line 267. Dorsal bending is now referred to as ‘extension’ with the first instance clarified with an “(i.e., head elevation)”

Additional changes to conform with this – Line 24-25 now reads “lateral flexion, extension, and rotation”

Line 28-29 reads “Crucially, mobility did not shift…” as the list of types of mobility made the abstract too long with other edits incorporated, and this cut seemed suitable to bring it back to the correct length

Line 76-77 reads “…measured lateral flexion and dorsoventral extension stiffness and torsional stiffness (i.e., for rotation).”

Line 101 starts “In lateral flexion…”

Line 106 ‘lateral bending’ replaced with “lateral flexion”

Line 118 “*Bending*” replaced with “*Lateral Flexion and Extension (head elevation)”*

Line 126-128 “Lateral and dorsal bending” replaced with “lateral flexion and extension”

Line 132-134 now reads “With the exception of *Whatcheeria*, stem tetrapods generally were not capable of lateral flexion of their necks greater than 8.5° or extension greater than than 3°.” As a result of multiple changes

Similar changes also made in lines 137, 147, 152, 180, 184, 193, 219, 234, 262, 267, Figure 2 caption.

In the Methods section, bending still refers to the testing, and I have added a clarifying parentheticals to line 502 “(equivalent to lateral flexion)” and 505 “(equivalent to extension)” to tie this to the main text. Where referring to the estimates, changes were made to suit the new terminology as well as in the Extended Data table titles in the extended data file.

Finally, went through the extended data file in the same way to make relevant changes. Kept the terms torsion and bending when referring to testing on the UTM or material properties where those terms apply, so the changes were largely to supplemental table or figure captions.

‘head mobility’: There is one line on *Eusthenopteron* regarding it not having a neck region but only a few slightly differentiated anterior vertebrae (line 189-191), but clearly this wasn’t sufficient to make clear why it is included or what the ‘estimates’ mean for this taxon. We thought it was important to include an out-group taxon in this study to give context to the rest of the estimates and to make it possible to actually compare across the skull-shoulder decoupling event. I have added “…from which we took a length of axial column on which to model theoretical mobility were the region to be free” to the end of that sentence in order to

clarify Eusthenopteron's place in the study. To line 188, we added "but see below" after Eusthenopteron in the list referring to neck lengths to direct the reader to the next sentence for clarification. To Figure 2's caption, we added (or analogous region) after 'neck region' to further clarify.

We intend for this term to refer to mobility relative to the trunk rather than the shoulder itself, though the pectoral limb is biomechanically important as an anchor point in any of the possible functions that follow from this (e.g., lateral snapping as imagined with the forelimb planted). I think that it is most appropriate to clarify this term in the first main text instance of its use, Line 45-46, rather than in the abstract, so I have added the following to the relevant sentence "…such that the head could move more through added flexion, extension, or rotation capability of the neck region." This should both be in keeping with the terminology chosen through the other edits made in response to this comment (and similar comment by Referee 3) and clarify our meaning of this term. We have also checked for instances where the term 'head mobility' was used and changed it to 'neck mobility" to keep this consistent throughout (e.g., lines 230, 241) and disentangle the meaning from any internal cranial kinesis.

'Neck': This one was also a topic of comment by Referee 3, who expanded on the differences in the use of this term among vertebrates. So, I am responding to your request here as well as their added comments (see below for those).

We have made sure to say "tetrapod neck" to disentangle this use from other definitions. Edits for this are on lines 23, 24, 30, 110, 155, 222, 239, and 258. In many cases, terms like "neck region of stem tetrapods" or "neck of *Taxon*" were already in use and have been kept meaning the same thing to avoid redundancy that would be introduced by saying 'tetrapod neck' in these instances.

Line 40-42 is the best opportunity to clarify this in the main text, so I have edited it to the following to clarify that we are discussing the early appearance of a tetrapod neck rather than a neck by any other definition: "…when the skull and shoulder girdle separated, giving rise to a distinct neck region unique to tetrapods".

It would also be useful to explicitly state that you are not studying the neck as a joint-based system (as most other studies do). Instead, the assumption is that the neck functions as a single structure in bending, based on constant material properties.

Very true! The term you use here 'joint-based system' solves a big terminology block that I have been facing in the introduction trying to describe how these axial columns differ from most axial columns today. I have re-written a sentence in the main (lines 56-58) to use this term, which I think answers your request for an explicit statement and makes the difference clearer at the same time: "These differences in anatomy mean that the stem tetrapod axial column is not a joint-based system as the crown tetrapod axial column is, making the 'soft' axial columns of stem tetrapods challenging to model."

Suggested Improvements

It would be useful to include a measure of bending per joint or unit of length to show how neck shape—not just length—impacts mobility. It is not greatly surprising that species with longer necks are predicted to have greater head mobility.

Answering this one was a kind of fun theoretical. Bending for a set length is a good idea to compare the shape impacts on mobility, and could inform the portion of the discussion on how shape impacts bending.

The trouble with this comparison is that the size of the animal plays into other factors in the calculation as well. It is tied to the accepted forces because this is determined by maximum cross sectional muscle area, so I think a set force would also have to be chosen for this comparison (otherwise: bigger animal = larger muscle cross sectional area and resulting larger force accepted). The animal size is also part of the I calculation in that the columns don't just have different proportions but are larger. So, the proportions would need to be scaled to a given axial column height or width as would the notochord size. I have chosen to scale all others to the Base width of *Eusthenopteron* for the sake of looking into this because it is listed first in my spreadsheet and the widths were more similar to one another than the heights to start out with.

I am going to try to attach the excel sheet to this response with the full calculation in it, but here are how the bending estimates shake out when the axial columns are scaled to the same size by base (i.e., width), bending is calculated across 10 cm, and the angles are fit to result from the same amount of force applied to each theoretical column:

| **Taxon** | **Lateral Flexion (°)** | **Doral Extension (°)** | **Torque (Nm)** |
|---|---|---|---|
| **Whole Column** (Force for lateral flexion = 1.44E+03 N, Force or extension = 4.13E+03 N, Torque at 5° rotation | | | |
| *Eusthenopteron foordi* | 10 | 10 | 116 |
| *Tiktaalik roseae* | 10.9 | 13.05 | 93.8 |
| *Acanthostega gunnari* | 7.85 | 4.88 | 210.5 |
| *Whatcheeria deltae* | 10.25 | 10.75 | 109.4 |
| *Greererpeton burkemorani* | 13.2 | 22.6 | 61.1 |
| *Trimerorhachis insignis* | 0.54 | 0.526 | 636.9 |
| **Notochord** (Force for lateral flexion = 5.24E+01 N, Force or extension = 1.06E+02 N, Torque at 5° rotation | | | |
| *Eusthenopteron foordi* | 10 | 10 | 0.000804035 |
| *Tiktaalik roseae* | 28.25 | 28.2 | 0.000264233 |
| *Acanthostega gunnari* | 5.91 | 5.9 | 0.001371649 |
| *Whatcheeria deltae* | 77.7 | 77.6 | 3.09264E-05 |
| *Greererpeton burkemorani* | 53.7 | 53.5 | 0.000104203 |
| *Trimerorhachis insignis* | 6.53 | 6.5 | 0.001239542 |

I have made the first part of this table on the whole column into Extended Data Table 13 with the following header added to the list in the main text document: "**Extended Data Table 13.** The influence of axial column proportions on mobility."

So, what does this mean for proportions influence on bending and rotation?

I don't think the notochord section really does much for us here, so I have not included it in the supplemental table. If you look at all of the cross-sections, we are just seeing a 'big notochords are stiffer small notochords are less stiff' relationship reflected here. Not really interesting or informative.

The whole column part is interesting, though! The proportions of *Tiktaalik*, *Whatcheeria*, and *Greererpeton* result in more bendiness than *Eusthenopteron* while the proportions of *Acanthostega* and *Trimerorhachis* result in a stiffer column. The pattern is the same for both bending directions but it is much more pronounced in extension! This actually follows really well from the discussion we have in place on proportions.

The standouts here are *Greererpeton*, *Acanthostega*, and *Trimerorhachis*, and these certainly add additional perspective to consider in the discussion! *Greererpeton*'s short and broad profile is favorable for extension whereas *Acanthostega*'s tall and narrow profile is restrictive. The thing that really stands out here that wasn't nearly as clear in the estimates, though, is that *Trimerorachis*' rectangular profile is very impactful! I lost that in the main estimation process because it also has such a short neck, but the rectangular shape is also a major factor. I have made some adjustments and an addition starting at line 204 to the discussion section on proportions of the column referring to the new Table S13 as a result of this new perspective.

Please state what—if any—impact freezing and thawing is known to have on the material properties of soft tissues like the notochord.

This has been a subject of hot debate (wordplay not intended but kept in once noticed), especially in the surgical literature! It was also a huge concern of mine going into experimental design. I was reassured by the literature on this topic that a single freezing event in a typical -20 degrees Celsius freezer would have minimal effect on the material properties of soft tissues. A single freezing event has a minor effect on bone tissue, smaller than the differences present through natural variation in the tissue (Kaye et al. *Open Bone* 4.1: 14-19) and on articular cartilage (Changoor et al. *Journal of Biomechanical Engineering* 132(6): 064502). Other literature (see next) has suggested that freezing temperature does not matter, but none has suggested that -20 is worse than -80 that I have seen.

More recent work by Dhiman et al. (2025, *Journal of Biomechanics* 188: 112779) found that the material properties of intervertebral disc material, perhaps the most similar tissue to notochord that is tested for storage degradation in the literature, was only influenced by freezing in degenerated discs, not healthy ones.

There is some indication that certain tissue types like tendon are not highly affected by freezing temp or by number of freezes and thaws (e.g., Blaker et al. 2024 *Journal of Orthopaedic Research* 42.6: 1180-1189.) but I worked with some large sturgeon material in developing the method for this study and noticed a considerable qualitiative difference in the tissues across multiple freeze-thaw cycles so decided that a single freeze and thaw would be the limit for this study.

So, in response to this comment, I have added a briefer version of this information into the extended data under "Specimen Preparation / Storage".

Please include how many early/stem tetrapods had notochord-dominated axial columns (and are therefore well-represented by this study)?

To address your exact question, this method to look into neck mobility applies to elpistostegalians and all early tetrapods more closely related to the tetrapod crown group than to elpistostegalians (e.g., *Elginerpeton* if we were ever to get any postcrania, Ichthyostegids,

Colosteids, Whacheeriids, Baphetids, and everyone in between, just excluding 'Embolomeres' if you consider them to be stem tetrapods). Additionally, this method can be used in the tetrapod crown group including in any temnospondyl until the notochordal canal is highly restricted by centra, though this would also need to consider the elaboration of zygopophyseal articulations. I do not know of any amniotes for which it would be applicable due to their high degree of notochordal restriction.

Though our study presented here focuses on the cervical regions specifically, this method could be applied to any part of the axial column, so it is applicable both to stem and early tetrapods as well as to a wide range on non-tetrapod-line fishes for addressing different questions. If you would like to expand and do a project on part of the column outside of the neck region, it is applicable far outside of stem and early tetrapods. It could be used to estimate a particular region's bending or total body bending in Osteolepiforms, for example, which also have notochordal axial columns. This kind of approach could be applied also to other sarcopterygians so long as proportions of the axial column could be measured. It could similarly be applied to jawless fish, some placoderms, many Paleozoic and Mesozoic actinopterygian clades besides acipenseriforms, and some chondrichthyans *if the relevant axial column measurements are possible*. That means that we need to be able to provide a reasonable estimate of the axial column shape in cross section and its size (base and height if triangular) if we wanted to approach additional studies on one of these clades. For any one of these, though, I would *take a close look at the size and coverage of the vertebral arches* relative to the notochordal canal and strongly advise consideration of the cyclostome literature as well in any cases where the vertebral arches are small/reduced.

It could also be used to estimate mobility in extant animals that cannot be directly studied due to endangered status or similar (I am thinking paddlefish) if there was reason to want this.

The fused occipital vertebrae of sturgeon are mentioned, but not discussed or shown in detail. Please explain if/how these would be expected to impact mobility and justify why this doesn't impact the applicability of sturgeon as a model for stem and early tetrapods.

I have added a note on the occipital vertebrae to line 589 (original submission line 588, shifted with edits incorporated) to note that the vertebrae posterior to the occipital vertebrae are what I'm considering the most comparable region of the sturgeon axial column to the neck of stem/early tetrapods. I expect that their mechanical impact is to move the effective end of the skull backwards, so rather than ending at the occipital condyle the skull unit in a sturgeon includes also the occipital vertebrae. This is why the first testing position for bending is back at the level of the 12$^{th}$ rib (behind the 4-5 occipital vertebrae) and why there are specific gauge measurements for every anterior torsion test to measure to the effective back of the skull rather than to the neurocranium-first occipital vertebra dividing line.

Please explain and justify why the modelling approach in this study doesn't take into account any variation in the morphology of ossified elements of the cervical or anterior axial column.

I could see this question referring to two different things, either variation in morphology from the anterior to the posterior cervical vertebrae in one animal or to the variation in vertebral morphology between taxa.

If you meant the former, then this response is tied to the response to your earlier question about which vertebra was used for the cross-sectional measurements. There is usually a slight grade in size or proportion of the vertebrae across a portion of the column. These are pretty minor in the animals included in this study, but we aimed to choose a mid-point in the column so the difference from our measurements to what would be measured on either extreme end of the column section would be minimized.

If it is the latter, then I think this response is necessarily entangled with the response to the ‘boney stops’ questions posed by Referee #4 and your earlier comment on this not being a joint-based system. In fact, the reason we don’t take this variation into account is the same as the reason we needed to develop a new non-joint-based method to estimate mobility in the first place. The variations in the vertebral components probably do have an impact, but not on the measurements that go into this method. They are not something that can be addressed by this method or by existing joint-based methods that rely on establishing stop points between the articulations of vertebrae as an outside limit of mobility between the vertebrae. I do not know of a method that would allow internal freedom of motion between components of each vertebra (e.g., one that would allow for the space between the intercentrum and pleurocentrum of the same vertebra to widen or narrow with bending). For the effect of different vertebral anatomies to be examined in a notochordal axial column, that kind of modeling would be necessary. I think that kind of model could theoretically be established, and I may even look into it in the future. I imagine that it would start with dissections of animals with multipartite vertebrae and manual manipulation to see if/how these vertebral components move relative to one another. It is interesting to think about, but that would be at least one if not a few other projects.

Referee #1 (Remarks on code availability):

The provided code on the sturgeon data in the CrshR repository was tested and returned results similar to those presented in the paper. The manual selection of break and change points presents a potential source of variability, especially as there are no instructions on what is being selected or why. Otherwise, the code meets the specified requirements for reproducibility. However, detailed annotations in the code or a thorough README file would clear up uncertainties; the current README provides almost no instructions.

CMD has added a README file to the github repository that explains the code (not the same as the demonstration text that I submitted with the initial submission which, you are absolutely correct, was very minimal and meant more to note that the code did not take much computing power to run rather than to explain its implementation). She has also gone through the code and added annotations to make use of it easier for non-coders.

We were not exactly sure what you meant by break and change points, but interpreted that to mean spots at which the next bit of code has to be started after one part of the script has run. CMD has added annotations specifically with an eye towards clarifying the order in which different parts of the script should be run to help with this.

Referee #2 (Remarks to the Author):

I co-reviewed this manuscript with one of the reviewers who provided the listed reports.

Referee #2 (Remarks on code availability):

I was able to use and run the code, however there is no README file with sufficient instructions provided for others without former knowledge of RStudio.

CMD has added a README file to the github repository that explains the code and annotations to the code itself to make it easier to use for non-coders.

Referee #3 (Remarks to the Author):

This manuscript entitled "Early tetrapod neck mobility modeled from an extant analogue" by Dana E. Korneisel and colleagues examined the mobility of the neck, which was newly evolved anatomical part between the head and shoulder in the stem tetrapods, based on the biomechanical properties of the notochord, and found that the neck mobilities were limited in early limbed tetrapods. For the past 20 years, our understanding of the transition from fish to tetrapods has made great progress, particularly in the morphological and functional changes from fins to limbs. On the other hand, our understanding of the function of the neck in the lineage leading to tetrapods has remained poor, and this study is the first to do so. The methodology of this study represents the best approach to quantitatively estimating neck mobility from the fossil data currently available and is highly convincing. This finding will be of interest to many scientists as it is important for understanding how tetrapods evolved from fish and how our body became established. Therefore, I believe this study is worth publishing from Nature, although there is room for improvement before publication.

Major comments.

(1) Background of this study: neck.
The morphological differences between necked crown tetrapods and neckless fishes should be better explained. In vertebrate morphology, the term "neck" has been used by various researchers to refer to structures found in various taxa. For example, the term "neck" is often used to describe the gap between the head and shoulder of placoderms and the other fishes. On the other hand, the "neck" in the human body is a region lacking internal organs (i.e., a coelom), and is distinct from that of amphibians. It should be called the "amniote neck" (see Hirasawa et al., 2016). In amphibians, the heart is located in the "neck." The "neck" used in this study is used to refer to the structure that constitutes the body plan of crown tetrapods, and should be called the "tetrapod neck." It would be better to explain how it evolved from the fish to the amniote conditions (this should be explained in the diagram, see Comment (5)). Hirasawa, T., S. Fujimoto, and S. Kuratani. 2016. Expansion of the neck reconstituted

the shoulder-diaphragm in amniote evolution. Development, Growth & Differentiation 58: 143–153.

For the part of this comment that is about the term ‘neck’, see the response to Referee 1’s request for clarification on this term under their ‘Clarity’ heading. I have taken note from your helpful input here in making changes described in that response. In short, I take your suggestion to say “tetrapod neck” throughout.

We agree that the differences between axial columns are best shown in a diagram rather than further explained in the text, and have removed the transparency from cartilaginous arches depicted in figure 1 to improve their visibility.

It's debatable whether the evolutionary process covered in this paper is appropriately called "head-shoulder decoupling." I believe the "decoupling" referred to here is functional, not developmental, but the authors don't even explain this. As the results of this study, neck mobility was shown to be quite limited, so I suppose that (functional) "decoupling" is not appropriate. I think the term "separation," used elsewhere in this paper, is a more appropriate description of this evolution.

I see where you’re coming from, here, it was absolutely intended to be about potential impact on function rather than a developmental term. I am happy to change up the language to avoid potential confusion, and have changed decoupled/decoupling to separated/separation throughout the text.

(2) Background of this study: notochord.
It should be more clearly explained that the evolutionary process focused by this study is the stage before the evolutionary establishment of the vertebral column. As mentioned in comment (5), the evolution of the axial skeleton can be better explained by illustrating it in a Figure.

I have looked for a good place to include a more explicit comment on this, and settled on the second paragraph of the main text, where this topic is already being discussed. On lines 50-52, I have changed the start of the sentence to read “This condition predates the evolution of the vertebral column that characterizes most adult crown tetrapods in which the notochord is largely replaced with bony centra, …”.

Regarding the idea that the axial skeleton supported by the notochord is more flexible, it may be helpful to explain this by using the example of the dragonfish lineage, a teleost lineage in which the vertebral elements in the neck region were secondarily reduced and the skeleton was supported by the notochord (Schnell et al., 2010). In this lineage, the secondarily acquisition of the notochord-supported axial skeleton increased the mobility of the neck region.

Schnell, N. K., R. Britz, and G. D. Johnson. 2010. New insights into the complex structure and ontogeny of the occipito-vertebral gap in barbeled dragonfishes (Stomiidae, Teleostei). Journal of Morphology 271:1006–1022.

This was a really interesting read; I didn't know about dragonfish necks before! Having seen a lot of work on injury to notochords, I am shocked that they can achieve such extreme bending without causing lesions in the notochordal tissue. It would be really interesting to see some histology of this notochord, I expect it may have a very thick external sheath like that of hagfish. The dragonfish necks (especially the most extreme instances) go beyond what is comparable to early tetrapods with vertebral reduction. Most of them (like the various *Chauliodus sp.*s) have such a reduction that they look more like lungfish columns. I think this topic fit best under the discussion of notochordal stiffness in the section "The sturgeon axial column as a model for the early tetrapod neck", as an additional point noting that the notochord alone is not always so stiff as in hagfish. So, I have added a note on this in line 88-91.

(3) Explanation of how to calculate neck lengths.
There is a lack of explanation for how the number of vertebrae corresponding to the neck region was determined, making it difficult to determine whether this is appropriate. For example, while the number of the neck vertebrae of *Whatcheeria* seems to be based on the detailed description by Otto et al. (2021), rather than simply accepting the number of "cervical vertebrae" given there, the study should provide a reproducible explanation of the criteria used to determine the number of neck bones (e.g., the presence or absence of developed ribs). Regarding *Whatcheeria*, ribs are also present in the areas considered to be vertebrae in the neck, so the neck length estimate may need to be left to a certain extent.

You are right to notice that I only explained the measurement method from the 3D model of *Tiktaalik* not those from the reconstructions. I have added the following to the "Measurements" section of the extended data to clarify the placement of our measurement plane: "Measurements from all other taxa (Extended Data Table 5) were also taken from the anteriormost point of the first intercentrum to the posterior edge of the last cervical (or non-cervical anterior column region, as in Eusthenopteron) vertebra centrum, measured on reconstructions (Extended Data Figure 7). Except for in *Eusthenopteron*, the definition of the cervical vertebral region followed that of the source material (citations in Extended Data Figure 7)."

As I am sure you know, the definitions used for cervical vertebrae are wildly variable, largely because of body plan variations in tetrapods (e.g., limbs vs no limbs, cervical ribs vs no cervical ribs) but also due to preservation biases of individual fossil specimens. Cervical ribs are actually a very common phenomenon. It is only in mammals (and sort of Gondwanatherians) that the lack of ribs is used as a defining feature of cervical vertebrae, so that is not an applicable criterion to these animals.

I (DEK) actually have a work in progress looking into this wild variation in definition. Apologies for referring to unpublished data, but by my count so far there are 29 different skeletal criteria given as definitive of cervical vertebrae (not including the 30$^{th}$ which is no criteria given) in published anatomical descriptions. For this reason, we made no attempt to force all of these taxa to conform to one definition of the cervical region and instead use the definitions from the source material with citation given. The one exception to this is *Whatcheeria*, where what DEK identified as an extraneous pleurocentrum at the anterior end of the column in the original reconstruction by Otoo et al. (2021) (see Korneisel & Maddin,

2025 *Biological Reviews* figure 9E and figure 9 caption) has been removed.

(4) Explanation of the relevance of choosing the sturgeon as a model.
In my opinion, there is no problem with using the sturgeon as a model for vertebrates with an axial skeleton supported by the notochord. However, the explanation for the choice of this taxon should be more detailed. It should also be explained whether the shared features seen in stem tetrapods and the sturgeon represent an ancestral state or a secondary related state (see comment (14)). It should also be specified what the "additional connective tissues" are (ligaments? fascia?) and whether they were also present in stem tetrapods.

I have changed the wording per your suggestion in comment #14 and think that it makes the relationship very clear. I have also added a clause to line 65, "The notochord-dominated axial column of sturgeon, *a secondary adaptation in their lineage*, is similar in its anatomical configuration…" to state explicitly that the notochordal axial column is not a maintained ancestral feature in acipenseriforms.

For 'additional connective tissues', this is referring to the tissue connecting the arches to one another and to the notochord. These are multiple, it is not referring to a specific ligament or similar. So, I have reworded to the following: "This type of column is a composite of soft tissue (notochord), hard tissue (cartilage or bone arches), and the additional connective tissues that unite them. All contribute to these material properties." There was surely tissue connecting the vertebral components in stem tetrapods, but precisely what the makeup of this was is not something we can know.

(5) Main Figures
I believe a phylogenetic tree showing the evolution of notochords and the phylogenetic relationship of stem tetrapods should be included as a figure in the main text. The phylogenetic position of *Trimerorhachis* (temnospondyl), which is being analyzed in this study, should be explained properly, otherwise it will lead to misunderstandings. The branching of the lineage leading to the amniotes and the lineage leading to the lissamphibians, along with the phylogenetic position of the acquisition of the intervertebral joint, should be presented. The process of the head-shoulder separation should also be explained using lateral views of the skeleton.

Fig. 1 in the current manuscript includes illustrations of *Eusthenopteron*, *Acanthostega*, and *Acipenser*, but this is not informative, as the essential axial skeletal morphology is barely visible. Body silhouettes are unnecessary here. Rather, the skeletal elements (notochord and bones) should be clearly and in detail illustrated. Something like Supplementary Figure 7, but with the addition of notochord etc.

I disagree that the silhouettes are unnecessary. This is being presented for a very broad audience, and presentation of the skeletal elements divorced from their body context would probably obscure the relevance for most readers outside of our field. The figures have been prepared at high resolution and will be viewed by most readers digitally, so zooming in on the vertebrae will be possible for those who are interested.

There are larger considerations at play in the reorganization of the figures, but I have kept these comments in consideration while making these changes.

The way the data is presented in Fig. 2 is a little difficult to understand. It would be better to make the comparison of estimated range of motion easier to understand than the silhouettes of the animals' bodies. A previous paper dealing with similar estimations, Pierce et al. (2012), is a good reference. (Incidentally, Pierce et al. (2012) also shares with this paper the use of a phylogenetically independent animal as a model for joint range of motion.) I believe this type of graphical comparison is better. Also, it would be better to compare both notochord-only estimates and whole axial column estimates on the same graph.

The figure caption already directs the reader to Extended Data Table 5 for those who would like to see this presented in a numerical format. A bar graph approach would add almost nothing to what is available in the table, whereas illustration on the silhouettes actually shows what these numbers could mean for the animals. The Pierce figures are also nice, but I think their implications are less intuitive than in our presentation.

A previous version of this figure did illustrate the notochord estimates as well (you can see it included here below). Since the whole column is so stiff, though, the notochord-based estimates really aren't applicable and we would not want to imply that we think they represent a likely range of motion. But, you can see one comparison of these limits shown in Extended Data figure 9

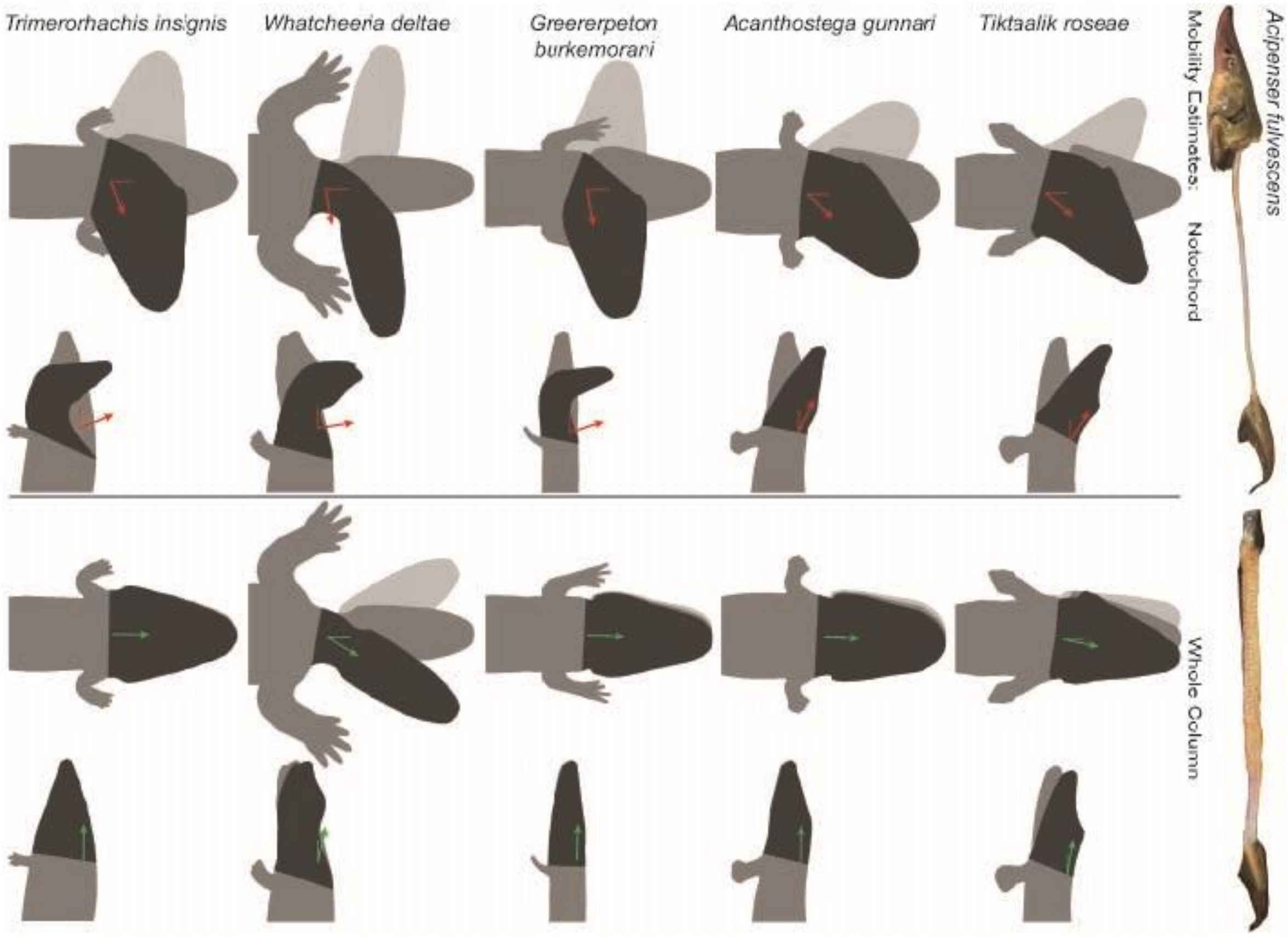


The phylogenetic tree in Fig. 2 in the current manuscript is very difficult to understand, so it

would be better to show the phylogenetic relationships in Fig. 1 separately and use Fig. 2 to compare the estimated values graphically.

I have reformatted figure 1 to include phylogenetic relationships and some changes to the axial column structure plus related landmark shifts (appearance of limbs, loss of gills) bringing together a few of your comments and comments from other reviewers. In order to accommodate this tree, I did have to decrease the size of the drawings, which is unfortunate given that one of your other comments was about how visible the vertebrae in these were, but I managed to mitigate this size reduction by changing the figure to a full page rather than 1 column width.

(6) Significant Figures of the Data
Throughout the paper, significant figures appear to be handled inappropriately. For example, it seems nearly impossible to measure fossil skeletons of the size under consideration with an accuracy of 0.01 mm, and such precision is not necessary, considering postmortem deformation of the fossils. Furthermore, while the actual measurements of material properties are given with very precise values, it is questionable whether these actually correspond to the accuracy of the measuring equipment.

You are right, these measurements were all digital and this was not considered with the care that it would have been with a caliper or similar. I appreciate your bringing this up for further consideration. None of these measurements are directly on physical objects. The measurements on *Tiktaalik* are limited by the pixel size from the scan represented in a 3D model and follow the digital measurement directly from Blender. The other figures given in the tables here, though, are all from measurements taken on images of reconstructions, measured to the pixel and then converted to mms.

I have given some thought to how this maps back onto non-digital methods and decided that, since there is no extrapolation beyond whole pixels involved here and the source of the measurements is clear in the extended data, additional rounding steps would not improve accuracy. I believe the measurements could easily be repeated by any interested party in their current state.

(7) Anatomical direction terminology.
In vertebrate anatomy, directional terms are defined to describe body movement: rotation of the head is called "rotation" (or "axial rotation"), sideways bending is called "lateral flexion," and dorsal bending is called "extension." "Extension" in this case could be called "head elevation," but "bending" and "torsion" are not common in vertebrate anatomy, so I believe it would be better to revise the terminology.

Thank you for these term suggestions. Referee 1 also commented on these terms, and I have referred to your suggestions here in how I edited to respond to their comments. You can find line-by-line changes listed in the response to their comment under the heading ‘clarity’ above.

Minor comments.

(8) Lines 22 and 26.
"co-ossified"
I don't understand why the authors say "co-ossified." It should simply be "ossified."

This is in reference to vertebrae in which each vertebral component (e.g., neural arch, intercentrum, pleurocentrum, supraneural where applicable) is fully ossified in the sense that the degree of ossification appears to represent the adult condition but components remain separate from one another within a single vertebra. I believe changing the phrasing on line 22 to "ossified" would incorrectly imply that the components should ossify together in these taxa. I believe that changing the phrasing on line 26 to "ossified" would imply that the vertebrae remain cartilaginous, which is not the case for most taxa examined herein. For these reasons, I have not made the suggested changes.

(9) Line 25.
"a soft-tissue dominated axial column is surprisingly rigid" should be changed into "a soft-tissue dominated axial column was surprisingly rigid"

Changed as suggested.

(10) Lines 27, 56, 128, 131, 203, 219, and 220.
"the capacity to move their heads"
Here, "capability" is more appropriate than "capacity."

Checked each use of the word "capacity" in the text and changed most of them as suggested. There were a few places that I think it was still the best fit. In two places, edited the sentences to remove the word but used different wording than suggested. The original Line 131 was changed more in response to your previous terminology suggestion.

(11) Lines 28–30.
This sentence reads as if more-crownward stem tetrapods evolved from the tristichopterids, but this is not the case. It should be rewritten to be phylogenetically accurate.

I can see what you mean here, I have edited as follows: "…with the head-shoulder decoupling that occurred between the origin of tristichopterids (represented by *Eusthenopteron*) and that of more crown-ward stem tetrapods." I think this lays out a sequence of events rather than implying a direct relationship.

(12) Lines 46.
"soft notochord" should be changed into "soft tissue notochord." At this point, data regarding hardness relative to other tissues are not cited. Thus, here the authors should just say "soft tissue" or "unmineralized."

Edited as suggested. Also checked the remaining manuscript for uses of the word "soft" and edited accordingly where necessary.

(13) Line 49
"crown tetrapods today"
Remove "today." It's redundant.

Edited as suggested.

(14) Line 57.
"phylogenetically distant but anatomically similar"
The phylogenetic distance is not an issue per se. What is important is whether they are of the same origin or independent. So, it should be rephrased as "phylogenetically independent but anatomically comparable."

I like this wording a lot, thank you! Changed to this.

(15) Lines 66, 67, 69, 129, and 147.
"early tetrapods" should be changed into "stem tetrapods." *Trimerorachis* should be excluded from this category.

I chose to use the term "early tetrapods" precisely because *Trimerorachis* is part of this study. I have made a compromise edit here that I hope will be acceptable – changing each of these occurrences to read "stem and early tetrapods".

(16) Line 90.
"in-tact"
Remove "-."

Done

(17) Line 95.
"posterior precloacal axial column." (Add "precloacal.")

This statement is applicable to the tail as well, so I have added the following: "…precloacal and tail regions of the…"

(18) Lines 99, 117, and 601.
The "m" in Young's modulus should be lowercase.

Changed here and, where necessary, in the extended data

(19) Line 104.
"identified" should be changed into "focused on."

Chose "selected" as an alternative here

(20) Line 109.
"is" should be changed into "was."

Changed as suggested.

(21) Lines 117–118.
The variables (E, I, and L) should be written in italics.

Done. Checked for other places where these would occur in the text and did not find other locations to make the same change. They occur in extended data tables as well and have

been changed there.

(22) Lines 120 and 611.
"the established pressures for vertebrate striated muscles"
"Established" sounds somewhat strange. "Known" is appropriate.

I can see this, have changed accordingly.

(23) Line 127.
"5.5–8.5°." These values correspond to *Tiktaalik* and *Eusthenopteron*, respectively, and should not be explained as a range.

Good point, the parenthetical now reads like this: "(8.5° degrees for the former and 5.5° for the latter laterally; Fig. 2)."

(24) Line 148.
"34.2–37.6%." Use a dash rather than a hyphen.

This strikes me as the same problem that you pointed out in the last comment, it shouldn't appear as a range, so I have changed to the following: "34.2% in *Acanthostega* and 37.6% in *Greererpeton*"

(25) Lines 146–148.
"Whatcheeria is an outlier in neck length among early [stem] tetrapods, as its neck length relative to skull length is more than double that of other stem tetrapods."
What is the rationale for discussing neck length compared to skull length, and not skull width?

Skull length strikes me as the more intuitive comparison to neck length because they are both measurements in the same axis. The rationale for using a comparative measurement is that *Whatcheeria* is the largest taxon in the study, so any comparison of measurements alone would be misleading.

(26) Lines 202, 230, 246, and 248.
"head mobility" should be changed into "neck mobility."

Also noted in a response to Referee #1 on clarity of terms, all instances have been changed as suggested

(27) Line 204.
"peripheral" should be changed into "superficial."

Changed as suggested

(28) Lines 206–209.
This sentence should be simply rephrased as:
"Additional constraints on neck mobility could also come from the internal organs that would not allow extension or compression created by neck motion, such as the gill apparatus."

Based on the loss of postbranchial lamina (see Otto et al. (2021)), evolutionary changes in the gill apparatus should be included in the phylogenetic tree shown in [new] Fig. 1.

Changed mostly as suggested. Kept “tolerate” rather than “allow” as I think it is more accurate.

I had a conversation with BKAO about what to do with the idea of including gills in the tree along with the larger reformat of Figure 1. The evolutionary history with the loss of the PBL and any possible independent losses or regains is not clear because it is present in *Greerpreton* but absent in *Whatcheeria*, and this makes putting it on this simplified tree problematic. As we haven’t done any new analysis on the gill apparatus in this project, we decided that it is not appropriate to put forth a hypothetical evolutionary history herein and have thus decided not to include it in the figure.

(29) Line 230.
"substantial." (Add "l.")

Great catch, thanks!

(30) Figure 1 caption.
"the general tetrapodomorph sarcopterygian condition"
"General" is not appropriate wording in evolutionary biology. Since it refers to the charactar state of the common ancestor, it should be called "ancestral." Also, the expression "tetrapodomorph sarcopterygian" is redundant. Therefore, it should be rephrased as "the ancestral tetrapodmorph condition" or "the ancestral condition of the tetrapodomorphs."

Good points, changed to “the ancestral tetrapodomorphs condition”.

(31) Line 499.
"at a span of 4 cm" should be "at spans of 4 cm."

I don’t believe so, since this is referring to the spacing of the supporting pins (singular) rather than to the axial column tissue that would span the space during testing (multiple).

(32) Supplementary Table 2.
Positions 1–8 are confusing, so the positions should be shown on the body silhouette.
When you have the fish in your hands, you can feel the ribs and determine the precise placement for the descending point. I have added the following to the table heading: “Ribs are palpable for most of the anterior axial column after dissection, but measurements are used posteriorly where ribs are not palpable.” I worry that it might be more confusing on a body silhouette, but I have also added a small diagram showing the approximate arrangement of test positions.

Tatsuya Hirasawa (The University of Tokyo)

Referee #4 (Remarks to the Author):

General comments: This manuscript uses mechanical experiments on a modern analog and

modeling data from fossils to address a fundamental question in the evolution of the tetrapod body plan: when did a truly mobile neck evolve? The approach the authors use is relatively simple, and is clever in its simplicity. They first use being experiments on modern sturgeon vertebral columns, which have a notochord-dominated morphology analogous to that observed in early tetrapods, to gain insight into the level of flexibility of the notochord itself as well as the column including bones and connective tissues. They then apply these values to models that simplify the shapes of the neck region of the early tetrapods and calculate the degree of bending that would be possible given estimates of the amount of force that the muscles acting on the neck could generate. There are definitely some simplifying assumptions in terms of the modeled shapes of the vertebral columns and the the way they estimated the amount of muscle available to move the necks, but the assumptions generally seem reasonable and I suspect that their estimates are reasonably accurate.

Most of my comments below are relatively minor requests for clarifications or suggestions for small changes to the text and figures. Among these, maybe the most significant is the request for more information about what actual specimens were used in making the neck reconstructions for the fossil tetrapods. I also had one more significant question, specifically about whether bony stops (i.e., disarticulation of vertebrae where zygapophyses are present and/or bone-bone contact that would prevent further movement) would further constrain the ranges of motion they estimate. In part this question pertains to the sturgeon experiments (i.e., were there any cases where bone-bone contact seemed to be preventing further movement). It also pertains to the fossils, though, and is likely more important there because many of the fossils have more extensive articulations between vertebrae than is the case for the sturgeons. Given the very limited ranges of motion calculated, my guess is that bony stops are unlike to be much of a constraint, but I think this is an important thing to mention explicitly as it would speak to the overall plausibility of their results. Alternatively, it would be interesting to note if there are any cases where the size/orientations of articular surfaces suggest a range of motion that is greater than that suggested by the bending studies because this might suggest their results are overly conservative, although my guess is that's probably not the case. Finally, given that they have ranges of material property values for the extant sturgeons, it would be good to provide range of motion estimates for the fossil taxa that take this variation into account (e.g., range of motion estimates using, minimum, maximum, and mean values). That would provide a useful estimate of (one aspect) of uncertainty associated with the results.

In order to address the 'bony stops' question fully, instead of having it fragmented between the responses to line comments on lines 86 and 202 below, I'll write it in here. I have still included less detailed responses below, so there will be some repetition between this and those two line comments.

Pertaining to the sturgeon experiments, no we did not bend the columns far enough that this was a concern. We watched each trial carefully so that any trial where the specimen fell over could be removed and did not see a point at which hard stops were encountered. You can actually see this in the raw UTM data, the stress-strain curves have the appearance of a steady wave and would have likely shown a spike at higher degrees of bending if resistance jumped with an event like arches colliding.

For the fossil taxa, your guess is correct. Even in *Whatcheeria*, which has the largest estimated mobility, if the total bend was only happening at points between the vertebrae that would still only correspond to 4 (if counting it as if there are 8 joints, those between the seven cervical vertebrae and the skull plus first trunk vertebra) to 5.3 degrees of deflection (only

joints between subsequent cervical vertebrae) per joint, displacements at the lower end of ranges of motion measured between individual vertebrae in joint-based systems used to validate the Autobend method (Jones, Brocklehurst, & Pierce, 2021, *IOB*). The measurements I am referring to there-in were all on amniotes and relied on parameters like degree of zygopophyseal disarticulation to define. Given this, we anticipate that estimates based on bony stops in joints would overestimate mobility compared to our method were they possible for these vertebral anatomies. We strove herein to make a maximum estimate in each case (highest muscle pressures and maximum muscle areas used for example), so higher ranges of motion limited by interactions between the arches shouldn't be feasible, they could only further limit mobility.

Looking into your point on what kind of ranges of motion might be anticipated based on zygopophyseal angles, this is unfortunately impossible to make this kind of assessment for the taxa that have more extensive zygopophyses as they are not represented by 3D data sources like *Tiktaalik* is. I hope the discussion in the previous paragraph of this response will be sufficient reasoning on this point.

Range of material values for sturgeon – CMD has output a new version of the stats data to get precise readouts of the maximum and minimum E values for each of these, so they don't have to be eyeballed from the graphs. I have added these columns to extended data tables 9, 10, and 11. I have also added a sheet to the same excel file that has the scaled bend per 10 cm and the lungfish validation sheets. I have left the values for the maximum muscle pressure on the minimum E & G sheet, which is the combination that gives the largest possible estimates. I have left the values for the minimum muscle pressure in the maximum E & G sheet, which is the combination that gives the smallest possible estimates.

Overall, this was an enjoyable paper to read and the results are interesting. It was also nice to see that the unusual neck proportions of *Whatcheeria* that were described a few years ago seem to have real functional implications.

Yes! As BKAO said when I first sent over the estimates – "Head banging in the Holkerian lessgoooo"

Line 46: Maybe say soft tissue notochord instead of just soft. Regardless of the results you present here, I think it's generally known that the notochord is a somewhat rigid structure, and I don't think you want to imply that it is highly squishy.

I have added the word tissue, good point!

Fig. 1: Are the schematic drawings of the heads/necks in parts a-c to scale? It would be good if they are because it would help support your statement in the text that the lake sturgeon is of comparable size to the relevant tetrapods. If the drawings are to scale, please note this in the figure caption.

These are not drawn to scale. Other larger changes to the figures have been made as I work through these comments, but I have kept this in mind while making those changes!

Also, since you mention supra-neurals, basidorsals and basiventrals in the text, I think it would be good to use those terms in the labels for the vertebra in part E so that readers can match the names with the structures.

Due to other changes, these diagrams have been cut and are now only shown in Extended data figure 6. I have added these terms to that figure. The cartilage right above the notochord is just the base of the neural arch, the slice doesn't happen to have the basidorsal visible.

Line 86: From what you say here and in the methods, it seems like the way you're approaching the problem is to estimate the stiffness the notochord/axial column of the fossil taxa and then predict how far the column could be bent given the muscle force available. That makes sense to some degree because in general there would be few bony stops that could limit bending in the sturgeon, so essentially you're carrying over the assumptions from the sturgeon tests to the tetrapod models. However, in the sturgeon experiments are there any points where the column can bend to a degree where contact between bony parts of successive vertebrae come into contact and therefore could actually serve as bony stops on range of motion? Or is the degree of bending required for that to happen so great that it is beyond plausible limits? Likewise, when thinking about the fossil taxa, some of them have relatively closely spaced central elements and zygapophyses that could function as bony stops on range of motion that are more akin to what's seen in more crownward tetrapods. Did you take those into account at all? Similarly, are the ranges of motion you predicted small enough to avoid causing disarticulation or unrealistic collisions/overlap of the zygapophyses?

Your read on our approach is correct.

For sturgeon, bending laterally enough to cause contact between the cartilaginous vertebral arches would be implausible and rotation would not result in contact between the vertebrae either. In terms of extension (dorsal elevation), this would be a bigger concern, but extension is even more highly limited by the overall morphology so it is not a concern.

For the more crownward tetrapods, we expect that the addition of boney stops could only decrease the predicted range of motion, just like the addition of other tissues around the column such as integument, etc. Since all of these additional factors only decrease the mobility estimates and because zygapophyseal connections are only applicable for some of the taxa examined herein, we did not check for these limitations. That said, we only had 3D bone models of the type that would be necessary to perform a virtual bending to check for this from *Tiktaalik*, which is one of the least applicable to your comment unfortunately. In *Tiktaalik*, though, we can say that bony stops (considering the ossified vertebrae more posterior in the column), would occur only at a much greater flexion than we have estimated.

Line 107: The reconstructions in the supplementary figure look good, but I think it would be helpful to include more information (presumably in a section in the supplement) that explains which specimens form the basis of the reconstructions. I realize that a lot of the information underlying them is taken from the literature, but even in those cases, I think noting (as much as possible) which specimens were used in the literature sources would help readers feel confident that the different parts of the vertebrae are scaled accurately relative to one another, understand if there is articulation information that helps to estimate spacing between vertebrae based on articulated specimens, the degree to which the cervical series is based on an actual series of vertebrae vs. repetition of a single vertebral morphology, etc.

Excellent point, and I actually have this information in an excel sheet already that I have been keeping for my own reference. Added this information as Table S12: "Supplemental Table 12. Sources for vertebral reconstructions (Fig. S6-S7) and skull measurements (Fig. S8)." Turned off track changes while building this table so as not to clutter the extended data file, but it is tracked with the addition of the table name.

Line 176: It would be good if you can provide some measurement of body size here to help contextualize the neck lengths you give.

Elsewhere in the paper, I compared neck length to skull length as a proxy for body size. I have repeated the *Whatcheeria* measure here (neck length 89% of skull length) and measured and added the *Trimerorhachis* measure (neck length 19% of skull length) as well.

Also, neck length values and angular measurements of bending aren't given in table 1. It seems like you should call out table S5 here instead, which does provide those values.

Good catch, I have changed this reference to S5 and, after finishing the rest of the revisions, double checked all table and figure reference numbers.

Line 202: This would be a good place to discuss whether you would expect any impact of bony stops on range of motion for any of your included taxa, and if they were likely to come into play with the ranges of motion you calculated from shape/material properties alone.

I have added the following to the end of this paragraph of the discussion to note that boney stops could become a consideration in more crownward taxa: "The zygopophyseal connections between vertebrae are elaborated in crown tetrapods, as well, and these boney articulations could impose further constraints on axial column mobility towards the tetrapod crown that, like constricted notochords, would require an additional method to assess." I think it makes for a very suitable transition point into the following discussion on additional constraints on range of motion.

Line 228: The wording here make it sound a bit like it's implying that the head/neck is being used for propulsion. I recommend replacing propulsion with locomotion.

True, changed as suggested.

Line 244: You could call the neck a spandrel in this context if you want to cite Gould and Lewontin.

I appreciate the suggestion but have decided not to since we're already pretty far over the citation limit.

Line 485: How does the size of the yearling sturgeon compare to the early tetrapods you're studying? i.e., are they comparable in size at that age, or do the fish have to grow more to achieve tetrapod-like sizes?

Older sturgeon of this same species achieve body sizes like those of stem and early tetrapods, and can get really big to the point where they would exceed the size of most early tetrapods. The yearling fish are smaller, up to about half a meter in length. The thing that was important to me here is that they are beyond the first few months where 1. The notochord

would have an adult-like arrangement rather than a stacked cells arrangement, as it would in very young individuals (probably only a consideration for pre-hatch or near-hatchling sturgeon) and 2. the body structure is quite different due to a very heavy armoring created by the osteoderms which are disproportionately large in very young sturgeon compared to their coverage in adults. The yearlings are mature enough that they don't have either of these challenges. So, they are at an age where they move in a very similar way to the adults that reach 'early tetrapod sizes'.

Line 538: Was there an original source for the drawings that were modified (e.g., from Coates' paper)? Same question for *Trimerorhachis*.

Yes, these references are in the caption of supplemental fig. 7 and the new supplemental table 12 with full citations in the extended data references. Added "See figure S7 and table S12 for references." To this section.

Line 582: Was a certain specific vertebral position used for the 'anteriormost' column? Or average values from a series of positions? Are these the positions you mention at line 588? If so, I would recommend moving that sentence up to this line.

Sort of. Rib positions were palpable, so the testing positions were chosen based on counting ribs. So anteriormost is referring to the area of the vertebral column with the first 12 ribs. Added "(Table S1, Bending Position 1 (bending) and Section Number 1 (torsion))" to the end of this sentence. These are the same as mentioned in line 588.

It occurred to me while looking at line 588 that it would be good to specify that I am counting "posterior to the midline skull" to mean both the braincase and the fused vertebrae that sturgeon have, so I have added "and fused occipital vertebrae" to that sentence in addition to moving it up in the paragraph as suggested.

Referee #4 (Remarks on code availability):

There is a README file (code demo), but there wasn't code provided with the documents for review. I also didn't see a link for a repository with code, but maybe I missed it.

You would have had to go to the github repository to get the code, as far as I can tell it was not directly included in the review packet. Another reviewer and their co-reviewer were able to get and run it, so I hope that is satisfactory.

Additional Changes – I noticed during preparation of the lungfish validation excel sheet that I had changed the notochord radius column to be for the notochord diameter in the rotation/torque calculation area for my original spreadsheet but still had the torsion calculation written as if a radius was the input, so the diameters were being doubled in the calculation. I have fixed this in the calculation sheet including the one that I am attaching to this review and have changed Extended Data table 6 to reflect this. This did not impact any of

the main or extended data text, it only further shows how little stiffness of the column is contributed by the notochord.

I also had to cut a few words from the abstract after incorporating edits, and aimed to do this in a way that did not change the meaning or flow.

Dear Dr. Gee and Reviewers,

Many thanks for your handling and careful checks on the revisions to our manuscript, “Early tetrapod neck mobility modeled from an extant analogue.” I am pleased that we have satisfied most of your comments in the first round and grateful for the time you all have taken checking over the edits.

To Referees 1 & 2, I will keep in mind the future possibility of sourcing lungfish from the pet trade if I come up with a project that needs them! Aside from that, I don’t have anything to add for you two in responses below.

To Referees 3 & 4, you will find my responses to your comments below. As before, I have greyed out the original text of your comments and offered my responses in purple below each comment. I really appreciate both of your input and attention to detail in this second round. To Referee 3, where my first edits and responses failed to satisfy, I hope you will find that the changes we have made in this round do. I have consulted with a number of my co-authors specifically on the comments regarding precision of measurement throughout the manuscript and I believe that has helped me come to a much better understanding of the edits needed to address your concerns. You were right to observe that these changes do not affect the results, but there have been many minor adjustments to numbers in the extended data figures and tables.

Dr Gee, I am aware that we have added a number of citations beyond the preferred number during revision but in all else I have endeavored to abide by the author guidelines throughout this process including fixing reference formatting in the extended data in this round. If we must choose some cuts from the introduction, please let me know.

As there were very few comments this round and most were minor, I have also taken the opportunity in this revision to check the flow of the writing and improve it in places, making previous additions and cuts more seamless to improve the readability, especially in the early sections of the discussion. So, there are some additional changes for style in this version not enumerated in the point-by-point below that are visible in the tracked changes version. These are stylistic and should not change the content of the manuscript.

Best wishes,

Dana E. Korneisel

on behalf of the team

Referees' comments:

Referee #1 (Remarks to the Author):

Overall, I appreciate the authors' thoughtful and positive approach to the reviews. They engaged meaningfully with my comments and made thorough revisions as a result. All of my original concerns have been sufficiently addressed and I do not have any further concerns or improvements.

The use of existing lungfish data (Gartner et al., 2022) is a clever way to validate the methods on in vivo data from extant species. Lungfish are available through the aquarium trade, so future studies would be able to acquire fresh (unfixed) specimens -- if scientifically and ethically justified.

The revised terminology for head, trunk, and neck motion provides more clarity, while avoiding the tricky issue of how, exactly, to define these regions (which I agree is a topic for a separate study/paper).

Referee #1 (Remarks on code availability):

As previously reported, the code can be installed and run. The improved comments and README file now make it much easier to use and understand this code.

Referee #2 (Remarks to the Author):

I co-reviewed this manuscript with one of the reviewers who provided the listed reports.

Referee #2 (Remarks on code availability):

The provided code works as intended and can be used to reproduce results.

Referee #3 (Remarks to the Author):

The revised version of the manuscript entitled "Early tetrapod neck mobility modeled from an extant analogue" by Dana E. Korneisel and colleagues has been improved from the previous version. I was mostly convinced with the authors' response to my comment. However, there still remain several points to be clarified, particularly concerning numerical values in primary data and significant figures, before publication.

Major comments

(1) Significant Figures of the Data
The authors' response to my previous comments was insufficient.
The neck length and notochord diameter of fossil species are described to have been measured at a resolution of 0.01 mm. It should be considered whether it is truly possible to measure objects on the scale of a few centimeters with this level of accuracy using photos and CT data, and if so, the method should be explained. When measuring from photographs, the angle of the specimen and the effects of lens aberrations must be taken into account, but it seems extremely difficult to obtain data indicating that "the notochord diameter of Acanthostega is 1.526 cm", for example.

I am sorry to hear that our initial response did not satisfy. This was actually the final revision comment that I was working on and considering even throughout the process of collecting data for the other revisions because, while I understood your concern, what I perceived as the suggested action was essentially choosing an arbitrary decimal place and rounding off all of the measurements. To my mind, there are so many factors in these measurements that are going to affect their accuracy that the best thing to do is to explain where the measurements come from but then faithfully represent the digital measurement, acknowledging the potential error sources. After reading this new comment a few times, I decided we had our wires crossed somewhere and consulted with a few of my co-authors for their thoughts. I hope that their added perspectives have allowed us to come up with a reasonable response.

On EMS's recommendation, we have determined that the resolution of the reconstructions is the most relevant limiting factor to any measurements on these reconstructions. As these are presented with mm scalebars, this means that measuring to the tenth of a mm is our acceptable limitation to precision. I have gone back in and repeated a few of the measurements to see whether this corresponds well to the human error component and found that measurements to the tenth of a mm were typically stable, giving me some confidence that this cutoff is, in fact, appropriate.

This does not apply to the measurements on *Tiktaalik*, which are on a CT reconstruction with a scan resolution of 122.441 um, so X.XXX cm corresponds well to resolution and is used there (no change).

I have checked over the main methods section and made a slight modification for clarity (since three of the reconstructions are the same, modified from literature, I removed the extra sentence repeating all the same for *Trimerorhachis* and just moved it into the list above).

To the extended data methods, I have added the information about the *Tiktaalik* scan resolution. I have also added a note that reconstruction measurements were to the nearest tenth of a millimeter and moved down the parts that were added in the last

revision so that information about these measurements doesn't break up the description of various measurements on the CT model.

Lastly, I have checked that these are represented in the mobility estimates file as well as extended data tables 5-8 and checked for any adjustments to the estimates caused by changing the measurement inputs. Intercentral span was in the place of notochord diameter in a number of places and this was replaced. In all of these tables, neck length was adjusted. The change in neck length values did not impact the whole column estimates of maximum bending but torque changed slightly, and this has been changed to match in table 6. In tables 6 and 8, notochord diameter was updated. In table 6, the adjustment for notochord diameter rather than intercentral span contributed to the change in torques and, likewise, in table 8 this resulted in a change in bending angles so both of these have been updated. In table 7, the title of the notochord column was changed to intercentral span and that was input directly.

Furthermore, as is evident, in Extended Data Figure 8, attention has not been paid to the significant figures upon calculation. Although they do not affect the final conclusions of this study, clearly stating the level of precision used in the research is essential in scientific papers.

You are correct here and I have no qualms with matching each of these areas to the smallest number of figs from the input measurements, so I have done so and changed the figure accordingly. These inputs are generally reconstructed with cm scales so I have taken these measurements to the nearest mm. You will notice that I have kept the measurements from *Tiktaalik*, not from a drawn reconstruction, at the tenth of a mm, well within what the scan resolution allows. I did not originally set the degree inputs to get the exact muscle areas down to decimals to match due to the broad estimate nature of these muscle areas, but I have checked these adjustments against my modeling document just in case any issues appeared. I did not find any.

These area calculations were done by hand but most of the other calculations in this study were performed in R. I have checked the values presented in table 1 and found none are reported to more than 3 significant figures. Measurements in extended data table 3 were done by hand with measuring tapes, and are reported to the appropriate level of precision for this method. Extended data tables 5-8 edits are discussed above. Extended data tables 9-11 are meant to report raw results (essentially intermediate calculation steps) and I have not made any changes to them. I have rounded one number in extended data table 13 in addition to the Extended data figure 8 values.

The min and max sheets created for the last round of revision have also been checked, all changes were so minimal as to not be meaningful to the results.

In addition, while I understand that the zwickiLine Z5.0 TN used in the test is extremely accurate, the measurement accuracy should be described in the "Methods: Material Testing" section so that readers can understand it.

We had to check each of these force transducer specs with the company because these differ for the two load cells (the one used for bending and the one used for torsion). Having seen how much trouble it was to get this from the manufacturer going through someone who owns one, I do think it is worth putting in! Here is what was inserted:

"Tissues were measured on a on a zwickiLine Z5.0 TN materials testing machine (ZwickRoell LP, Kennesaw, GA) using a Zwick/Roell GTM Series K load cell (5 kN capacity, ISO 7500-1 Accuracy Class 1 from 20 N - 5 kN) for bending. Measurement electronics provide a 24-bit resolution over the range corresponding to a force resolution finer than 0.01 N; while our max loads fall below the ISO threshold, they remain well within the system's electronic resolution and are repeatable across measurements. Torque was measured using a 20 Nm torque transducer (ISO 7500-1 Accuracy Class 1 from 0.2 Nm to 20 Nm) on the same machine. The full-scale range corresponds to a resolution finer than 0.0001 Nm; while some of our measured torques fell below the ISO threshold, they similarly remained well within the system's electronic resolution."

This is at the end of the opening paragraph of "Methods: Material Testing" as suggested.

Minor comments

(2) Line 133
Remove "degrees."

Thanks for catching this, it is removed.

(3) Figure 1
Fig. 1 in the new version is improved. However, there is a problem with how the ranges of taxa are illustrated on the phylogenetic tree. In the current version, all taxa are apomorphy-based, but the definitions of some taxa are branch-based (e.g., Tetrapodomorph). On the other hand, "Tetrapoda" is usually defined apomorphy-based, but based on that, it also includes stem tetrapods such as Acanthostega. The closest phylogenetic position to what the authors labeled would be "crown tetrapods". However, if "crown tetrapods" are to be illustrated, they must be labeled at the branching point of the common ancestor of amniotes and amphibians.

Thank you for this commentary, that's a fair consideration. I have gone back and forth a bit on whether having a "Tetrapoda" and a "Crown Tetrapods" label would be overdoing it, but I think it is better to do this than just to move the label, so I have moved the Tetrapoda label to 'below' *Acanthostega* and changed the label (slightly scootched from the current position) to read "Tetrapod Crown", not in bold to reflect the current choice in the figure to only bold formal taxonomic terms.  As follows:

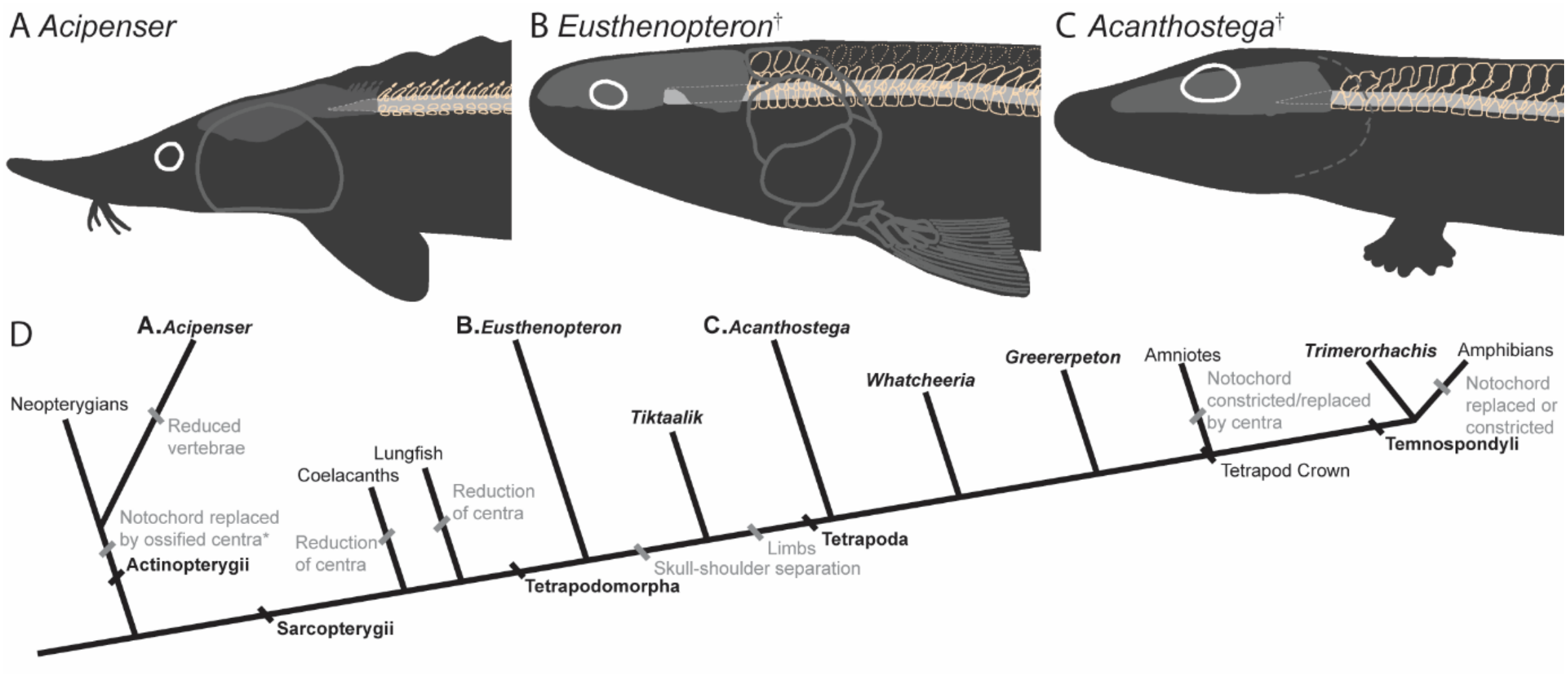


While doing the first round of revisions, my computer had appeared to have died and I had to do the edits directly on the first-round submission tiffs. Luckily, it survived so I was able to edit the original files this time. I have looked between the versions to check for deviations but I believe I have gotten them in line with one another except for the additional edits you suggested here and one more edits. I had wanted to rearrange *Greererpeton* and *Whatcheeria* on figure 2 last time and was able to do that neatly now so that the layout in fig 2 is consistent with the phylogeny in fig 1.

In panels A–C, only B. Eusthenopteron shows the shoulder girdle and fin skeletons; however, for consistency, the corresponding skeletal parts should also be shown in panels A and C.

I agree that adding shoulder girdles and limbs to panels A and C would make them more consistent with one another but I don't agree that this would improve the figure. The added drawing would detract from the relevant details to the discussion in this study and make the vertebrae harder to see (as you noted in your first round of revisions, this is already a difficult balance in this figure!). We don't want to obscure the relevant details. In this particular case, I think the most important things to show are

- in panel A, that the sturgeon has some occipital vertebrae fused into its occiput
- in panel B, that the opercular series etc overlaps the first few vertebrae in *Eusthenopteron*
- in panel C, that there still are some lateral bones that extend posterior to the occiput in *Acanthostega*, indicated by the dashed line

Adding more limb and girdle bone outlines would detract from these. I have checked the figure caption to make sure that the differences in the three depictions are described there.

(4) Extended Data "Measurements" section and Extended Data Figure 7
I understand that the method of identifying the cervical vertebrae in this paper has become clearer from the authors' responses to previous comments. However, I believe that Whatcheeria's explanation needs to be more specific, as shown in the response, rather than simply stating that it is based on "personal observation."

I am glad to hear that this clarified things for most of the taxa. I have looked back at your initial comment as well as my initial response in considering this *Whatcheeria* question. I can definitely see how you getting more explanation doesn't satisfy the need to clarify in the manuscript, so apologies for that explanation without a matching edit last time. I have added the following to the figure caption for extended data figure 7: "modified from Otoo *et al.* (2021) by removal of the first centrum component from the original reconstruction following Korneisel *et al.* (2025)'s observation that this appears to have been a misplaced pleurocentrum".

I also added the following to the parenthetical in the Measurements section that references extended data figure 7: ", note that the reconstruction of *Whatcheeria* is modified from the original as described in the figure caption". I think that this satisfactorily communicates that we followed BKAO's definition of cervicals for *Whatcheeria* except for this modification and hope that you will agree.

Referee #4 (Remarks to the Author):

General comments: It's good to see this paper progressing through the review process. Overall, I'm satisfied with the authors' responses to my comments and the changes they made to the manuscript to address them. I have a couple suggestions where maybe adding a sentence or two would further help to show that bony contacts don't play a major role in determining range of motion, but those should be very simple additions. Other than that there were a couple places where I think changes didn't get carried over as intended between drafts exactly as intended (e.g., the caption to figure 1), but those are just technical changes to make.

Figure 1 caption: The ordering of images of taxa in the figure and the A/B/C lettering described in the caption don't match up. It seems like the ordering from the previous version of the figure was inadvertently carried over.

You are absolutely right! I have reordered these to match the new figure layout.

Extended data figure 7: It didn't occur to me in the previous round of review, but the presence of Proterogyrinus and Archeria in this figure isn't really explained anywhere. Those taxa only show up in this figure and aren't referred to anywhere else in the main

text or supplement that I can find aside from an oblique reference at line 213. I think the space that they use in the figure would be better spent on showing vertebrae for Eusthenopteron and Tiktaalik considering that they are discussed a lot in the text. I realize they show up a little in some of the other figures, but having them shown in a comparable way to the other taxa here would be helpful. If you do want to keep Proterogyrinus and Archeria in an expanded version of this figure (which also includes Eusthenopteron and Tiktaalik), then I recommend more explicitly referring to them somewhere in the text (maybe around line 51, where you talk about taxa where the vertebrae are more ossified and the notochord is reduced, and also specifically naming them when you mention embolomeres at line 213 to help readers who are unfamiliar with early tetrapod taxonomy make the connection between them).

Yeah, the embolomeres should be removed. I had kept them throughout because of those few statements, so the reader could see what I mean by constricted notochord, but this is probably pretty obvious and not necessary to figure. I have cut a few words in lines 211-216 including the reference to those panels in order to make this change fit rather than elaborating more in the text in order to keep them. I have also deleted the two Holmes citations from the extended data file.

On Extended Data figure 7, we have done as you suggested, cutting the embolomeres and adding the other two taxa used in this study. I have also rearranged the figure caption to match. I have included a note on both of these additions to clarify that measurements come from another source for them.

Line 85: Change to: All contribute to the material properties of the column.

Done as suggested.

Line 97: You comments in the response to reviewers about bony stops not seeming to serve as a strong constraint in bending makes sense. I still think this is an important point to mention in the paper, though. This (line 97) seems like it might be an appropriate place to add a comment that you didn’t observe this limiting bending in the sturgeon case. Somewhat related to this, you could also note that checking the the 3d orientations and sizes of the joint surfaces of those taxa would be an interesting next step; i.e., are the joints sized and shaped in a manner consistent with very limited ranges of motion? It would be a good test of your material properties/basic shape-based predictions. Line 216 seems like it would be a good place to include that discussion point.

I am glad to hear that the initial response was mostly satisfactory. I did some rearranging around line 97 to make this inclusion read well and have the following in now: “This high stiffness is not attributable to hard stops or joint-interactions between the arches, as we did not observe any such interactions nor any regionalization of bending during testing.” As I see it, there are two important points you raised that I want to address with this statement, both

the lack of bony stops and this not being a joint-based system. I have tried to choose phrasing that matches to the other content of the manuscript rather than bringing in new terms from what we've discussed in this revision process.

For the second part, I had already taken out a few words about embolomeres based on the last comment. I lost a bit of the flow of this section while rearranging the information, so I have changed the wordings around for style, adding the context you have suggested about joints. It now reads: "In other taxa like embolomeres and amniotes, this widening is also correlated with increasing replacement of the notochord by vertebral centra, putting estimation of their range of mobility beyond our current method. The zygopophyseal connections between vertebrae are elaborated in crown tetrapods, as well, adding complex joints to the vertebral column. Evaluation of early tetrapod and early amniote axial columns with more developed zygapophyseal articulations will require another novel approach."

The question of how to model mobility in joint-based systems where the major joints are between vertebrae but there is also potential mobility between components within each individual vertebra is one of my white whales of research. I tried to convince one of Katrina Jones' students to take this on as an update to an autobend-like mobility approach. I certainly don't mind mentioning it again, and hope someone will read this and think of the solution to this puzzle.

Line 222: It might be interesting to mention the young sturgeon example you referenced in the rebuttal as an example of how the presence of osteoderms could significantly impact mobility (especially since at least some of the taxa in question did have bony scales, even if they weren't exactly like the osteoderms of sturgeons).

True. I heard about the low mobility of young sturgeon first from the fishers who I sourced my first fish from (that I used to sort out the testing set up). The 'literature' on C-starts using very small fish where you can see this is pretty disappointing, though, it comes down to a choice between citing dissertations or a preprint from 2020 (https://www.biorxiv.org/content/10.1101/2020.03.01.972257v1.full.pdf) that I am afraid may have been abandoned. The preprint doesn't consider the growth of the osteoderms, it just has a few images that make the stiffness of the juvenile body really evident if you have also seen yearling and adult fish bend!
I would like to include this anecdote, because I agree that it is interesting, but due to the lack of good citable material for an added statement I have chosen not to take this comment, sorry. I think it would be a fun topic for a future study if someone has a bunch of fish on hand, it probably has implications for shifts in the ecology of these animals throughout their lives.

Line 486: Is 'material characterization material testing" redundant?

Yes, it is. This was meant to be a change from the term "testing" to "characterization". I have removed the former.

Line 643: Should 'depending' be in the sentence? Or is something missing after it?

It should not be, that is a leftover word from my having previously written "depending on the proportions of the individual". It is now removed.

Dear Dr. Gee and editorial team,

Thank you for providing these comments on the formatting and templating of our manuscript, “Early tetrapod neck mobility modeled from an extant analogue” and my sincere thanks to the statistics reporting team for their careful review of that document. I have now completed the edits that I believe address the stated concerns, with considerable help from my second author, Cassandra Donatelli, who has cleared up some confusion on my part regarding the github repositories and provided most of the responses relevant to the stats, code, and math below to make sure it is all correct and reported correctly. You will find detailed responses below to the formatting edits, final comments from Reviewer 3, and Reporting comments on a point-by-point basis describing the fixes we have made to address your comments.

Responses to referee 3’s final comments are between the editor responses and editorial requests document responses. I have not addressed this letter to Dr. Hirasawa on the understanding that he is not reviewing these edits again, but my apologies if that is not the case. In short, I have agreed to the argument for rounding the *Tiktaalik* measurements which did move the estimate by .25 degrees. This has been updated but does not influence our interpretations.

I have also had another go over the full formatting guidelines list and hope that anything I have failed to catch is minor enough as to not be a terrible bother. I could remove some of the introductory citations if the 50 limit is insisted on, but I do feel it would be arbitrary. The word counts are as follows: Summary paragraph/abstract = 201 words, Main = 3,089 words, Methods = 1,843 words.

I really appreciate your communicativeness and flexibility throughout this submission and revision as well as the whole team’s attention to detail. Working through the review process on this manuscript has been at times difficult but also certainly positive. I know that it has improved the manuscript greatly. As before, the itemized responses are below in purple!

Best wishes,

Dana E. Korneisel

on behalf of the team

Referee #3 (Remarks to the Author):

The authors responded sincerely to my comments. In particular, the accuracy of the measurement equipment has been thoroughly investigated and described in the Methods section; this study represents a significant contribution that will serve as a valuable reference for future research. However, I believe there are two points that should be addressed prior to publication. In all other respects, the manuscript is an excellent scientific paper.

Minor comments.

(1) Methods, Measurement section in the Main Text

Lines 546–547.

"Each intercentral span measurement was divided by 0.96 to estimate the notochord diameter" should be changed into " Each intercentral span measurement was multiplied by 0.96 to estimate the notochord diameter." (Notochord diameter = intercentral span x 0.96)

I can see why you would expect the relationship to be this way, but this is not quite right. The notochord is a bit wider than the intercentral span in the sturgeon, as the intercentra are positioned ventrally and do not quite reach the maximum width. So, 0.96 x notochord diameter = intercentral span in sturgeon.

That said, I have had a close look through everything because of this comment. After taking a closer look at the axial column cross-sections, the only taxon that might have a sturgeon-like wider notochord than intercentra is *Tiktaalik*, but even in that animal the intercentra are quite tall laterally. All of the rest of the taxa herein have fully bound lateral extents of the notochord. So, the intercentral span to notochord calculation probably only applies to sturgeon in the end. See extended data figure 6 to see the difference (dashed vs solid line in sturgeon cross section compared to the solid line that is both intercentral span and notochord diameter in *Tiktaalik*).

I have removed this sentence from the section and added “in *A. fulvescens*” to the in-figure text of “extended data figure 6” (now extended data figure 4). I have also taken out the

calculation, instead just using the intercentral span measurement and checked all the notochord values for any changes that this would make, reverting Extended Data table 8 to a no-extra-calculation version. This makes no changes to the interpretations presented herein.

(2) Significant Figures of the Data

In some cells of Extended Data Table 7, the significant figures have not been corrected.

Correct, I missed the base and height columns in this table. Those are now fixed to match with the measurements reported in the other tables. I have also had another go over the tables to try to find any other mistakes.

Furthermore, given the CT data resolution of 122.441 µm for *Tiktaalik*, a measurement should be expressed as "1.59 cm" (X.XX cm) rather than "1.585 cm" (X.XXX cm). This is because measurements on digital images are typically taken in pixel units (unless specialized techniques are employed); even assuming no manual error in pixel selection, the result falls between 129 pixels × 122.441 µm (1.5794889 cm) and 130pixels × 122.441 µm (1.591733 cm). Consequently, the second decimal place falls within the margin of error, rendering the third decimal place meaningless. While this applies to two-dimensional measurements, measurements in Blender—compared to those in ImageJ (Fiji)—are subject to three-dimensional misalignment and interpolation (smoothing) during 3D model rendering; therefore, one generally cannot expect accuracy superior to that of two-dimensional measurements.

I see your point and have dropped this final digit. As with the changes in the last round of review, I have checked through the deflection calculations to make sure that this rounding does not make any changes to the returned estimates or, from those, the interpretation. The dorsoventral estimate had to be edited to 2.25 rather than 2.5 degrees, not sufficient to make any changes to the interpretation. I edited this value in line 209 in the main text as well as Extended Data Table 5 and Main text figure 2.
